# Supplementary material for: Medieval and early modern diets in the Polack region of Belarus: A stable isotope perspective
Source: PLoS One. 2022 Oct 7;17(10):e0275758. doi: 10.1371/journal.pone.0275758 (PMC9543997; doi:10.1371/journal.pone.0275758)
Supplement: S1 File — (DOCX) [file pone.0275758.s001.docx]

**Supplementary Information for**

**Medieval and Early Modern diets in the Polack region of Belarus: A stable isotope perspective**

Haponava et al.

**This file includes:**

S1 Note. The Polack region in the 11-18^th^ centuries: historic background.

S2 Note. Additional information on past diets in Belarus.

S3 Note. Further details about sites and contexts analysed.

S1 Fig. Example of a kurgan burial in Skrabianiec (11^th^ century).

S2 Fig. Example of a ‘stone’ pit burial in Pieravoz-4 (13-14^th^ century).

S3 Fig. Plan of the excavations of the ground cemetery near the village Biruli in 2011.

S4 Fig. Situational plan of the kurgan cemetery #4 near the village Pieravoz with the ground cemetery marked to the north of it.

S5 Fig. Plan of the excavations in Polack Lower Castle in 2014.

S6 Fig. Plot of δ^13^C and δ^15^N values of human and animal samples grouped by sites.

S7 Fig. Plot of δ^13^C and δ^15^N values of human individuals from the elite, military and rural burial contexts.

S1 Table. The stable isotope results of the human samples and the quality control indicators.

S2 Table. The stable isotope results of the animal samples and the quality control indicators.

S3-18 Tables. Results of the post-hoc tests.

S1 References.

**S1 Note. Polack region in the 11-18^th^ centuries: historic background**

The city of Polack was a major Medieval trade, political and cultural centre with masonry churches, monasteries, markets and prospering crafts [[7,9,22]](https://www.zotero.org/google-docs/?1bJoK1). Among the early states which together comprised Ancient Rus in the first centuries of the second millennium AD, Polack Principality was the most prominent of them on Belarusian lands [[9,20]](https://www.zotero.org/google-docs/?dpIfPp). Overall, the Ancient Rus is described as a vibrant community, with prospering trade which witnessed numerous military campaigns and territorial changes. Written sources like the “Polack Chronicle” discuss the confrontation of Polack with Novgorod and Kyiv rulers in the 12^th^ century, which included both alliances and joint raids against common enemies, fights against each other, and dynastic marriage [[13,21]](https://www.zotero.org/google-docs/?cNAp5p). Both the lives of nobility and ordinary people could have been affected by such political and social processes, wars often being followed by famines, epidemics and mass migrations.

Belarusian lands avoided occupation by the Tatar Mongols in the 13^th^ century AD but were involved in the fights against them as well as against the Teutonic Order. Against the backdrop of these circumstances a new state formed in Eastern Europe - the Grand Duchy of Lithuania with a capital in Navahrudak (Novogrudok). From the 14^th^ century AD, the lands of Polack also became part of the Grand Duchy of Lithuania (GDL). In the 15-16^th^ centuries AD, Belarusian lands were impacted by the European Renaissance, cities obtained the Magdeburg Law and craftsmen and merchants formed guilds. Secular education emerged at this time and reached its prosperity in the cities of the 18^th^ century AD [[22]](https://www.zotero.org/google-docs/?6t61b9).

In 1569 the GDL and Poland united into the Polish-Lithuanian Commonwealth (PLC), and the Polack region became part of yet another new state. The western Belarusian cities continued to develop economically and culturally, but many of the eastern cities fell into decline following the devastating wars of the 16-17^th^ centuries AD and the Great Northern War of the early 18^th^ century AD [[22]](https://www.zotero.org/google-docs/?kVkWFk). Polack was particularly severely depopulated and devastated after the Livonian war, leading to the decline of its role as a political and socio-economic centre [[9]](https://www.zotero.org/google-docs/?Zn874y). Territories in the west of the GDL suffered as well, with some settlements being almost completely abandoned following a series of depopulation events including the war of Muscovy with the Polish-Lithuanian Commonwealth in the mid-17^th^ century, an invasion by Sweden (1706), a nationwide famine (1708), and the Big Plague (1709–1711) which devastated populations not only in Lithuania, but in Poland and Prussia as well [[5]](https://www.zotero.org/google-docs/?mtR9yK). The deterioration of the situation in the Polack region culminated towards the end of the 18^th^ century AD during the partition of the Polish-Lithuanian Commonwealth by Austria, Russia and Prussia, which resulted in the annexation of Belarusian lands by the Russian Empire.

**S2 Note. Additional information on past diets in Belarus**

**Agricultural crops**

Agriculture was the base of the economy in Ancient Rus, as stated in written and archaeological sources and supported by linguistic analysis [[30,33]](https://www.zotero.org/google-docs/?uaTBSZ). The territory of Belarus is rather homogeneous with an average annual precipitation of 600-700 mm and average annual temperatures between 4.5°C in the northeast and 7.0°C in the southwest [[106]](https://www.zotero.org/google-docs/?e0KaoA). The climate in Europe from the first centuries AD is believed to have been roughly similar to the modern one. This moderate continental climate was beneficial for agriculture [[7,29,30]](https://www.zotero.org/google-docs/?ykwWy3). Soils were also similar to the current ones, being moderately fertile and suitable for agriculture. However, the sod-podzolic soils, sensitive to drought, were more widespread in the past, especially in the north-west of Belarus [[7,30]](https://www.zotero.org/google-docs/?w5OiWM).

Written sources for the Ancient Rus period give very little information about the agricultural economy of the time and its productivity. However, according to Halubovič [[7]](https://www.zotero.org/google-docs/?BYPSxI), years of crop failure and famine were relatively not frequent - for example, they happened 8 times between 1162-1250 and lasted 1-2 years. The main reasons for crop failures given were wars along with natural disasters. Among the years with bad harvest that brought famine to Ancient Rus, 1024, 1127, 1228, and 1230 AD are all noted [[30]](https://www.zotero.org/google-docs/?CV4MXq). For comparison, in the territory of Western Europe written sources name about 30 famine years for the 11^th^ century alone. However, this may be a consequence of the relative abundance of sources. Based on the chronicles from neighbouring Ancient Rus chiefdoms and much later written sources from Belarusian territory, scholars assume crop yields were rather high (with the ratio of the harvested crop to that sown being between 3 and 5) [[7,30]](https://www.zotero.org/google-docs/?rEUO1u), though Korobushkina [[30]](https://www.zotero.org/google-docs/?fg2uFT) states the average yield to be lower (2.5-3). Agriculture would have thus provided surplus product which was used in internal and foreign trade [[7]](https://www.zotero.org/google-docs/?IYRqOb).

Overall, in the 10-13^th^ centuries almost all currently known food crops were grown, except for those which were brought to Europe later from the Americas. Still, the written sources of this time are insufficient alone to reconstruct agriculture on the Polack territory in detail, as they only list the general use of certain crops like rye, wheat, barley, oats, buckwheat, millet, legumes (peas, beans, lentils), hemp, flax, and vetch [[30,33]](https://www.zotero.org/google-docs/?KW7WMT). Therefore researchers have relied on the study of archaeological materials, primarily charred grains and weed remains, to aid in the reconstruction of agricultural practices. For example, a study based on charred grains found in archaeological sites of the Ancient Rus dated to the 5-15^th^ centuries AD was conducted by Kiryanova [[29]](https://www.zotero.org/google-docs/?NT41OJ). She compared the relative frequency of cereals over centuries and distinguished the main grain crops in different periods. Based on the studied material, the most widely spread crop on the broader Ancient Rus territory in the second half of the 1^st^ millennium AD, directly prior to the period of focus of our research, was common wheat, with barley and small-fruited legumes also being prominent. Millet is also found on three out of seven sites of the period (and on one out of three sites from the territory of modern Belarus). Other finds include rye and oats (mostly in quantities insufficient to consider them stand-alone crops), peas, emmer wheat, vetch (weed), hemp, and flax. Rye and oats represent new grain crops coming into use in this period dominated by wheat and barley.

At the time of inclusion of the Eastern European lands into Ancient Rus from the 9^th^ century AD, farmers continued to cultivate grain crops known in the previous period. However, their ratio is different as rye became the main crop [[29]](https://www.zotero.org/google-docs/?wwaMUP). Wheat and barley occupied the second and the third places in the list of important grains. This conclusion is supported by Dovzhenok [[33]](https://www.zotero.org/google-docs/?w1kM2q), Korobushkina [[30]](https://www.zotero.org/google-docs/?F12ywd) and Bialiavina [[10]](https://www.zotero.org/google-docs/?FbuKQi) who state a special role of rye, which was more frost-resistant than wheat, in Ancient Rus and Belarusian territory in particular. The earliest find of rye specifically on Belarusian territory is dated to the 9^th^ century, though it appeared as a weed in the archaeobotanical collections earlier [[107]](https://www.zotero.org/google-docs/?qkXT1L). Only from the 9^th^ century is it recorded that rye was already cultivated as an agricultural crop, at least then a whole collection of grain is recorded rather than single grains. From the 11^th^ century, rye started to dominate other crops, comprising 50-60% of the finds in the northern-central parts of Belarus. In the southern part of Belarusian territory, finds of grains are more limited, but rye remains the most abundant alongside wheat as well as millets and lentils, some hemp and flax. The percentage of rye in finds grows in the 12-13^th^ centuries; in some sites it grows from 50 to 70-98%, especially in the south, in the Nioman (Neman) basin [[10,30]](https://www.zotero.org/google-docs/?Bm2Hq3). Finds in Homieĺ (Gomel, Homyel) (south-east of Belarus) are in line with this statement, as rye outnumbered the sum of all of the other charred grain finds in the closed storage complex dated to the second half of the 13^th^ century found in the city. Barley was the second most popular grain in terms of frequency. Oats and lentils were also found, as well as millet, but in small quantities [[108]](https://www.zotero.org/google-docs/?PM89Vh).

Despite the dominance of rye, wheat continued to be a leading crop along with it in the 11-13^th^ centuries, occupying the second, sometimes even the first place by the quantity among finds. The earliest finds of barley on the territory of Belarus are dated to the 10-11^th^ centuries [[10,30]](https://www.zotero.org/google-docs/?bUErj2) though, according to Loshenkov [[107]](https://www.zotero.org/google-docs/?FcMwKA), it was cultivated throughout the Iron Age from the 7^th^ century BC to the 9^th^ century AD. In the north of Belarus, it has been identified on the Krivel, Esmony and Novoye Selo monuments. Loshenkov [[107]](https://www.zotero.org/google-docs/?j7Dp6v) believes that it was in the Iron Age that barley "settled" on the territory of Belarus. Barley is the third most frequent crop in most sites of the Polack Principality and 13^th^-century Polack [[10,30]](https://www.zotero.org/google-docs/?P0VxVn). In Braslaŭ (Braslav, Braslaw; north of Belarus) it is even the second most frequent. Therefore, despite not being able to compete with rye and wheat, it was still grown in significant quantities in some places. Oats first appear among finds of the 10-11^th^ centuries and in small quantities in Polack and most other sites [[10,30]](https://www.zotero.org/google-docs/?rVGE2o). Based on Kiryanova [[29]](https://www.zotero.org/google-docs/?ypImeM), though, this new crop usually lagged behind rye, wheat and barley in terms of the frequency of crops grown in Ancient Rus of the time.

The above-described popularity of crops could vary from site to site, as in the city of Viciebsk (Vitebsk) in northern Belarus barley dominated the charred grain finds of the first half of the 14^th^ century (followed by wheat, oats, and only then rye) [[29]](https://www.zotero.org/google-docs/?shyxBv), and also was the only crop mentioned in a written source of the time [[37]](https://www.zotero.org/google-docs/?nqnuf9). Koledinskiy [[37]](https://www.zotero.org/google-docs/?49kpru) considers this only logical given the local soil and climatic factors which were highly suitable for this crop. Similarly, although overall considered second in importance to rye, wheat is mentioned as the main food product in some written sources - however, more so in southern regions [[33]](https://www.zotero.org/google-docs/?vkGfwO).

Legumes still remained prominent crops in the beginning of the second millennium, and more so in the western part of the Ancient Rus state (including Belarus) than in the eastern [[29]](https://www.zotero.org/google-docs/?HQzqTo). According to Korobushkina [[30]](https://www.zotero.org/google-docs/?IJw25i), legumes constituted a certain proportion of food crops - however, the author does not give any numerical estimation of this proportion. The legumes included small-fruited beans, peas, vetch, lentils, sweet pea (*Lathyrus odoratus*), and vegetable beans. In Polack, horse beans, peas, and sweet peas were found in the layers dating to the 11-12^th^ centuries. The proportions of lentil finds are variable in different sites, but usually remain quite small, between 2.5% and 15%. There are some suggestions that peas occupied second place in terms of crop frequency found in the 13^th^-century Vaŭkavysk (Vawkavysk, Volkovysk) in the southwest of Belarus [[30]](https://www.zotero.org/google-docs/?F85mu5). In the works by Dovzhenok [[33]](https://www.zotero.org/google-docs/?ZV0RSv) and Halubovič [[7]](https://www.zotero.org/google-docs/?w2T9ZH) lentils stand out in their role as the main foods of priests and monks during fasting periods, while peas are also mentioned in this regard.

Dovzhenok [[33]](https://www.zotero.org/google-docs/?mZP0UZ) states that millets were widely grown and even makes the point that millet was one of the main foods of the Ancient Rus’ population. Interestingly, prices of millets in Novgorod were higher than those of rye and even wheat. Halubovič [[7]](https://www.zotero.org/google-docs/?BuJ9k0) repeats that millets occupied a significant number of fields, especially fields that were newly converted into arable land which had the most favourable conditions for this crop. On the other hand, Kiryanova [[29]](https://www.zotero.org/google-docs/?nkbi9b) points to the clear changes in the distribution of millet - while for the second half of the 1^st^ millennium the author refers to it as being common in Ancient Rus, in the beginning of the second millennium it already appears in fewer instances - its percentage within assemblages dropping from 12% to 7.9%, and representation of its grains becoming negligible at the majority of sites.

Kiryanova [[29]](https://www.zotero.org/google-docs/?KOtebm) argues that at the turn of the 1^st^ and the 2^nd^ millennia there are changes in the composition of crops: two new ones appear (rye and oats), which gradually become part of the cultivated grains and more and more firmly take their place among the crops, pushing back the previously widespread wheat, barley and especially millet. Rier [[35]](https://www.zotero.org/google-docs/?SbQeXw) confirms the growing role of rye in the forest zone of Europe from the end of the 1^st^ millennium.

The formation of the GDL does not seem to have brought about any significant changes to subsistence in Belarus based on existing historical and archaeological knowledge. According to Kiryanova [[29]](https://www.zotero.org/google-docs/?7pPQhG), from the second half of the 13^th^ to the 15^th^ century, the tendency of the previous period clearly continues in agriculture, with rye firmly occupying the first place among crops, its percentage in finds only growing and now representing more than half of the whole archaeobotanical material at most sites. Wheat and barley remain popular, but the representation of oats continues to increase. This crop reaches the popularity of the latter two and even rises to the second place in terms of frequency of use by the 15^th^ century - as far as it can be judged from archaeological grain finds at least. As for millet, it continues to decrease in frequency in archaeobotanical assemblages through time [[29]](https://www.zotero.org/google-docs/?jV0cO6).

With regards to the time of the PLC, according to Lakotka [[109]](https://www.zotero.org/google-docs/?BUKppO) the rural economy of the 16-18^th^ centuries was one of the most developed in Eastern Europe. Historical sources of the 16-17^th^ centuries show that largely the same crops were sown in the village as before: rye, wheat, oats, barley, peas, buckwheat, flax and hemp. Their yield does not seem to have increased from the time of Ancient Rus and is quoted as being even lower - 2.5-3 [[10]](https://www.zotero.org/google-docs/?MkAZy9). Korobushkina [[30]](https://www.zotero.org/google-docs/?P57Qrj) simply summarises that the yield on the territory of Belarus remained quite unchanged (2.5-3.5) till the end of the 18^th^ century when it finally grew significantly. According to Bialiavina [[10]](https://www.zotero.org/google-docs/?uOP9pd), millets and small beans were definitely harvested in the 16-17^th^ centuries and occupied an important place in the crop structure of the 19-20^th^ centuries - which somewhat contradicts the conclusion of Kiryanova [[29]](https://www.zotero.org/google-docs/?lAwTwh) regarding the marginal role of millets that diminished over centuries. Additionally, Navahrodski [[31]](https://www.zotero.org/google-docs/?tJK9RA) mentions that in the 19^th^ century millets occupied a small number of fields. As for lentils, based on Bialiavina [[10]](https://www.zotero.org/google-docs/?w65LkQ), they occupied a significant place among crops - however, again there is no numerical information regarding their exact significance.

Based on the work of Navahrodski [[32]](https://www.zotero.org/google-docs/?gnw6SL), agriculture was still the base of the economy of Belarusians in the 19^th^ - first half of the 20^th^ centuries, so we may assume that its leading role and the reliance on cereals as the main food product holds true for the whole period of study - which is supported by the words of Bialiavina [[10]](https://www.zotero.org/google-docs/?J4V0Gh) that until the mid 19^th^ century grain cultures constituted the base of local subsistence in Belarus.

**Gardens**

Garden crops are known from archaeological and written sources from the territory of Ancient Rus, including the territory of Belarus. Overall, the use of garden cultivars became widespread on Belarusian lands in the period of Christianisation and the same crops continued to be grown in subsequent times (excluding the additional ones brought later from the Americas to Europe), including cabbage, onions, garlic, turnips, beetroot, radish, horseradish, carrots, poppies, pumpkins, radishes, and hops (the latter was used for the preparation of different drinks) [[7,34,35]](https://www.zotero.org/google-docs/?EA4k1a). Polack contributes to this list with finds of cucumber seeds of the 13^th^ century. Cucumbers are also found in the archaeobotanical inventory of Viciebsk castle of the 16^th^ century along with rye, wheat, barley, oats, peas, and buckwheat [[35]](https://www.zotero.org/google-docs/?5jQSQz). The most widespread vegetables were cabbage and turnip, while onion and garlic had also long been documented [[33,34]](https://www.zotero.org/google-docs/?e0KPNV). Rakava [[34]](https://www.zotero.org/google-docs/?lPiHyP) supplements this list of garden cultures in feudal times with finds of parsnip, sorrel, rutabaga, beans, and borage. According to Rakava, most of the garden space was allocated for the most important plants: cabbage, turnip, beetroot, rutabaga, onion, and cucumber. These cultivars remained important until the second half of the 19^th^ century with cabbage still being the most dominant, but for this time Rakava also notes that lentil, beans and peas occupied large spaces. Nevertheless the information regarding the quantity of different specific crops grown in gardens varies among researchers, so it was probably different for different regions [[34]](https://www.zotero.org/google-docs/?JhqYjn).

Apart from vegetables and cereals, fruit trees were also grown in gardens, including apples (the most widespread fruit), pears, plums, and cherries [[33]](https://www.zotero.org/google-docs/?Q8wb6g). Plum and cherry pits were found in Polack, Minsk, Hrodna (Grodno), Brest, and Sluck (Slutsk) [[30,35]](https://www.zotero.org/google-docs/?lm7Nyi). Rier [[35]](https://www.zotero.org/google-docs/?yKopTe) confirms that fruits are known in Eastern Europe from Ancient Rus times. From the second half of the 10^th^ century, grapes and, from the middle 13^th^ century, plums are also documented in the Polish lands [[35]](https://www.zotero.org/google-docs/?mjRyIO). Moreover, it is known that fruits were brought to Ancient Rus from foreign lands, especially from Byzantium [[33]](https://www.zotero.org/google-docs/?HQTXll).

Overall, it is believed that gardening played an important role in subsistence and cabbage, beetroot, carrots, onion, turnip, horseradish, and garlic were part of the daily diet [[25]](https://www.zotero.org/google-docs/?6L5sJs). Gardens existed near dwellings even in densely populated areas (e.g. Polack magistrates' registers mention gardens near the town houses) but likely most of them were situated in rural locations and in town suburbs [[9,33]](https://www.zotero.org/google-docs/?PGZ2rS). Keeping a garden for satisfying the needs of the noble family and their servants in terms of vegetables was also typical for the estates of the aristocracy. Basic garden crops were the same - cabbage, turnip, garlic, onion, carrot, parsnip, peas, and beetroot - but they were grown on well-manured soil [[100]](https://www.zotero.org/google-docs/?082uJ3).

With regards to changes occurring in gardening over time, Rakava [[100]](https://www.zotero.org/google-docs/?3NTQCP) suggests potential alterations in crop variety. Based on archaeological research, the variety of garden crops in the 10-13^th^ century was small: cabbage, turnip, onion, beetroot, cucumber, peas, vetch, lentil, and beans. In the 16-18^th^ centuries, written sources show that the same cultures were grown, and also others like radish, poppy, carrot, garlic, parsnip, dill, parsley, pumpkin, horseradish, and caraway [[100]](https://www.zotero.org/google-docs/?1rTneV). Corn started to be grown in Belarus in the 18^th^ century. Grains were used for consumption and stalks for feeding animals [[31]](https://www.zotero.org/google-docs/?XZ19ie). Potatoes, tomatoes and pepper appear later [[31,100]](https://www.zotero.org/google-docs/?fsf0YK), beyond the period of focus of this study.

**Husbandry**

By the start of our period of interest in the 2^nd^ millennium AD, animal husbandry seems to have been a secondary activity compared to crop cultivation [[109]](https://www.zotero.org/google-docs/?YO7Bml). The fact that husbandry was a less important, though still vital, subsistence component, contributing meat and milk into the diet, is confirmed by Dovzhenok [[33]](https://www.zotero.org/google-docs/?uzHC93) and Halubovič [[7]](https://www.zotero.org/google-docs/?zYQQDI). Halubovič [[7]](https://www.zotero.org/google-docs/?yJ8H0a) states that one of the reasons for its limited role were unfavourable conditions for the breeding of animals in large quantities - the territory of Belarus in the beginning of the 2^nd^ millennium was covered with forests providing few pastures. In many cases, woodlands or the floodplains of rivers, free of woodland, were used as pastures. Still, husbandry also provided important surplus products.

Written and archaeological sources show that horse, cattle, sheep, goats, pigs and poultry (geese, chicken, ducks) were commonly kept [[7,25,33]](https://www.zotero.org/google-docs/?FGTFcR). This is confirmed by archaeological finds - e.g. the study by Shcheglova et al. [[28]](https://www.zotero.org/google-docs/?FbmyA3) focused on animal bones from the excavations of several Medieval castles and townships in the north of Belarus, such as Drysviaty, Ikzań, Svir, Maskavičy. Domestic species dominated among the bones - 63% to 93% on different sites - and included large and small ruminants, pig, horse, dog, and cat. Similarly, among 105 animal bones from the Polack region, which were reviewed for sampling in the present research, the identified domestic species included cattle, sheep, goat, horse, pig, rabbit, chicken, goose, dog, and cat. Among the domestic species identified by Shcheglova et al. [[28]](https://www.zotero.org/google-docs/?FPIJ8P) pig was the dominant one, representing 32-42% of the bones; though in one site cattle dominated. According to Gurevich [[41]](https://www.zotero.org/google-docs/?Xn8AZl), the dominance of cattle and pig is typical for the cities of Ancient Rus, where small ruminants played a more marginal role in terms of meat procurement, though their wool was widely used. Ethnographic studies from Belarus are in agreement with the dominance of pig bones - e.g. according to Gurko et al. [[25]](https://www.zotero.org/google-docs/?hPff5v) pork was the most popular among meat products although lamb was more popular in the western portions of Belarus. Likewise, Kaspiarovič [[38]](https://www.zotero.org/google-docs/?SLr9mk) notes that in the 14-17^th^ centuries pigs were widely kept. Often they were kept freely and interbred with wild boar.

Taking into account the proposition that milk products were part of the daily ration [[25]](https://www.zotero.org/google-docs/?tz5KWD), we may assume that cattle and ovicaprids were kept primarily for milk (and as draft animals) and to a lesser degree for meat. Overall, leftovers found in archaeological excavations on the sites reviewed by Shcheglova et al. [[28]](https://www.zotero.org/google-docs/?87s0yW) were represented mostly by adult animals, with few bones having any unfused epiphyses. The fragmentation of the horse bones in all the settlements made the authors suppose occasional consumption of horse meat.

According to Kaspiarovič [[40]](https://www.zotero.org/google-docs/?wXEuVH), in the 19^th^ - beginning of the 20^th^ century husbandry still remained a secondary branch of the subsistence economy. Cows were a source of milk and milk products like butter, cheese, cottage cheese and cattle breeding remained an important economic activity, however. In the 20^th^ century, after the Stolypin agrarian reform and more intensive inclusion of husbandry into the trade and economic relations, the proportion of small ruminants kept by peasants increased relative to cattle. The proportion of the domestic animals kept by peasants at the time was roughly 14% for horses, 24% cattle, 26% pigs, and 36% sheep and goats. By contrast, the nobility kept more horses and cattle and fewer sheep, goats, and pigs. Among poultry, hens were dominant but, in villages near lakes and rivers, geese and ducks were also kept. Apart from hay, animals were also fed with oat straw and vegetables according to the ethnographic data of the 19-20^th^ centuries [[40]](https://www.zotero.org/google-docs/?yqST7m).

**Hunting and gathering**

Hunting, fishing, bortfreat (collection of wild honey), and gathering occupied an important place in the past peasant economy of Belarus, providing additional sources of food. It is evident from mentions in written sources and from finds of fish and wild animal bones in rural settlements, and is supported by ethnographic data [[25,33]](https://www.zotero.org/google-docs/?tSK76b). Among the written sources one may name “Russkaya Pravda”, the legal source from the 11^th^ century, which, among other things, regulated fishing, hunting and bortfreat [[7]](https://www.zotero.org/google-docs/?jV3lVT). At the same time, Shcheglova et al. [[28]](https://www.zotero.org/google-docs/?RIO4gS) describe the role of hunting as marginal at the north Belarusian sites included in their study. This may be in line with the decline of the role of hunting in the 9-13^th^ centuries mentioned by Halubovič [[7]](https://www.zotero.org/google-docs/?XATPiR). At this time, the bones of domestic animals started to outweigh the wild ones in archaeological sites. Still the role of hunting remained significant, and focus shifted from procurement of meat to procurement of fur, traded with the Arabic world, Byzantium, and western Europe. For the feudal aristocracy, hunting was first and foremost an entertaining and social activity, it became one of the favourite activities of the dukes and their druzhyna (armed retinue) [[7]](https://www.zotero.org/google-docs/?GzUqGD).

Wild animals that were hunted included European bison, elk, red deer, roe deer, wild boar, bear, wolf, beaver, fox, hare, marten, lynxes, badgers, otters, sables, ermines, squirrels, minks, ferrets, tarpans, and wild goats, as well as capercaillie, partridges, black grouses, grouses, geese, and ducks [[7,28,33,41]](https://www.zotero.org/google-docs/?8yiV7E). Discussing the prevalence of large animal bones in the city site of Navahrudak of the early 2^nd^ millennium AD, Gurevich [[41]](https://www.zotero.org/google-docs/?bVWf19) proposes that this may be due to the concentration of hunting on meat-bearing animals but also possibly due to the skinning of fur animals in the forest. Among the wild species identified among the animal bones in the present study, there were elk, red deer, beaver, hare, possibly bison and a wild bird.

The gathering of mushrooms, berries, nuts and medicinal herbs, as well as bortfreat, also played a substantial role in the subsistence economy, especially in years of war and famine. The importance of bortfreat and hunting in the times of Ancient Rus was connected not only with subsistence and abundance of forests; they also provided the main export products of the time - honey, wax and fur [[7]](https://www.zotero.org/google-docs/?rwBJcR). As far as archaeological sources are concerned, bortfreat equipment was present in small quantities in the western city site of Navahrudak and is documented in legal documents of the 16^th^ century [[41]](https://www.zotero.org/google-docs/?ciUF2L). Beekeeping starts to be mentioned in written sources from the 13^th^ century [[7]](https://www.zotero.org/google-docs/?AdO1hr), but bortfreat remains a significant trade until the 18-19^th^ centuries [[38,40]](https://www.zotero.org/google-docs/?qGbb30).

**Fishing**

Fishing was a widespread and well-developed occupation, and the numerous lakes and rivers in Belarus provided plenty of fish over the time period of interest. This trade was especially widespread in Palessie and south-western Paazerje [[7,40]](https://www.zotero.org/google-docs/?r7zdYv). The former region is situated in the south of Belarus, but the latter one is located in the north of the country, thus belonging to the focus territory of this study.

Fish bones and fishing equipment are found at archaeological sites throughout the territory of Belarus [[7]](https://www.zotero.org/google-docs/?n7yGJ5). Klimau and Liashkievich [[39]](https://www.zotero.org/google-docs/?a2ySs6) studied fishing in rural settlements in the Polack surroundings in the 11-16^th^ centuries. The territory surrounding Polack, Paazerje, is rich in water resources, including numerous tributaries of the Dzvina and large and small lakes. According to Klimau and Liashkievich, some rural sites in this region, like Lisna-1, Lucna-1 (a rural site with feudal residence) but also Polack itself with neighbouring towns, yielded a substantial fishing inventory - including hooks, net sinkers, spear tips. Hooks are the most widespread equipment. Some of them were suitable for catching large predatory fish. Fishing spears were also found in the 10-11^th^ century burials [[39]](https://www.zotero.org/google-docs/?ak935j). They were used for large fish, such as catfish, salmon and pike [[7]](https://www.zotero.org/google-docs/?GKlXF5). Klimau and Liashkievich [[39]](https://www.zotero.org/google-docs/?HiinBQ) note that the proportion of fishing equipment compared to other finds is rather substantial, up to half of the finds in some squares. The authors point out that the concentration of fishing equipment is higher in lake settlements than in the river ones, but the assortment is similar. Other Medieval settlements in neighbouring regions to Polack have yielded few items related to fishing, however, necessitating further research [[39]](https://www.zotero.org/google-docs/?DbpFpO).

Based on ethnographic and archaeological data, as well as rare mentions in written sources, one can assume that multiple species of fish lived in the 9-13^th^-century lakes and rivers of Belarus, including catfish (the biggest), and the sturgeon (beluga, stellate sturgeon, sterlet, sturgeon) and salmon families (whitefish, vendace, smelt, grayling, salmon) [[7]](https://www.zotero.org/google-docs/?fRlUmX). Western Dzvina yielded very large quantities of salmon in the 15-16^th^ centuries according to Halubovič [[7]](https://www.zotero.org/google-docs/?jwOD0Q). Kaspiarovič [[38]](https://www.zotero.org/google-docs/?mezTO7) agrees that beluga, sterlet, and sturgeon appeared in Belarusian waters but only rarely. Other species caught included sea and river lamprey, pike, trout, carp, taimen, burbot, flounder, eel, pike perch (zander), perch, tench, bream, ide, chub, loach, guster, bleak, and rudd [[7,41]](https://www.zotero.org/google-docs/?TSAwrp). One of the most widespread and valuable fishes in the 9-13^th^ centuries was the vimba [[7]](https://www.zotero.org/google-docs/?6xvnTp).

A collection of fish remains was gathered at Lisna-1 in the Polack region. Most other sites mentioned by Klimau and Liashkievich [[39]](https://www.zotero.org/google-docs/?ifQAKN) have poor conditions for the preservation of fish remains. Eight fish species were identified at Lisna-1. Most of the remains came from the 11-13^th^ centuries and a few from the 15-16^th^ centuries. The majority of the remains belong to perch, which was likely most hunted in winter, specifically in January-March. Members of the carp family occupy second place in terms of importance, among which the order of popularity is from bream being most popular, followed by tench, roach and ide. Pike perch remains were substantial but mostly connected to the later period of the settlement’s existence. Pike and catfish were present in small quantities. The presence of ide, tench and catfish indicates summer fishing. All of the mentioned fish are lake-river types [[39]](https://www.zotero.org/google-docs/?jYwZdJ). For comparison, among the fish remains found in Vilnius’ lower castle dating to the 14-17^th^ centuries, which reportedly represents the fish consumption of the highest social classes, Piličiauskienė and Blaževičius [[110]](https://www.zotero.org/google-docs/?jDeuUS) name pike as the most frequently consumed species, followed by different cyprinids (bream, tench, ide, common carp, vimba and asp) and pike perch. Bones of sturgeon, wels catfish, perch and salmon were also present, but no remains of marine fish were found, including a lack of herring (probably due to hand collection of the bones during excavation) and cod.

Fishing brought additional food products and profit for the populations of Belarus, with fish being a common item at markets. Supposedly fishing with a fishing rod in the 9-13^th^ centuries acquired elements of entertainment for common people in addition to being a subsistence activity, while the feudal aristocracy preferred hunting with dogs and falcons. Fisher unions started to appear in the 12-13^th^ centuries. They were formed around the church as with the spread of Christianity priests wanted the inhabitants to follow fasts [[7]](https://www.zotero.org/google-docs/?avQWPr). It is also known that peasants had to catch fish for landlords as one of their tributes [[38]](https://www.zotero.org/google-docs/?DLG8J2).

**Use of manure**

The 2- and 3-field crop rotation farming system was dominant in Ancient Rus, including the Belarusian territory, in the 10-13^th^ centuries. The wide use of this system of agriculture from the 9-11^th^ centuries was supported by the development of husbandry, which provided manure. Older slash-and-burn agriculture was not systematically applied any more, though remained in use in some areas, especially in southern regions, till the 19-20^th^ centuries [[7,10,30,33]](https://www.zotero.org/google-docs/?mWJwzs). Rier [[35]](https://www.zotero.org/google-docs/?7TLGM1) confirms the dominance of the three-field rotation system on east Slavic lands for the 14-15^th^ centuries. This system was legalised on Belarusian lands in the mid 16^th^ century by the so-called Voloka Decree and was still in use in the mid 19^th^ century [[10]](https://www.zotero.org/google-docs/?4jgt0R).

Manuring is a necessary component of the crop rotation system, and on the territory of Belarus in the 10-13^th^ centuries dung was used as manure [[30]](https://www.zotero.org/google-docs/?qdei3A). Dovzhenok [[33]](https://www.zotero.org/google-docs/?5W5CJX) and Halubovič [[7]](https://www.zotero.org/google-docs/?OjcIBE) agree that though fertilization of fields in Ancient Rus is not mentioned in written sources till the 15^th^ century, the benefits of dung and ash for field fertility were undoubtedly well-known. However, according to Guagnini (cited in Koledinskiy [[37]](https://www.zotero.org/google-docs/?TYJk4I)), manuring was not used in northern Belarusian territories in the 16-17^th^ centuries, while Koledinskiy [[37]](https://www.zotero.org/google-docs/?IJQVp2) confirms the same archaeologically for the 12-16^th^ centuries’ cultural layers in Viciebsk. Writing about the unchanging crop yield throughout the centuries of study, Bialiavina [[10]](https://www.zotero.org/google-docs/?vt9v3C) proposes that it was insufficient manuring and unaltering tools that kept the fertility low. Agricultural work in spring started with bringing manure to the fields. However, limited pastures allowed a limited number of animals to be kept, so not all fields were equally manured [[10]](https://www.zotero.org/google-docs/?PIcnJ9). As this is an ethnographic observation, it can only be directly applied to the 19-20^th^ centuries. Nevertheless, the observations by Koledinskiy [[37]](https://www.zotero.org/google-docs/?2aiE1r) make it probable that the use of manure was indeed limited in the territory of our study throughout the 2^nd^ millennium AD. At the same time it is worth mentioning that speaking about the gardens of nobility estates, Rakava [[100]](https://www.zotero.org/google-docs/?JnYufH) mentions them being grown on well-manured soil. Zaharuĺski [[111]](https://www.zotero.org/google-docs/?A04FrY) states that while rural settlements of the 11-13^th^ centuries in Western Rus might lack finds of manure due to the poor preservation of the cultural layers, concentrations of manure are found in the well-studied town sites (Polack, Minsk, Brest, Pinsk), where cultural layers preserved organic material. They are especially common in barns, leading to a suggestion that town dwellers did not manure fields, even though they definitely practiced agriculture. At the same time, in the later layers of the 16-17^th^ centuries, such concentrations are still found, even though manuring of the fields is already traceable through written sources. Overall, Zaharuĺski [[111]](https://www.zotero.org/google-docs/?gG5Xar) concludes that, based on archaeological sources, it is only possible to say that conditions for the maintaining of the soil fertility through manuring existed, though its exact extent and intensity remains largely unknown at present.

**Diet in urban and rural settings**

Peasants at the time of Ancient Rus were divided into free members of the rural community, who only had to pay rent to the state in the face of the duke, and feudally dependent peasants, who lacked economic freedom and depended on the requirements of their feudal lord. Most of the rent was taken in the form of subsistence products. Corvee also existed, as well as monetised rent. Meanwhile, the feudal castle or estate would act as economic, administrative and military centres [[7]](https://www.zotero.org/google-docs/?KuEb5Z). Peasants were still the most numerous social class in the GDL, and the basis of their economy was agriculture. The dependency of peasants grew over time and, in the second half of the 16^th^ century, significant social changes came about in the form of an agricultural reform (Voloka Decree of 1557), codifying the feudal ownership of the land and the dependency of peasants. Demand for agricultural products from the west led to the growing orientation of estates towards their production for sale. This caused landowners to change feudal rent to corvee (obligation of tenant farmers to work a certain number of days on the landlords’ estates), which contributed to the loss of land and freedom by the peasants. The legal position of peasants worsened in the 17-18^th^ centuries due to the increase of corvee demands. Additionally, wars with Russia and among the local feudal lords destroyed the crops of peasants [[34,100]](https://www.zotero.org/google-docs/?bYGE8X).

In the 9-13^th^ centuries, cities grew as centres of craft and trade on the territory of Belarus. According to written sources, 38 cities existed on the territory of Belarus at this time. The main occupation of the inhabitants of these cities was trade and crafts, while part of them were involved in agriculture. Apart from craftsmen and merchants, dukes, nobility and clergy lived in the cities [[22]](https://www.zotero.org/google-docs/?YRgpVE). Polack, the city of interest in this study, was the main craft and trade centre of Ancient Belarus and had several markets [[7]](https://www.zotero.org/google-docs/?3DFSl9). Duk [[9]](https://www.zotero.org/google-docs/?H5LChZ) characterises Polack as a craft and trade centre of the region. Craftsmanship is supported archaeologically for the period spanning the 10-18^th^ centuries. Written sources add to this picture in the 16-18^th^ centuries, when the principle that only those who were engaged in craft or trade activity could be considered fully-fledged urban citizens was lawfully enforced [[9]](https://www.zotero.org/google-docs/?Iivwas).

In contrast to cities, villages continued to rely on agricultural economies in the 9-13^th^ centuries [[7,29]](https://www.zotero.org/google-docs/?IfX5Yp). Urban inhabitants were the main consumers of village production. On the one hand, agricultural activity was common in cities in the 10-16^th^ centuries and city inhabitants also kept gardens and domestic animals [[7,9,29,30,33,37,41]](https://www.zotero.org/google-docs/?rmtb9B). For examples, Duk [[9]](https://www.zotero.org/google-docs/?IaJS8u) points out to the existence of agricultural suburbs of Polack, known from the written sources of the 14-18^th^ centuries and 17-18^th^ centuries’ folwarks across the river Palata. Documents show that Polack’s inhabitants practiced agriculture and owned large pieces of land, not only in far away places beyond Palata and along it, but also gardens directly near to the city’s fortifications. The agricultural inventory (scythes, forgings from shovels, hoes) found in Polack testifies to the close contacts of the city with the village in the 16-18^th^ centuries. Moreover, cases are known in the first half of the 16^th^ century when peasants acquired grain in Polack [[9]](https://www.zotero.org/google-docs/?6SbCp2). However, the agricultural and husbandry activity in the city was not enough to fully satisfy the needs of inhabitants and cities obtained much of their food from the rural surroundings [[7,29,30,33,37,41]](https://www.zotero.org/google-docs/?tTI0eM).

The development of crafts and trade led to a growing demand for food products in the cities - according to written sources like the “Russkaya Pravda”, one could buy meat, honey, salt, wheat, rye, millet, oats, hops, vegetables, fruits, fish, milk, flour, baked bread, cheese, butter and other products at the city markets. Salt and rye had the biggest turnover [[7]](https://www.zotero.org/google-docs/?OrAxL8). Lakotka et al. [[22]](https://www.zotero.org/google-docs/?yGcsy2) mention beer and mead being consumed in city taverns in the 15-16^th^ centuries, with vodka taverns starting to outnumber beer and mead establishments in the 17^th^ century. Writing about this later period, Holubieŭ [[43]](https://www.zotero.org/google-docs/?vAv934) mentions the hypothesis of eastern Belarusian cities remaining the main consumers of products from the village, with the majority of grain being exported from the western parts. Polack was still a crafts and trade centre at the time of the GDL. Written sources of 1500 mention bakers, butchers, brewers among other craftspeople [[9]](https://www.zotero.org/google-docs/?yReDHb), giving already some information about the possible food of the urban dweller. Moreover, the butchers’ corporation was the most prominent and influential among craftspeople in the first half of the 16^th^ century [[9]](https://www.zotero.org/google-docs/?KtZMAv).

Given that city dwellers cultivated the same crops as rural inhabitants and used the same agricultural techniques [[30]](https://www.zotero.org/google-docs/?X8igtx), and also obtained much of their food products from the rural neighbourhood, we may assume that the diet of urban commoners was similar to that of the rural commoners, at least in the beginning of the period of study. Information related to import may enrich our understanding of possible differences between urban and rural diets. For the 10-13^th^ centuries, amphorae occupied the second place among all items of import found in archaeological excavations. Their contents were primarily wine from the Black Sea provinces of Byzantium. The only other imported foodstuff among finds in Polack would be walnut shells [[9]](https://www.zotero.org/google-docs/?sXdSyU). Thus, one possible difference between the diets of peasants and citizens could be that foreign goods, including food products, almost never reached the village [[7]](https://www.zotero.org/google-docs/?YaH7TY). However, export in the region dominated over import throughout the Medieval period [[112]](https://www.zotero.org/google-docs/?DbbrC7). Already from the establishment of the city until at least the 16^th^ century AD grain is mentioned as one of the main exports from the Belarusian territories, including the Polack neighbourhood, among other agricultural and forestry products [[7,33,45,113]](https://www.zotero.org/google-docs/?oJx2ZC). Movement of foodstuffs in the opposite direction seems to be more focused on luxurious rather than basic foods. For example, in the 9-11^th^ centuries foreign trade focused on the Arabic Caliphate and Byzantium [[7,13]](https://www.zotero.org/google-docs/?1KWzQS). Imports from Byzantium were regular in the 12-13^th^ centuries until the occupation of southern Rus lands by Mongols, and the imported food items included wine, oil, and walnuts [[9,44]](https://www.zotero.org/google-docs/?JboCo9)*.* In the 12-13^th^ centuries, trade with Western Europe started to play a major role, while Kievan Rus cities, as well as the Baltic region, remained important partners. In the early 13^th^ century, the Hanseatic League agreements between Riga and Gotland on the one side and cities of Smolensk, Polack and Viciebsk on the other are documented, with the trading post in Polack existing from the first half of the 14^th^ century till the beginning of the 16^th^ century. Agricultural and forestry products constituted the main exports from Eastern European lands, while the food imports to Polack did not change much over the time of Hanseatic trade and included salt as the main import, as well as salted herring, beer and wine, spices and fruits like cherries, apples, pears, plums, even southern figs and grapes [[7,9,13,45,46]](https://www.zotero.org/google-docs/?LfaQH7).

The volume of trade, both local and international, grew in the 15-16^th^ centuries, with grain remaining the leader in terms of food exports, and salt as the main import product, accompanied by wine, spices, and herring [[114,115]](https://www.zotero.org/google-docs/?qhLOao). From the 16^th^ till the 18^th^ century, the role of Viciebsk as the trade centre in the north of Belarus grew [[112]](https://www.zotero.org/google-docs/?YCwolU). To sum up, in terms of long-distance trade, staple foods like grain were only exported by the Polack region, while imports of food focused on luxury products like wine, spices, fruits, as well as salt that are perhaps less likely to have made major contributions to the diet. This situation suggests that, if differences based on access to exotic foreign foods existed between rural and urban communities, they were rather marginal and more applicable to rich citizens than commoners.

**Diet of the elites**

As mentioned above, peasants were the most numerous members of Belarusian society under the period of study, though the percentage of nobility at different times reached up to 10-12% of the population [[100]](https://www.zotero.org/google-docs/?WZ161v). It is likely that the wealthier citizens and nobility of Polack had greater access to imported foods like wine, spices, and fruits. Another notable city of the time is Navahrudak, one of the most prominent cities of south-western Ancient Rus, and the probable first capital of Lithuania. Feasts were an integral part of life for the inhabitants of the rich district of Navahrudak, and hardly any excavated building of the 12-13^th^ century was without fragments of amphorae from the Black Sea region. The tableware of various kinds is also dominated by glasses and cups for the consumption of wine. In the rich houses of Navahrudak, there are no agricultural tools used in the 10-11^th^ centuries, but there is much more hunting equipment. Usually a rare find in Ancient Rus’ settlements, harpoons are frequently found among this equipment [[47]](https://www.zotero.org/google-docs/?FGC0iB). Hunting was likely not just undertaken to meet economic needs, but was also a source of entertainment for the rich inhabitants of vakolny horad (the fortified part of the city adjacent to its centre) [[41]](https://www.zotero.org/google-docs/?e2gkrr). According to Gurevich [[47]](https://www.zotero.org/google-docs/?U52sA2), hunting played an exceptional role in the cities of Black Rus, and in Navahrudak specifically - 36-58% of animal bones found there belonged to wild species. This significant role of hunting is not necessarily true for other early cities of the Belarusian lands, however.

The evidence of feasting and hunting may suggest that the nutrition of elites in Ancient Rus was higher overall and richer in meat in particular. At the same time, Navahrodski [[48]](https://www.zotero.org/google-docs/?CNh3Tk) proposes that until the 16^th^ century social elites consumed almost the same products as commoners, maybe only of better quality and with greater regularity. A nobleman often ate at the same table with servants and during military campaigns ate the typical food for warriors - oatmeal - alongside everyone else. With the growth of commodity-money relations and the influence of Poland and Western Europe, the nobility and wealthier citizens started to use more and more imported products. From the 16^th^ century, the cuisine of nobility was influenced by Poland, for which consumption of products with excessive fat content, the presence of sweet dishes, and use of a lot of spices, testified the high status of the host [[48]](https://www.zotero.org/google-docs/?KCVhKx). Lakotka et al. [[22]](https://www.zotero.org/google-docs/?roKrCS) likewise note that feasts after hunting, with consumption of a substantial amount of food and wine, were typical for the nobility of the region. They note that Hungary sold all of its wine within the PLC, illustrating the statement that the ability to drink a lot was a respected skill here.

Apart from the mentioned Polish influence, the overall culinary variety grew with time. Multiple ethnic groups appeared among the population of Belarus as a result of trade and political connections: Poles (14^th^ - beginning of the 20^th^ century AD), Jews (15-18^th^ centuries AD), Tatars (14-17^th^ centuries AD), Russian Old Believers (17-18^th^ centuries AD), Romani (15-20^th^ centuries AD), Germans (16-20^th^ centuries AD) and others. Their cuisines had their own peculiarities, as well as those of different confessions [[25]](https://www.zotero.org/google-docs/?QKnFAy). Overall, historical and archaeological evidence indicates that dietary variability and access to non-local resources increased throughout Europe during the Medieval and Early Modern periods, with Belarus being no different [[5]](https://www.zotero.org/google-docs/?1EIs4k).

Navahrodski [[24,48]](https://www.zotero.org/google-docs/?n4K7rF) and Skiepjan [[23]](https://www.zotero.org/google-docs/?ZwKDyq) have studied information regarding the food and cuisine of the GDL and PLC elites. According to Navahrodski, as with peasants, bread was the main flour-based product consumed by the elites and was a respected symbol of wealth. Social elites consumed bread made of flour of a higher quality, and there was a royal type of bread made based on milk. Different kinds of elaborate bread were made for holidays. Sugar, chocolate, vanilla, almonds and other nuts, saffron, lemon and fruits could be used in its preparation. Rusks were made during fasting periods [[48]](https://www.zotero.org/google-docs/?n6SAB3). Bakeries in the kitchen spaces contained several ovens for bread, which highlights its important role in the daily diet of people at the time [[23]](https://www.zotero.org/google-docs/?13PlyO).

Different groats (or porridges) were also made from grain. Written sources mention buckwheat, barley, wheat, rye, and oat-based products. Among vegetables, turnip and radish, cabbage, carrots, beetroot, cucumbers, onions, garlic, peas, beans, tomatoes were also widespread. Many vegetables, including cucumbers, beans, asparagus, peas, sorrel, dill and parsley, cabbage, beetroot, were preserved for winter in noble houses through salting, pickling, and marinating. Poppy was widely used, especially in baking festive dishes [[48]](https://www.zotero.org/google-docs/?YtdxVJ). Fruits were more widely used by the nobility than by peasants as the former kept fruit gardens [[25]](https://www.zotero.org/google-docs/?IDKFlW). Wide usage of different kinds of mushrooms was also typical for noble cuisine - mostly dried, but also fried, boiled, marinated, salted, added to other dishes and eaten on their own [[48]](https://www.zotero.org/google-docs/?pOYgVY).

As with peasants, husbandry was a significant branch of the economy for the elites but remained secondary to agriculture. Cattle played a particularly significant role in elite husbandry. Rich households also bred horses, animals that were valued a lot and used in agriculture and for transport. Pigs, sheep and goats were also kept by nobility, though goat breeding was less developed. Meanwhile, chicken, ducks, geese, and turkey appeared amongst the poultry [[38]](https://www.zotero.org/google-docs/?oupVvQ). Meat and meat dishes occupied a significant place in the diet of elites. Pork was most often consumed, but also the meat of sheep, cattle and poultry. A significant part of the meat consumed was acquired in the course of numerous hunting campaigns. For preservation, meat was salted or smoked. A lot of fat and meat of pigs was stored in noble households and often pig products were sent to markets for sale. Pork, as well as game and poultry sausages were a typical food stored by the nobility [[38,48]](https://www.zotero.org/google-docs/?hcuQj6). Milk products, especially butter and cheese, occupied a significant place in the diet of the elites as well, including sweet cheeses [[48]](https://www.zotero.org/google-docs/?ysSEqX).

Hunting was the favourite entertainment of the nobility, including those who lived in cities. As mentioned above, hunting would be followed by feasts with a large amount of food and drink (e.g. wine). Hunted animals included deer, elk, wild boar, wild goat, doe, pheasant, grouse, partridge, and hare [[22,48]](https://www.zotero.org/google-docs/?8qLs6m). Fish was a significant product in the diet of the Belarusian elites and was especially actively consumed during numerous fasts [[48]](https://www.zotero.org/google-docs/?I5my7X). Some estates on Belarusian territory also practiced fish breeding, e.g. of trout and carp. Fish was consumed fresh (boiled, fried) and preserved (dried, smoked, salted). A lot of herring was consumed as well as crayfish and eel [[48]](https://www.zotero.org/google-docs/?Wu1mBT). This is true for other parts of the PLC as well - e.g. as mentioned in the written sources, the Master of the Teutonic Order used to send herring to Grand Duke Vytautas during the fast [[110]](https://www.zotero.org/google-docs/?mK9r08). Likewise, herring was among the most frequently mentioned fish in the 14–15^th^-century written records of Poland, and it was very popular in the early 12^th^ century in Poland [[98]](https://www.zotero.org/google-docs/?jDlman). In the territory of the GDL, according to historical data, the Duke’s Court and all elites favoured freshwater fish, mostly pike, with common carp being second in popularity. Zooarchaeological data from Vilnius’ Lower Castle support some of these observations, though the lack of marine fish bones found there seems surprising, given that marine fish in Poland was found in cities at a similar distance from the sea as Vilnius. Moreover, the Palace of the Grand Dukes would get foodstuffs from more distant countries, such as oysters, figs, capers, almonds, etc. [[110]](https://www.zotero.org/google-docs/?33QNnl).

Drinks consumed by Medieval Belarusian elites included mead, beer, home wine, liqueurs and vodka. Infused vodka became popular among the rich in the 18-19^th^ centuries. The host of the feast was satisfied if none of the guests remained sober [[48]](https://www.zotero.org/google-docs/?zPgjcD).

From century to century the menu which was served for the aristocrats’ tables became more complicated: novel products appeared next to the traditional meat and fish dishes. At the beginning of the 16^th^ century these were citrus fruits (oranges and lemons), different types of lettuce; and later potatoes, asparagus, artichokes and even snails were added. Additionally, in the 16^th^ century there was some differentiation among cooks in terms of their specialization in local cuisine, including Moskovian and Hungarian dishes. In the 17^th^ century, French cuisine gained growing popularity and narrow specializations started to appear among cooks like pate-maker, baker, and confectioner. Servants responsible not only for food but also for drinks existed - including wine, vodka, mead, beer. Wine was bought in large cities from merchants or from the countries where it was made - e.g. servants could be sent to Hungary. Wine went exclusively to the lord’s table, while beer was made in the estate itself and formed part of both the lord’s and the servant’s regular diet. There were multiple kinds of beer - wheat beer went again only to the lord’s table, and for others barley beer dominated [[23]](https://www.zotero.org/google-docs/?hS1xoR).

Thus, the cuisine of the nobility, especially in the second half of the 2^nd^ millennium, depended a lot on imported products - in contrast to the cuisine of commoners, particularly those living in rural areas. Some noblemen were used to eating sweets, rice and corn dishes, macaroni from Italy, tea and rum from England, herring from Riga, and foreign fish and caviar. Despite foreign influences, the noble cuisine of the 18^th^ century had its distinctive features, including (1) excessive fat content, a lot of meat and butter, (2) a lot of sweets, dishes based on sugar and honey; and (3) a lot of spices and salt - signs of richness. Whole boars stuffed with sausages, turkey and partridges or whole pikes could appear on the table at the time. Soup was the first course; second was a large amount of different kinds of meat - beef, lamb, turkey, geese and other poultry. During fasts fish was consumed instead and more exotic dishes could appear on the table, e.g. beaver tails, snails, frogs, turtles, chicken scallops. Pies, cakes, sweets and fresh fruits were eaten for dessert [[48]](https://www.zotero.org/google-docs/?zOSpUD).

To conclude, the diet of elites seems to have been more abundant in general and much richer in meat protein in particular, as well as more versatile and even dependent on imported exotic foods, when compared to that of commoners. Its variability grew over time, and there are suggestions that the gap between common and elite diets increased as well, particularly so after the 16^th^ century, when abundance in nutrition is described as having reached gluttony for the richer part of the nobility.

**S3 Note. Further details about sites and contexts analysed in the text**

**Number of graves and individuals**

Table 1 in the main text provides the number of inhumated individuals excavated in each site, which was defined as follows:

- Cremated individuals found at the same kurgan cemeteries were not included in the count, as their number was not always identified and provided in the literature;
- The number of individuals does not match the number of excavated graves, as in some cases several individuals were buried in the same grave and, in other cases, the kurgan/grave was empty (skeleton not preserved or never present). In some cases the reported number differs between several publications: for example, for Polack Lower Castle some authors include the individuals recovered from the spoil heap at the end of the excavation and questionably dated to the 13-14^th^ centuries in the count and others, including the authors of this paper, do not;
- The overall number of graves found was not reported, as it is not provided in the literature and arguably hard to determine for pit burials. For kurgan burials their number in the cemetery was often reported in the literature but variable depending on the time when it was counted. For example, for Biruli, Vajtsjakhovich [[55]](https://www.zotero.org/google-docs/?Ghx9ul) counts 115 mounds but mentions that in 1971 130 mounds were observed there; in Domžarycy 180 are reported by Štýchaŭ [[62]](https://www.zotero.org/google-docs/?qIqMAn) with previous number being 209; the number of kurgans in Sielišča decreased from 78 to 52, while local inhabitants report it was once close to 200 [[76]](https://www.zotero.org/google-docs/?W0ssYF) and a similar situation is observed in Skrabianiec [[73]](https://www.zotero.org/google-docs/?3ONgac). This situation can be explained by the gradual destruction of the kurgan mounds, particularly during agricultural activity;
- The actual number of individuals excavated in some graveyards (e.g. Polack Township, Biruli, Domžarycy, Ziabki, Sielišča) is higher, as the authors of the available publications or reports mention the history of earlier excavations (mostly at the end of the 19^th^ century or in the 1930s and 80s) without details (number of inhumations, adults, overall individuals found in graves), thus they are not included in the individual count. Likewise, reports or publications for each year of excavation were not always available and the number of kurgans versus the number of burials, as well as the number of specifically adult individuals in these burials, was not always made clear in those reports that were available. In some cases the estimate of excavated individuals relied on the compilation of information from several reports and conversations with archaeologists.

**Burial custom: kurgan (mound) and ground (earth/pit burials)**

The human remains analysed in this study originate from burial sites that include kurgan (burial mound) and ground (pit burial) cemeteries in rural and urban environments. Archaeologists observe the evolution of burial rite from pagan to Christian during almost five centuries in the kurgans of Polack lands. The burial rite changes from cremations with sacrifice of animals in long and round mounds (5-10^th^ centuries) to inhumation at the horizon level, often with remains of fire (11 - first half of the 12^th^ century). At the beginning of the 11^th^ century burials in pits under mounds appeared and became widespread in the 12-13^th^ centuries (see S1 Fig for an example of a kurgan burial with a pit under the mound). Some of the common finds in the 11-12^th^ century Polack kurgan burials include female ornaments in the form of temporal rings, numerous beads from neck ornaments (and in richer burials neckrings), zoomorphic bracelets. Male burials in most cases have little inventory, most of it being tools, belt accessories (knives, parts of the belt); ceramic pots are found both in male and female burials [[62]](https://www.zotero.org/google-docs/?CYE8ps).

Early ground cemeteries partly contemporaneous to kurgans are characterised by stone constructions in the form of a pavement or lining, often with larger stones at the head and/or foot of the burial (S2 Fig). The stone installed at the head could be incised with a cross [[52–54]](https://www.zotero.org/google-docs/?CtzUtG). Such ‘stone graves’ are mostly dated to the 13-14^th^ centuries AD, though they existed until the 18^th^ century [[53]](https://www.zotero.org/google-docs/?5QZvLO), and are seen as a transition from the kurgan burials to modern earth burials [[62]](https://www.zotero.org/google-docs/?NgzxsF). Kurgans and flat graves are frequently combined in long-used burial complexes throughout the region. A large concentration of such burial complexes is found in the south of the Belarusian Dzvina (also Dvina, Western Dvina, or Daugava) basin - the territory of our study. Compared to the burial rite of the previous period, which often included multiple grave goods, in the 14-18^th^ centuries AD the deceased were interred without any grave goods [[52,54]](https://www.zotero.org/google-docs/?p2tb6L).

The chronology of the sites is therefore established through a combination of burial rite and grave objects and is usually more narrowly defined for the earlier period where objects were typically present in kurgans (range within up to three centuries), but is established rather broadly for the later pit burials that typically lack grave goods. For example, kurgan cemeteries of Biruli, Domžarycy, Dzmitroŭščyna, Padsvillie, Sielišča, Skrabianiec, Pieravoz-4 are dated by the finds of inventory typical for the kurgans of the 11-12^th^ centuries (pots, headdress and temporal rings’ remains, beads, neckrings, zoomorphic bracelets, knives); in Kazloŭcy a similar set of objects and dirhams of the 10^th^ century were also found. The dating of Ziabki and Vaŭča kurgans was also established based on the finds of objects (ring, pedant, button in the former and plaques of a headdress in the latter) combined with cemetery type.

For the later ground burials, mass ceramic finds deposited over centuries of the cemetery’s usage and interpreted as commemorative inventory (coupled with the few grave objects including several coins) were the main basis for establishing the wide chronology - this is the case of Ivieś, Michalinava, Klieščyno, Dubraŭka, Doŭhaje. Ceramics and object typology were also the basis for dating the ground cemeteries adjacent to kurgan graveyards in Biruli (earrings of the 14-16^th^ and a coin of the 16^th^ century), Vaŭča (ring, plaques of headdresses), Pieravoz-4 (headdress plaques).

The dating for the ground cemetery of Polack Lower Castle was established on the stratigraphy and the few archaeological finds relevant to each burial group (ring, coffin nails, bullet). Other cemeteries excavated within the city of Polack were also dated based on objects and ceramics - ceramic fragments and pots, cross pendants, lead bullet in the Township; spindles and fragments of glass bracelets in the Upper Castle.

As animal bone assemblages were typically not reported in publications, their dating and provenance had to be obtained directly from excavators and their field notes, and was based on the archaeological context - e.g. the layer, feature or pit and artefacts found in it.

Unfortunately, only in the case of Polack can the described cemeteries be directly related to the settlement, while in case of the other sites no such data is available in publications. They are therefore defined as rural based on the lack of any known urban settlements in their vicinity, contrary to sites like Polack where the city still exists or is known to have previously existed based on archival or archaeological data (for example the ground cemetery near the village of Pašavičy, which is referred to as urban due to the archaeologically-documented early Medieval city nearby [[70]](https://www.zotero.org/google-docs/?oFR0OU)). Therefore, we describe only the rural cemetery sites without reference to rural settlements below.

**Rural sites**

**Biruli** is an example of a burial complex with a prolonged history of interment. First, a kurgan cemetery with cremation and inhumation burials was created in the 10^th^ century by a culture of Polack-Smolensk Long Barrows. It contained both elongated kurgans (earlier) and round ones. At the end of the 10^th^ and the beginning of the 11^th^ century the burial rite changed to inhumation, probably in connection with the arrival of Slavic populations to the territory occupied by Polack-Smolensk Long Barrows culture. Burials with this rite are dated to the 11-12^th^ centuries. Almost all of the mounds contain stacks of stones or a single stone. In most cases the individuals were buried in a pit under the kurgan, in a few cases they were found in the virgin soil level over ritual fire [[55]](https://www.zotero.org/google-docs/?vdSSTu). The same cemetery complex near Biruli village includes ground burials marked by stone structures, some of which were excavated and dated to the 14^th^ - beginning of the 16^th^ century (S3 Fig). Initially, the area among the kurgans was used for interment then, due to lack of space, burials began to penetrate mounds [[56]](https://www.zotero.org/google-docs/?eJenJ5).

The burials of the kurgan cemetery named after the village **Domžarycy** are mostly dated to the 11^th^ century. The cemetery was located in the centre of Polack Principality during the time of its prosperity [[62]](https://www.zotero.org/google-docs/?7ekPir). The kurgans are attributed to the East Slavic tribal union of Kryvičy, though Štýchaŭ [[62]](https://www.zotero.org/google-docs/?dTyF2e) also mentions some particularities of the kurgan group - for example, inhumations with molded ceramics similar to that of the Long Barrow culture of northern Belarus, which practiced cremation. Folding of the hands of the deceased under the chin on the chest, which is characteristic for Latgals, also appears in this cemetery more often than in others in the region [[62]](https://www.zotero.org/google-docs/?QzrYy2).

The cemetery near the village of **Doŭhaje** includes three parts: kurgan, abandoned ground cemetery and active cemetery. Based on the funeral rite and equipment, the kurgan burials date back to the 11^th^-12^th^ centuries [[77]](https://www.zotero.org/google-docs/?WOIE9Z). Some mounds in this cemetery appear among ground burials; possibly the latter tradition replaced the mound burials by the 13^th^-14^th^ centuries, but this hypothesis requires additional research. In the older part of the ground cemetery, many graves are covered with stones, with larger ones at the head and foot of the burials. There are also stone crosses and boulders with incised crosses and other signs [[63]](https://www.zotero.org/google-docs/?TQJ7oK). Charauko [[63]](https://www.zotero.org/google-docs/?LV08sl) concluded that the excavated part of the cemetery with ground burials functioned in the 14-18^th^ centuries, owing to the finds of ceramics from the 14-16^th^ centuries and a coin from 1743 - all found in the upper stratigraphic layer and interpreted as a commemorative inventory. As most of the burials are believed to belong to the earlier part of this broad dating period, the 14-16^th^ centuries, this site is considered within the group of the 13-16^th^ centuries in the present work. The kurgan cemetery named after the village of **Ziabki** is adjacent to the Doŭhaje ground cemetery and probably constitutes part of the same long-functioning burial complex. Given that small parts of the graveyards have been excavated so far, it is not yet possible to assert that there is continuity between barrows and ground graves. In addition, no settlements to which either type of the burials could belong have been identified yet. The Ziabki kurgans are dated to the 11-12^th^ centuries and are considered to belong to Kryvičy [[77]](https://www.zotero.org/google-docs/?ZwacMT).

Similarly to Doŭhaje, the cemetery near the village of **Dubraŭka** includes an active graveyard, an abandoned ground cemetery with stone crosses, and kurgans. The abandoned ground cemetery was excavated and contained burials at two horizons, suggesting use of an existing burial ground for new interments [[65]](https://www.zotero.org/google-docs/?gpDSZz). Earlier burials are well-connected with stone constructions, which were possibly dismantled during new burials and then reconstructed in their original form. A large number of the graves contain children sub-burials - children buried in existing graves of adult individuals. This tradition is also found in similar cemeteries in the region (e.g. Klieščyno), thus they can be considered part of a local burial custom. The excavated part of the cemetery functioned between the 17-18^th^ centuries [[65]](https://www.zotero.org/google-docs/?vtSO0Z).

The kurgan cemetery named after the village of **Dzmitraŭščyna** is located 10-15 km to the north of Polack. It is dated to the 10^th^ - beginning of the 12^th^ centuries and is considered to have been constructed and used by a mixed Balto-Slavic population [[66]](https://www.zotero.org/google-docs/?X5Uop5).

The ground cemetery site near the village of **Ivieś** functioned in the 14-18^th^ centuries, with most burials dated to the 14-16^th^ centuries [[54]](https://www.zotero.org/google-docs/?GYlbco). A kurgan group is located nearby, which is yet to be studied extensively. Graves are marked with stone constructions, including stone covers or contours and larger stones at the head and foot. Some boulders have incised crosses [[67–69]](https://www.zotero.org/google-docs/?dsXovY).

The ground cemetery with cremations and inhumations near the village of **Kazloŭcy** is dated to the 10-12^th^ centuries. Artefacts reflect a mixed Balto-Slavic population. A large number of ash stains and oval pits with charcoal were found in the cemetery, apparently having ritual uses. One of the pits contained a skull and other bones of a horse. Female graves excavated at the cemetery contained many grave goods, mostly ornaments but also knives and pots. Two male burials yielded only one knife by comparison [[70]](https://www.zotero.org/google-docs/?TrNej1). The early dating and presence of grave goods stand out as unusual for a typical ground cemetery in the region, but Plavinski [[116]](https://www.zotero.org/google-docs/?KHDH28) believes it was actually a kurgan cemetery.

In the ground cemetery near the village of **Klieščyno** stones marked the head or feet of the buried individuals. One burial involved an incised cross, while another had a stone lining. Again, prolonged functioning and lack of grave goods make dating complicated and assessment relies on mass remains of ceramics interpreted as a commemorative inventory. The site is dated to the 16-18^th^ centuries on the basis of these artefacts. Notably, in this site, children were buried in the existing graves of adult women - a situation similar to that of the contemporary Dubraŭka cemetery [[71]](https://www.zotero.org/google-docs/?eW5iZt).

The late Medieval ground cemetery near the village of **Michalinava** was marked with a stone cross with another cross incised on it, and some stone slabs. Burial pits were hardly recognizable and anatomical order was disrupted in most cases and bones were also found in soil beyond burial features. The archaeologists also noted an unusually large amount of ceramic fragments in the excavated layers. All this supports the prolonged use of the burial ground [[72]](https://www.zotero.org/google-docs/?msxXt5). This makes the dating complicated but Charauko [[72]](https://www.zotero.org/google-docs/?YnqRLz) came to the conclusion that the studied burials belong to the 18-19^th^ centuries, while the functioning of the cemetery began earlier and continued throughout the 15-19^th^ centuries.

**Padsvillie** is a modern town. Two kurgans excavated near it were dated to the 12^th^ century. The village of **Pieravoz** is nearby and has four kurgan groups. Group Pieravoz-4 is accompanied by a ground cemetery with stone structures (S4 Fig). Both kurgans and pit burials were excavated in this complex, including one burial considered transitional from kurgan to stone grave type. As is typical for such complexes, most kurgan burials contained grave goods and are dated to the 11^th^ - beginning of the 12^th^ century. Among the ground interments, several female graves had ornaments of a headdress, while the remaining graves lacked artefacts. The ground part of the cemetery is dated to the 13-14^th^ centuries and is considered to have been used by the incoming Baltic population who buried their deceased next to Kryvičy’s kurgans [[57]](https://www.zotero.org/google-docs/?lieErO).

The kurgan cemetery near to the village of **Sielišča** has cremations and inhumations dated to the end of the 10^th^ - beginning of the 11^th^ century that seem to have belonged to the Balto-Slavic tradition [[117]](https://www.zotero.org/google-docs/?Gza07i). Another kurgan cemetery in the rural area, **Skrabianiec**, is dated to the 11^th^ century [[57]](https://www.zotero.org/google-docs/?Levd5G).

The village of **Vaŭča** gives its name to a further cemetery of a mixed type. All the ground graves are paved, while some are also lined with stones. Research showed that all of the deceased were buried in coffins. Often fires were burned at or near these inhumation graves. This part of the complex is dated to the 13-14^th^ centuries, while kurgans with cremations and inhumations form the earlier part of the cemetery, the overall dating of which spans the 12-14^th^ centuries [[58,59]](https://www.zotero.org/google-docs/?4MDEQ8).

**The city of Polack**

Human and animal material sampled in this study comes from several parts of the city of Polack - Lower Castle, Upper Castle, and Township. Polack’s ‘vakolny horad’ (living quarters that formed a circular city structure), dating to the 9-10^th^ century, was located on the left bank of the river Palata on the territory of an ancient settlement. To the north it was separated by the Palata from the Polack township (“haradzišča” or “dziaciniec”), and in the south it bordered the Upper Castle. In the second half of the 11^th^-12^th^ century it became part of ‘Vialiki pasad’ adjoining the Polack Township, and in 1563 the **Lower Castle** was built in its place [[78,118]](https://www.zotero.org/google-docs/?jP4voO). As a result of this construction, the new Palata riverbed was excavated and the township was cut off from the vakolny horad. During excavations in 2014 archaeologists distinguished three stratigraphic horizons in the Lower Castle area, dated to the 17-18^th^ centuries, 14-16^th^ centuries, and the 11-13^th^ centuries, respectively. Artefacts associated with the Iron Age and early Middle Ages were found, illustrating trade and craft activities of the population of the vakolny horad and Vialiki Pasad of the 11-16^th^ centuries and the existence of an earlier settlement. A complex of items of “družyna” (retinue in the duke’s service) culture of the 9-10^th^ centuries contains some items of Scandinavian origin or their imitations and likely reflects the proto-city period of Polack existence. A large number of items pertain to the Lower Castle of the second half of the 16^th^ -18^th^ centuries. Researchers point out that the material culture of that time characterises the lower property and social status of the inhabitants [[118]](https://www.zotero.org/google-docs/?KoGifa). In an urban setting, cemeteries were commonly formed near churches, and this was the case in the Lower Castle as well. In the 12^th^ century a masonry church was built on its location. A graveyard formed near the church and functioned for a long time, resulting in multi-layered burials of Orthodox inhabitants of the city. In the 17^th^ century, a new graveyard appeared near the church, covering the previous one (S5 Fig). Mostly young male individuals were interred in this later group of burials. This, as well as finds of bullets with some of the deceased, led to the interpretation of them having a military affiliation. The church was probably ruined in the 17^th^ century and the graveyard stopped functioning [[78]](https://www.zotero.org/google-docs/?pYUudi).

The Polack **township** is considered to be the ancient centre of Polack, existing already in the early 9^th^ century, as is evident from the finds of ceramics and dirhams, as well as radiocarbon dating [[119]](https://www.zotero.org/google-docs/?FWFWSP), although it was first inhabited in the Iron Age by the Dnieper-Dvina culture. Polack township in the 10-13^th^ centuries hosted the duke’s residence and potentially a church [[78]](https://www.zotero.org/google-docs/?xsIrkq). In the 17-18^th^ centuries, when the site of the township was already separated from the main plateau by the Palata riverbed, this territory turned into a cemetery, which was excavated in 2007 and 2009 and is interpreted as being Catholic based on the scarce inventory which is mostly limited to tin crosses, and one Jesuit medallion, on the chests of the deceased. Based on the Catholic confession, and on the fact that the cemetery is located in the centre of the city, the researchers concluded that the deceased belonged to Polack nobility. There are not many urban burials dated to the Modern period found so far in Belarus, and in the Dzvina basin there are only two more known apart from the cemetery in the township of Polack [[79]](https://www.zotero.org/google-docs/?n19eNO).

The deceased in the ground necropolis on the territory of the **Upper Castle** belong to various ages and sexes. Most interments were made in wooden coffins, which were made of planks and thin half-logs. Some child burials were made in birch bark, and some of the coffins were covered with birch bark. Apparently, the centre of the cemetery was active for a long time, as multiple layers of burials have been recorded here, while on the periphery the burials are found in one layer at a considerable distance from each other. The grave goods associated with the remains are scarce. Shoes were recorded in some burials and bone combs were found in two. Other objects include slate spindles on the belts of the dead and fragments of glass bracelets. The described inventory dates the cemetery to the 11-13^th^ centuries, with its most active phase of use in the 12^th^ and early 13^th^ centuries. Almost the entire burial site was covered with a layer of construction debris (plinth and limestone) which was formed in the 13-14^th^ centuries. The exact connection of the necropolis to any cult monuments is not recorded, but the presence of a layer of ancient construction debris and individual blocks indirectly suggests the presence of a stone temple, which could have housed a cemetery. However, this hypothesis requires further research.


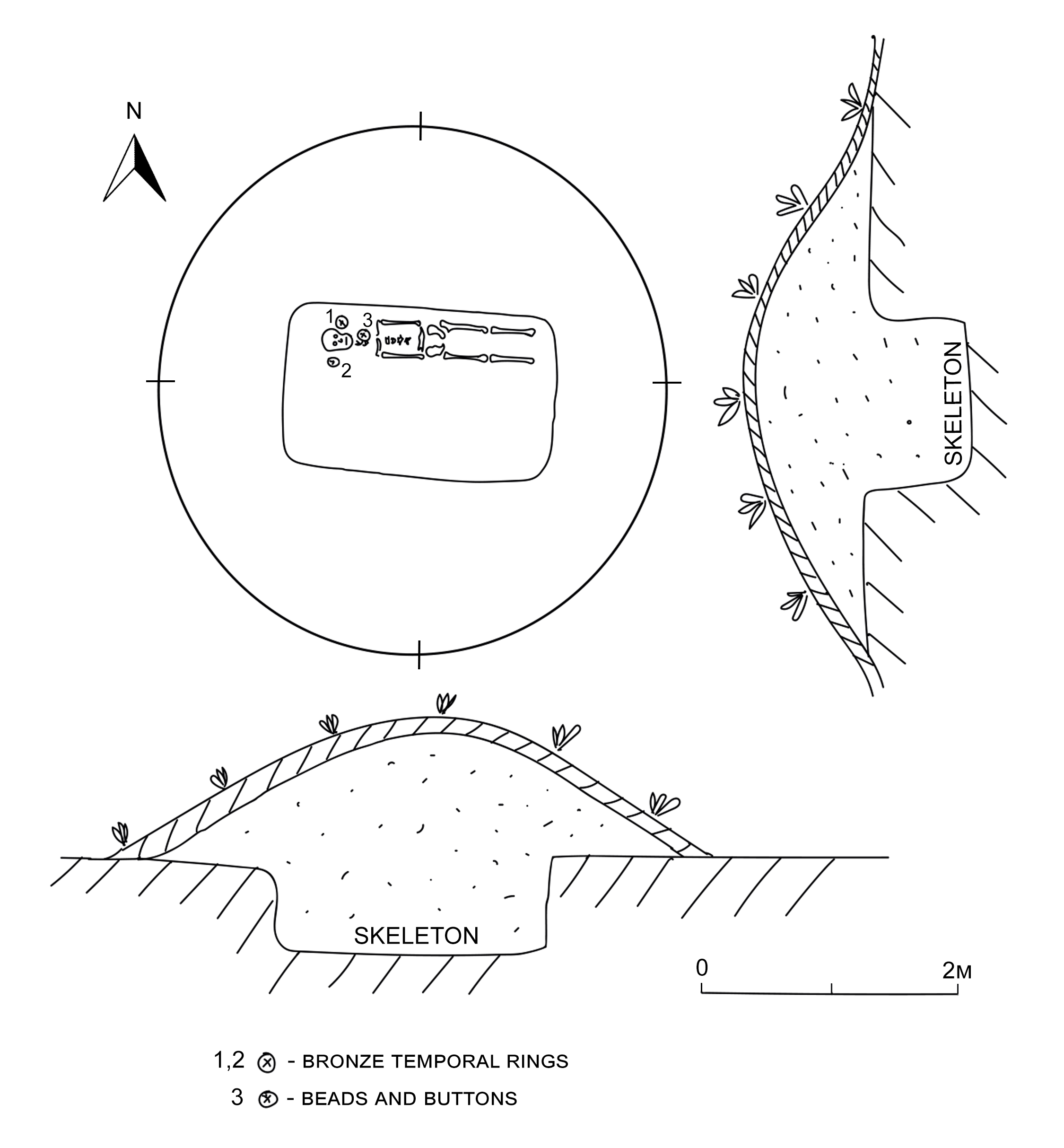


***S1 Fig.*** *Example of a kurgan burial in Skrabianiec (11^th^ century). Individual Skr92-K1. Adapted from Dučyc [[120]](https://www.zotero.org/google-docs/?gqDQMl).*


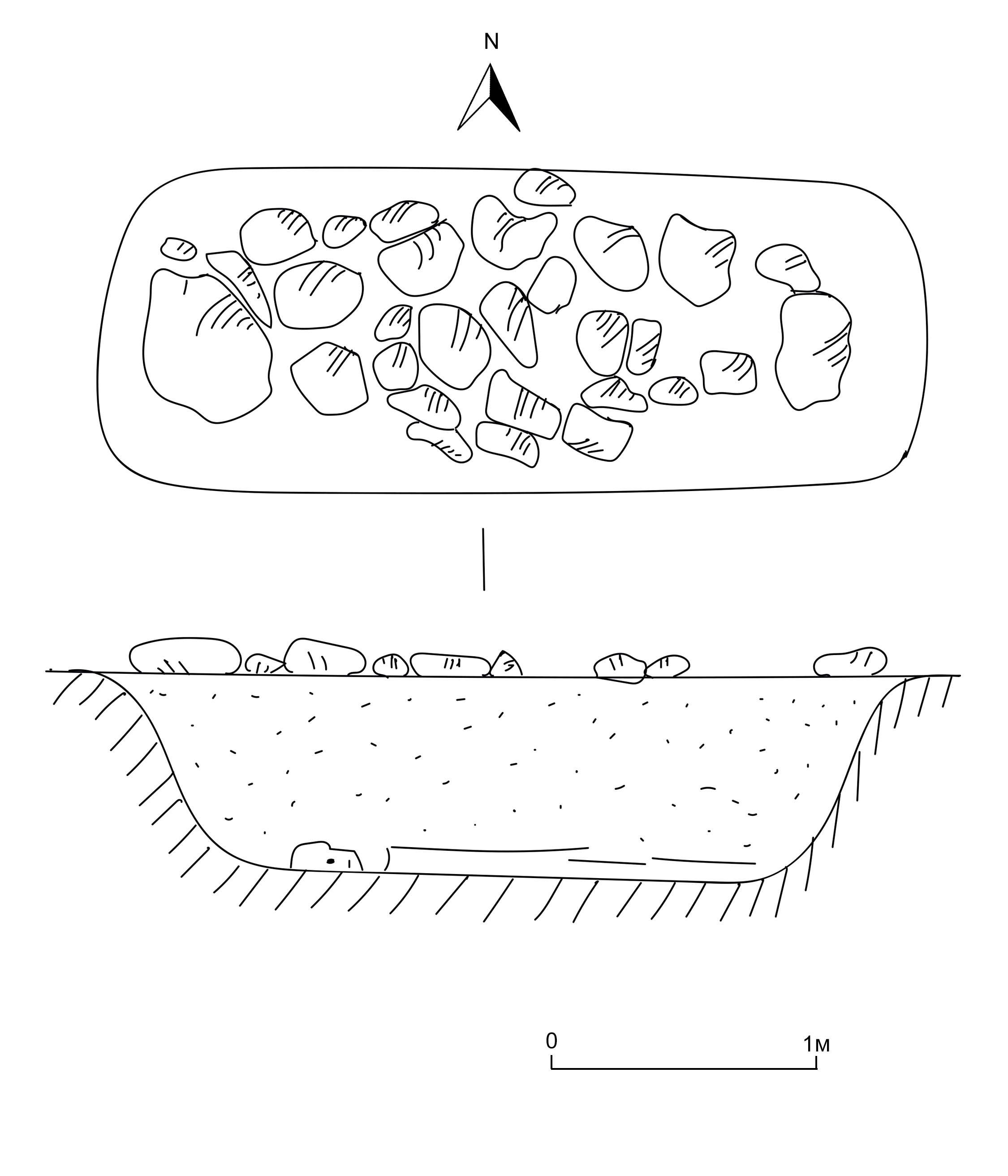


***S2 Fig.*** *Example of a ‘stone’ pit burial in Pieravoz-4 (13-14^th^ century). Individual Pie89-G1. Adapted from Dučyc [[121]](https://www.zotero.org/google-docs/?Yw032t).*


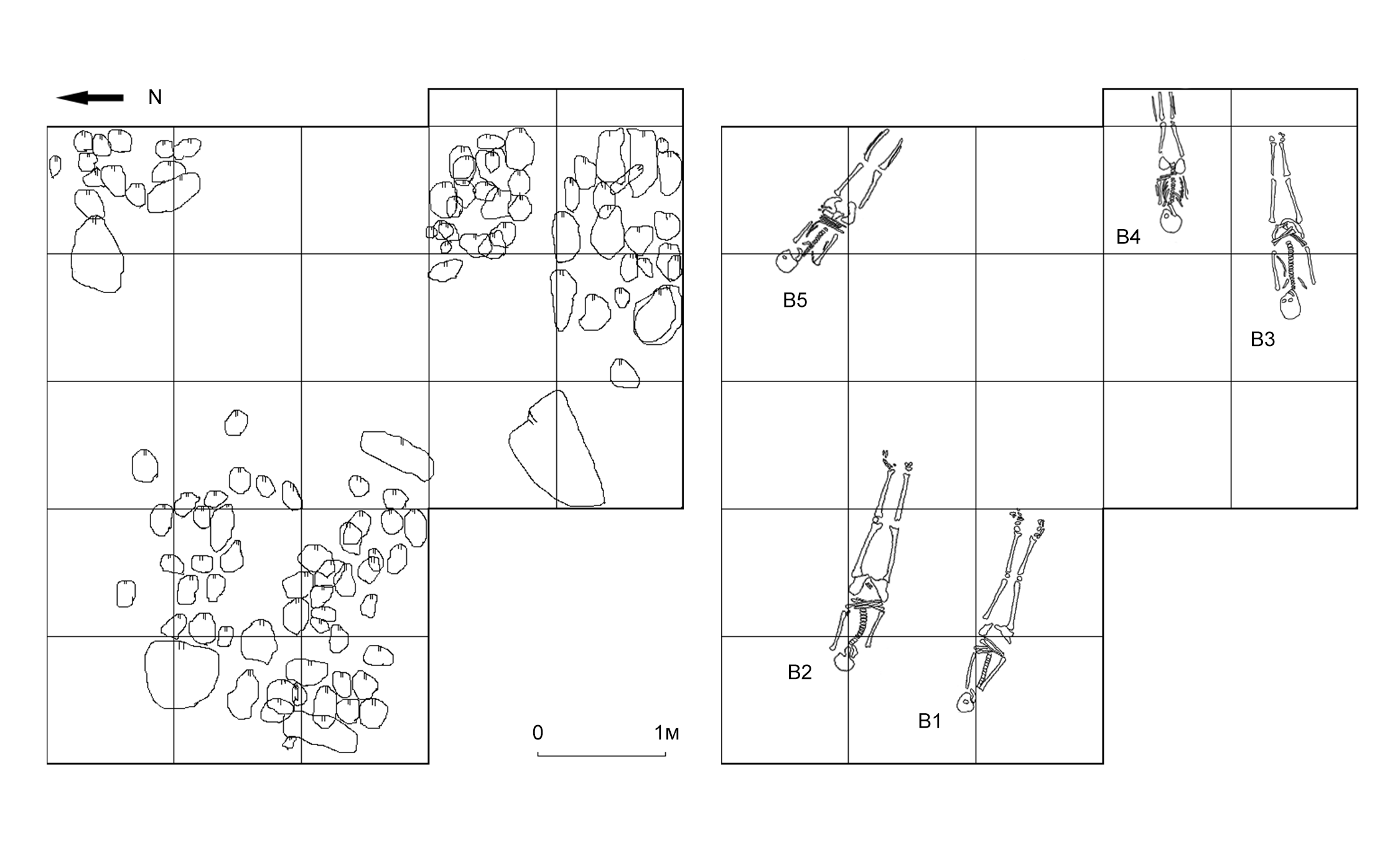


***S3 Fig.*** *Plan of the excavations of the ground cemetery near the village Biruli in 2011: stone constructions (left) and corresponding burials (right) with burial numbers. Adapted from Charauko [[122,123]](https://www.zotero.org/google-docs/?j8iODc).*


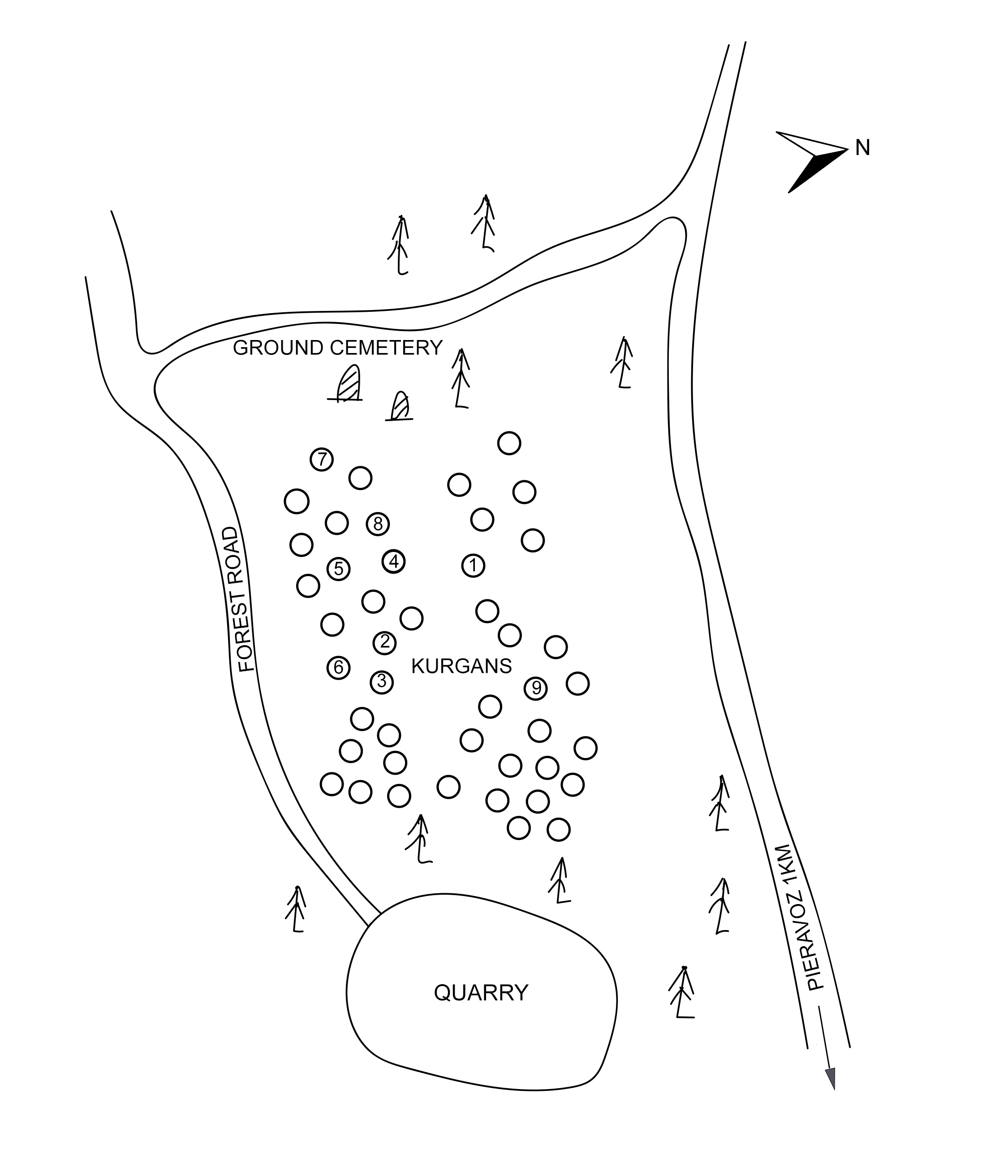


***S4 Fig.*** *Situational plan of the kurgan cemetery #4 near the village Pieravoz with the ground cemetery marked to the north of it. Adapted from Dučyc [[124]](https://www.zotero.org/google-docs/?ty7KBB).*

*
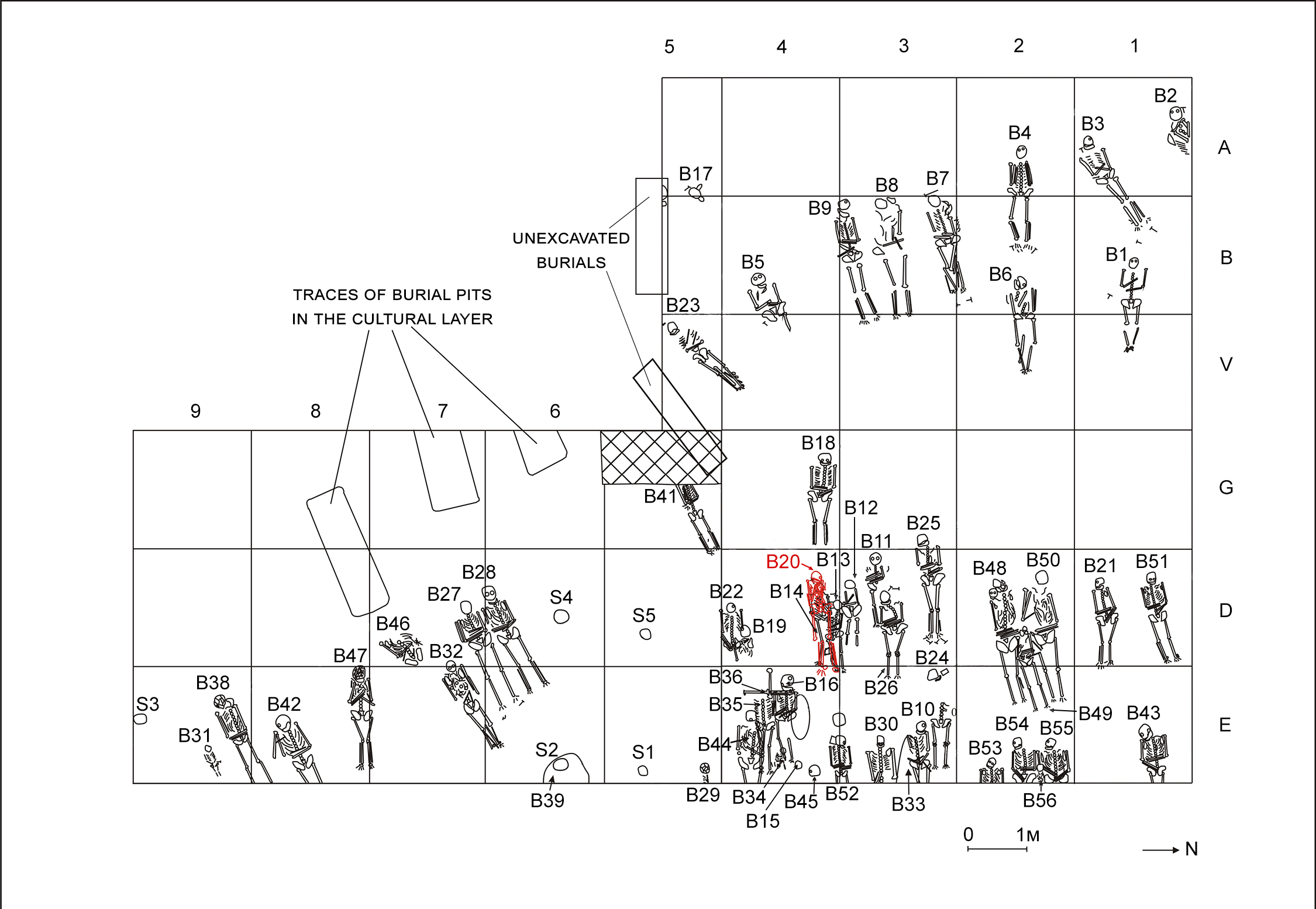
*

***S5 Fig.*** *Plan of the excavations in Polack Lower Castle in 2014. “B” stands for burial and “S” for skull; one skeleton (burial 20) drawn in red for easier distinguishing from the underlying skeleton (burial 14).*


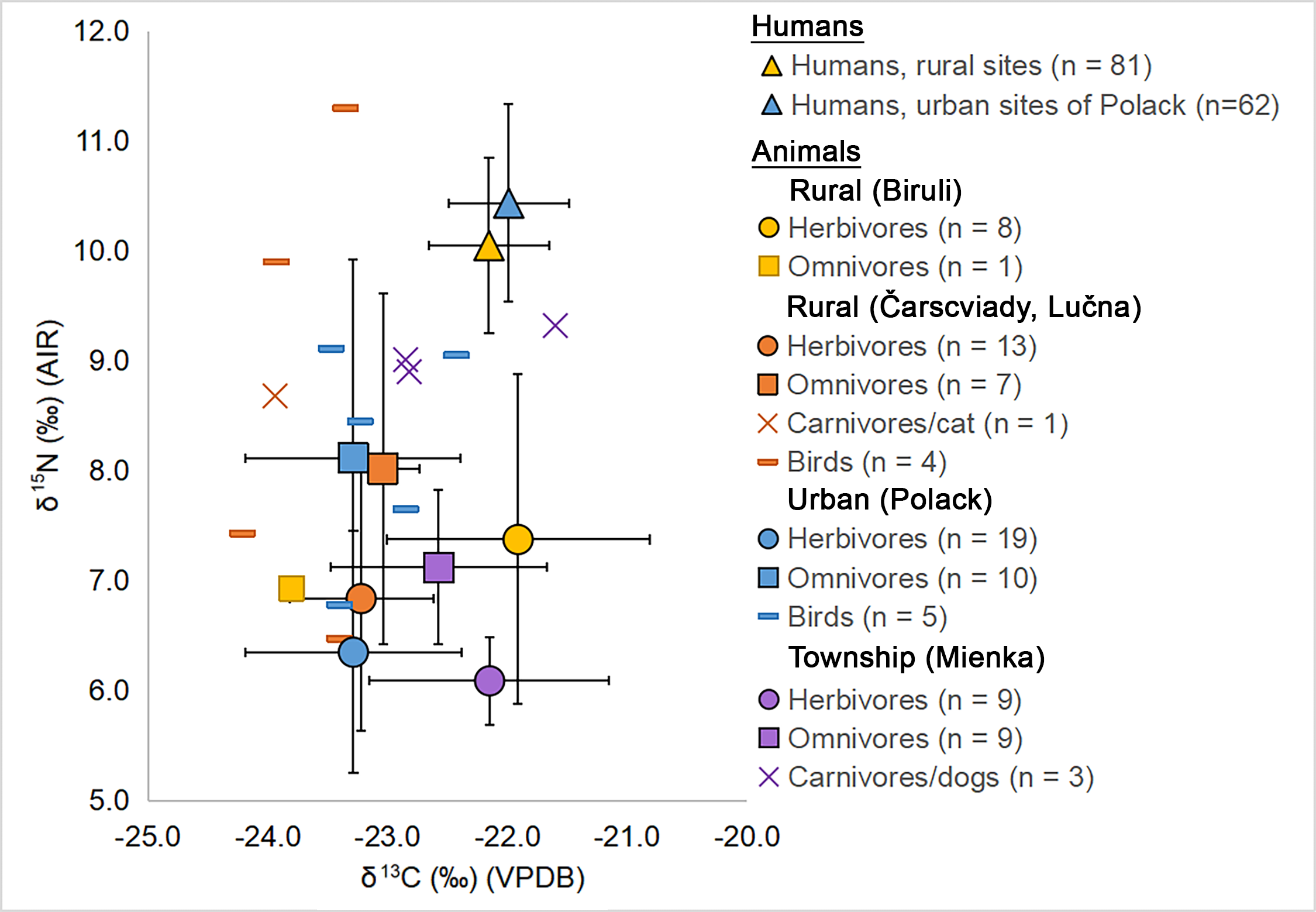


***S6 Fig.*** *Plot of δ^13^C and δ^15^N values of human and animal samples (mean ± 1 std. dev.; individual values for groups where n < 6) grouped by sites. Due to the low number of animal samples from some sites, samples from Čarscviady were considered in the statistical tests and are shown here in a group with Lučna, and Polack Upper Castle together with Lower Castle. Human samples are shown in the two groups which are discussed in the text (all rural sites versus all urban/Polack sites).*

*
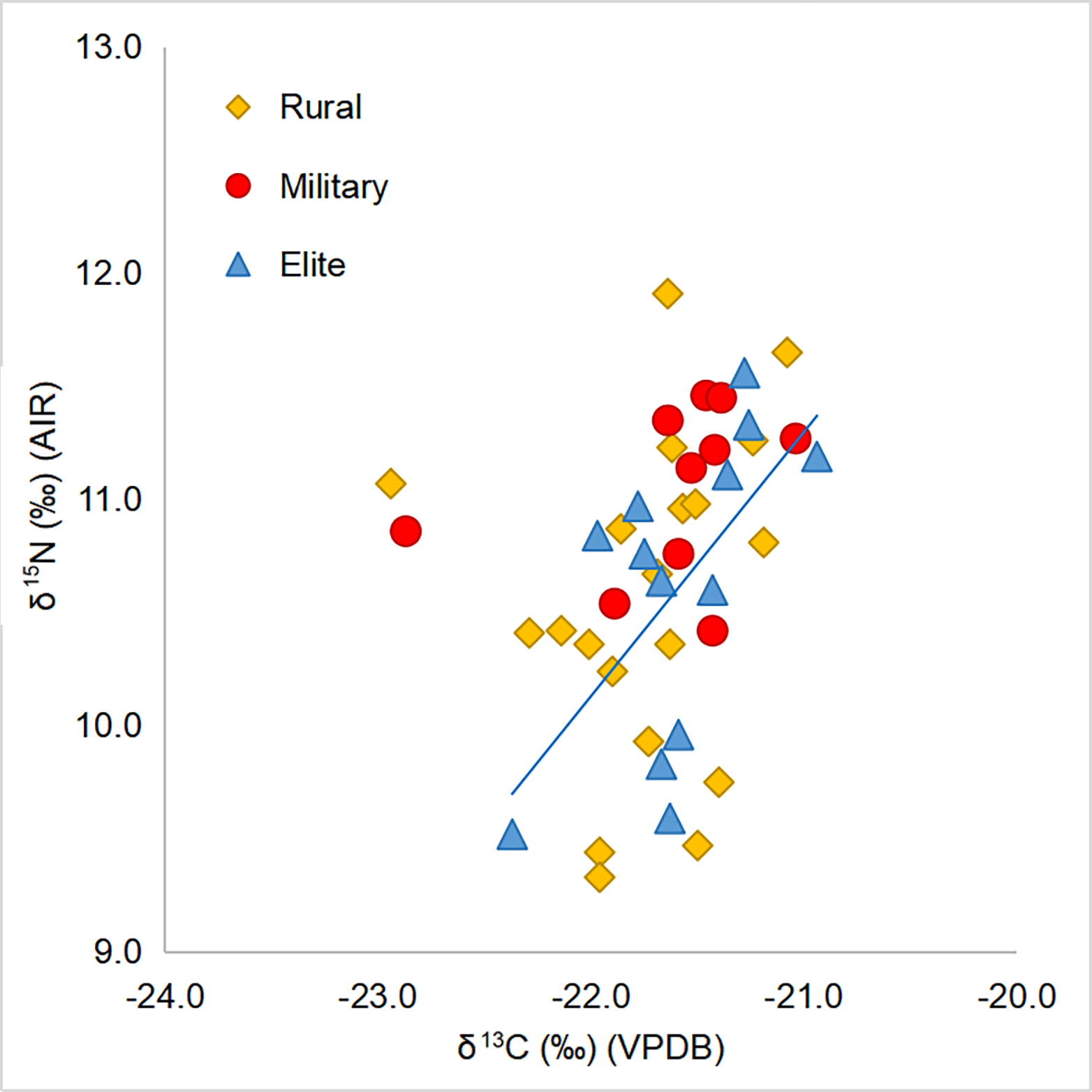
*

***S7 Fig.*** *Plot of δ^13^C and δ^15^N values of human individuals from the elite, military and rural burial contexts of the 16-18^th^ centuries AD with a regression line for the elite sub-sample values.*

**S1 Table.** The stable isotope results of the human samples and the quality control indicators. IDs start with the first three letter of the site name, apart from Polack sites, where PT stands for Polack-Township, PLC for Polack-Lower Castle, and PUC for Polack-Upper Castle.

| **ID** | **sex** | **age** | **sample** | **Coll %** | **δ^15^N (‰) (AIR)** | **δ^13^C (‰) (VPDB)** | **%N** | **%C** | **C/N** |
| --- | --- | --- | --- | --- | --- | --- | --- | --- | --- |
| ***11-13^th^ centuries (n = 44)*** | | | | | | | | | |
| Bir05-K55 | f | adult | M3 | 19.2 | 9.5 | -21.5 | 17.0 | 46.4 | 3.2 |
| Dom79-K11 | f | adult | P2 | 21.1 | 10.1 | -21.8 | 16.1 | 44.9 | 3.3 |
| Dom79-K4 | f | adolescent | M2 | 22.2 | 9.7 | -21.8 | 16.3 | 45.0 | 3.2 |
| Dom79-K5 | m | adult | M3 | 19.1 | 10.6 | -22.2 | 16.3 | 44.9 | 3.2 |
| Dom79-K9 | f | adult | M2 | 19.7 | 9.4 | -21.5 | 16.5 | 45.5 | 3.2 |
| Dzm93-K4 | f | adult | M2 | 20.3 | 9.2 | -22.2 | 16.5 | 45.3 | 3.2 |
| Ivi15-G6 | m | adult | rib | 24.1 | 10.8 | -21.5 | 16.3 | 45.2 | 3.2 |
| Kaz90-G1 | f | adult | M2 | 18.7 | 10.6 | -22.5 | 16.2 | 44.5 | 3.2 |
| Kaz90-G3 | m | adult | P2 | 16.2 | 10.5 | -22.2 | 16.4 | 45.8 | 3.3 |
| Pad91-K1 | m | adult | M3 | 18.4 | 11.1 | -23.5 | 16.4 | 45.3 | 3.2 |
| Pad92-K2-u | m | adult | ulna | 21.3 | 10.4 | -23.3 | 17.1 | 47.7 | 3.2 |
| Pie89-K2 | f | adult | M3 | 17.9 | 10.0 | -21.6 | 16.4 | 45.3 | 3.2 |
| Pie89-K4 | m | adult | M3 | 17.6 | 9.8 | -21.8 | 16.3 | 44.8 | 3.2 |
| Pie91-K10 | m | adult | M2 | 18.0 | 10.4 | -21.7 | 16.4 | 45.1 | 3.2 |
| PUC20-G1 | ? | adolescent | rib | 3.5 | 5.8 | -23.2 | 10.7 | 30.7 | 3.3 |
| PUC20-G14A | f | adult | rib | 17.5 | 11.1 | -23.0 | 16.6 | 46.1 | 3.2 |
| PUC20-G16A-I1 | f | adult | rib | 17.2 | 10.7 | -22.6 | 15.9 | 44.7 | 3.3 |
| PUC20-G16A-I2 | ? | adult | rib | 19.8 | 11.1 | -21.7 | 16.5 | 45.6 | 3.2 |
| PUC20-G16B | f | adult | rib | 18.8 | 9.8 | -21.8 | 16.9 | 46.6 | 3.2 |
| PUC20-G16G | f | adult | rib | 17.6 | 10.0 | -21.1 | 16.5 | 45.1 | 3.2 |
| PUC20-G17G-I1 | m | adult | M2 | 18.9 | 10.2 | -21.8 | 16.2 | 44.8 | 3.2 |
| PUC20-G17G-I2 | f | adult | M3 | 17.4 | 10.9 | -22.3 | 16.5 | 45.7 | 3.2 |
| PUC20-G2 | m | adult | rib | 8.9 | 10.1 | -22.7 | 15.7 | 44.6 | 3.3 |
| PUC20-G20G-I1 | f | adult | rib | 17.6 | 9.6 | -22.4 | 16.6 | 46.5 | 3.3 |
| PUC20-G20G-I2 | ? | adult | M3 | 18.1 | 10.9 | -21.2 | 16.5 | 45.3 | 3.2 |
| PUC20-G21A | n/a | adolescent | rib | 18.4 | 9.5 | -21.5 | 15.9 | 43.6 | 3.2 |
| PUC20-G21B | f | adult | rib | 17.2 | 10.1 | -22.3 | 15.9 | 44.2 | 3.2 |
| PUC20-G21G | m | adult | rib | 17.8 | 11.3 | -22.0 | 16.4 | 45.2 | 3.2 |
| PUC20-G22B | ? | adult | rib | 16.4 | 10.9 | -22.2 | 16.3 | 46.0 | 3.3 |
| PUC20-G23A | m | adult | M2 | 17.2 | 10.2 | -21.8 | 16.5 | 45.2 | 3.2 |
| PUC20-G8 | f | adult | rib | 6.5 | 10.3 | -21.9 | 15.5 | 44.5 | 3.3 |
| PUC20-GII | m | adult | rib | 7.9 | 10.5 | -22.2 | 15.2 | 42.7 | 3.3 |
| PUC20-GV | f | adult | rib | 16.5 | 10.5 | -22.1 | 16.3 | 45.4 | 3.3 |
| PUC20-GVII | f | adult | rib | 10.3 | 9.8 | -22.1 | 15.8 | 44.6 | 3.3 |
| PUC20-GVIII | f | adult | rib | 18.0 | 9.5 | -22.3 | 16.5 | 45.6 | 3.2 |
| Sie90-K11 | m | adult | M3 | 19.6 | 10.3 | -22.0 | 16.2 | 44.9 | 3.2 |
| Sie90-K12 | f | adult | P2 | 21.5 | 9.7 | -23.2 | 16.2 | 45.0 | 3.2 |
| Skr92-K1 | f | adult | femur | 21.3 | 8.9 | -22.2 | 16.8 | 46.3 | 3.2 |
| Skr92-K4 | f | adult | femur | 16.3 | 8.7 | -22.8 | 15.4 | 43.5 | 3.3 |
| Skr92-K5-B1 | m | adult | fibula | 15.9 | 9.0 | -22.3 | 15.2 | 42.1 | 3.2 |
| Vau90-K1 | f | adult | M3 | 20.5 | 11.3 | -22.7 | 16.5 | 46.0 | 3.3 |
| Vau90-K3 | f | adult | M3 | 19.2 | 10.8 | -22.8 | 16.6 | 45.7 | 3.2 |
| Zia95-K3 | m | adult | M2 | 19.6 | 10.7 | -21.6 | 15.9 | 44.1 | 3.2 |
| Zia95-K6 | f | adult | M2 | 20.9 | 10.1 | -22.3 | 16.6 | 45.9 | 3.2 |
| ***13-16^th^ centuries (n = 56)*** | | | | | | | | | |
| Bir08-K86-B1 | m | adult | rib | 21.9 | 9.4 | -22.8 | 16.3 | 45.7 | 3.3 |
| Bir11-K1-B1 | m | adult | rib | 24.5 | 9.8 | -22.0 | 16.6 | 46.1 | 3.3 |
| Bir11-K1-B2 | ? | adult | rib | 19.8 | 9.1 | -22.4 | 16.2 | 45.3 | 3.3 |
| Bir11-K1-B5 | m | adult | rib | 22.1 | 9.8 | -22.5 | 17.4 | 48.6 | 3.3 |
| Bir11-K1-B6 | m | adult | rib | 23.3 | 9.1 | -23.3 | 15.4 | 42.8 | 3.2 |
| Bir11-K1-B7 | m | adult | rib | 22.8 | 9.8 | -22.2 | 16.5 | 46.1 | 3.3 |
| Bir11-T1-B1 | m | adult | rib | 20.1 | 9.3 | -22.5 | 16.6 | 46.2 | 3.2 |
| Bir11-T1-B2 | m | adult | rib | 21.3 | 8.6 | -22.4 | 16.0 | 45.2 | 3.3 |
| Bir11-T1-B5 | f | adult | rib | 20.8 | 8.6 | -22.2 | 16.4 | 45.4 | 3.2 |
| Dou15-G15 | ? | adult | radius | 14.7 | 8.7 | -22.0 | 15.6 | 43.6 | 3.3 |
| Dou15-G23 | f | adult | radius? | 18.0 | 9.0 | -22.2 | 15.0 | 42.5 | 3.3 |
| Dou15-G25 | f | adult | rib | 16.9 | 10.3 | -22.3 | 16.6 | 46.1 | 3.2 |
| Dou15-G28 | m | adult | rib | 21.2 | 9.5 | -22.6 | 16.5 | 46.6 | 3.3 |
| Dou15-G6 | f | adult | rib | 15.5 | 9.1 | -22.1 | 16.6 | 46.3 | 3.3 |
| Ivi11-G5 | m | adult | rib | 20.6 | 9.7 | -22.8 | 17.0 | 47.5 | 3.2 |
| Ivi11-G7 | f | adult | rib | 20.8 | 9.3 | -22.4 | 16.3 | 45.2 | 3.2 |
| Ivi12-G1 | f | adolescent | rib | 16.5 | 9.3 | -22.0 | 16.2 | 44.4 | 3.2 |
| Ivi12-G2 | f | adult | rib | 15.6 | 9.5 | -22.7 | 17.0 | 46.6 | 3.2 |
| Ivi12-G5 | f | adult | rib | 16.4 | 8.9 | -22.3 | 16.5 | 45.9 | 3.2 |
| Ivi13-G1 | m? | adult | rib | 22.2 | 9.3 | -23.0 | 16.5 | 46.0 | 3.3 |
| Ivi13-G2 | m | adult | rib | 20.0 | 10.1 | -22.0 | 16.3 | 45.3 | 3.2 |
| Ivi13-G3 | m | adult | rib | 20.0 | 9.5 | -22.6 | 16.8 | 46.8 | 3.2 |
| Ivi13-G4 | m | adult | rib | 22.2 | 10.4 | -21.5 | 15.8 | 43.9 | 3.2 |
| Ivi15-G1 | m | adult | rib | 20.7 | 9.6 | -22.8 | 16.8 | 46.1 | 3.2 |
| Pie89-G1 | m | adult | M3 | 17.4 | 10.2 | -22.5 | 16.4 | 45.4 | 3.2 |
| Pie91-G10 | m? | adult | M3 | 20.3 | 11.2 | -22.4 | 16.5 | 45.1 | 3.2 |
| Pie91-G11 | ? | adult | M3 | 18.9 | 10.2 | -22.5 | 16.9 | 46.9 | 3.2 |
| Pie91-G15 | f? | adult | M3 | 19.3 | 10.6 | -22.3 | 17.0 | 47.0 | 3.2 |
| Pie91-G7 | f | adult | M2 | 18.1 | 9.4 | -22.0 | 16.8 | 45.8 | 3.2 |
| Pie91-G8 | f | adult | P2 | 18.8 | 10.2 | -22.5 | 16.3 | 45.1 | 3.2 |
| Pie91-G9 | m? | adult | M2 | 16.7 | 10.4 | -22.0 | 16.2 | 45.0 | 3.2 |
| PLC14-G10 | m | adult | rib | 18.7 | 9.1 | -22.0 | 16.6 | 45.2 | 3.2 |
| PLC14-G14 | f? | adult | rib | 20.3 | 11.3 | -22.3 | 16.3 | 44.8 | 3.2 |
| PLC14-G16 | f | adult | rib | 21.3 | 10.2 | -22.4 | 16.2 | 45.0 | 3.2 |
| PLC14-G18-I1 | m | adult | rib | 19.1 | 9.8 | -22.7 | 16.7 | 45.6 | 3.2 |
| PLC14-G20 | m | adult | rib | 19.8 | 11.3 | -22.6 | 16.5 | 45.2 | 3.2 |
| PLC14-G21 | f | adult | rib | 17.1 | 10.3 | -22.8 | 16.3 | 44.2 | 3.2 |
| PLC14-G24 | n/a | adolescent | rib | 21.3 | 10.0 | -22.1 | 16.8 | 46.2 | 3.2 |
| PLC14-G25 | m | adult | rib | 20.3 | 10.7 | -22.1 | 16.4 | 45.1 | 3.2 |
| PLC14-G26 | f | adult | rib | 21.4 | 9.8 | -22.2 | 17.3 | 47.5 | 3.2 |
| PLC14-G30 | m | adult | M2 | 18.0 | 10.8 | -22.2 | 15.9 | 43.9 | 3.2 |
| PLC14-G43 | m | adult | rib | 21.9 | 11.0 | -22.2 | 16.6 | 46.1 | 3.2 |
| PLC14-G44 | m | adult | rib | 21.4 | 9.5 | -22.6 | 17.2 | 47.0 | 3.2 |
| PLC14-G48 | m | adult | rib | 22.1 | 11.0 | -21.9 | 16.3 | 45.1 | 3.2 |
| PLC14-G50 | n/a | adolescent | rib | 19.8 | 10.3 | -22.0 | 16.9 | 46.3 | 3.2 |
| PLC14-G51 | f? | adult | rib | 21.7 | 9.9 | -22.2 | 17.3 | 47.2 | 3.2 |
| PLC14-G53 | m | adult | rib | 22.1 | 9.7 | -23.0 | 16.7 | 46.1 | 3.2 |
| PLC14-G54 | m | adult | rib | 21.8 | 10.4 | -22.3 | 17.2 | 46.8 | 3.2 |
| PLC14-G55 | m | adult | rib | 22.4 | 10.7 | -22.4 | 16.1 | 44.2 | 3.2 |
| Vau90-T1-G2 | m | adult | M2 | 21.3 | 9.7 | -21.7 | 16.2 | 44.4 | 3.2 |
| Vau90-T15 | f | adult | M3 | 20.8 | 10.0 | -22.4 | 16.5 | 46.0 | 3.2 |
| Vau90-T18 | f | adult | M3 | 19.3 | 10.8 | -22.3 | 16.5 | 45.6 | 3.2 |
| Vau90-T2 | m | adult | M3 | 19.8 | 10.6 | -22.3 | 16.5 | 45.3 | 3.2 |
| Vau90-T7 | f | adult | M3 | 18.8 | 10.2 | -22.1 | 16.9 | 46.2 | 3.2 |
| Vau90-T8 | f | adult | M3 | 18.2 | 11.9 | -22.5 | 16.9 | 46.2 | 3.2 |
| Vau90-T9 | f | adult | M3 | 18.5 | 10.2 | -21.9 | 16.4 | 45.9 | 3.3 |
| ***16-18^th^ centuries (n = 43)*** | | | | | | | | | |
| Dub14-G12 | f | adult | rib | 26.6 | 10.8 | -21.2 | 16.1 | 44.3 | 3.2 |
| Dub14-G15 | m | adult | rib | 20.9 | 10.9 | -21.9 | 16.8 | 46.2 | 3.2 |
| Dub14-G2 | f | adult | rib | 21.0 | 9.7 | -21.4 | 16.2 | 45.8 | 3.3 |
| Dub14-G4 | m | adult | rib | 8.9 | 9.9 | -21.7 | 15.4 | 43.3 | 3.3 |
| Dub14-G6 | f | adult | ulna | 22.2 | 10.4 | -22.0 | 16.9 | 46.9 | 3.2 |
| Dub14-G8 | f? | adult | rib | 21.6 | 9.5 | -21.5 | 16.9 | 46.9 | 3.2 |
| Kli13-G2-I1 | f | adult | rib | 25.9 | 11.2 | -21.6 | 16.2 | 44.9 | 3.2 |
| Kli13-G3 | f | adult | rib | 24.3 | 11.3 | -21.2 | 16.9 | 46.9 | 3.2 |
| Kli13-G5 | f | adult | rib | 25.0 | 11.0 | -21.6 | 16.5 | 45.6 | 3.2 |
| Mic16-G11 | f | adolescent | M3 | 19.1 | 11.0 | -21.5 | 16.3 | 44.6 | 3.2 |
| Mic16-G15 | m? | adult | M2 | 20.2 | 10.7 | -21.7 | 16.7 | 46.1 | 3.2 |
| Mic16-G17 | f | adolescent | rib | 23.7 | 9.4 | -22.0 | 16.5 | 45.9 | 3.2 |
| Mic16-G18 | m? | adolescent | rib | 25.4 | 11.1 | -22.9 | 16.9 | 47.2 | 3.3 |
| Mic16-G19 | ? | adult | fibula | 24.6 | 9.3 | -22.0 | 17.0 | 46.7 | 3.2 |
| Mic16-G20 | m? | adult | M2 | 21.9 | 10.4 | -21.6 | 16.2 | 44.6 | 3.2 |
| Mic16-G23 | m | adult | rib | 25.2 | 10.4 | -22.1 | 17.2 | 47.2 | 3.2 |
| Mic16-G25-I1 | m? | adult | M3 | 19.1 | 11.9 | -21.6 | 16.1 | 44.1 | 3.2 |
| Mic16-G3 | ? | adolescent | rib | 22.3 | 10.2 | -21.9 | 16.0 | 44.4 | 3.2 |
| Mic16-G9-I1 | f | adult | M2 | 19.3 | 11.7 | -21.1 | 16.3 | 45.0 | 3.2 |
| Mic16-G9-I2 | f | adult | M3 | 19.9 | 10.4 | -22.3 | 16.3 | 45.3 | 3.2 |
| PLC14-G1 | ? | adult | rib | 7.8 | 10.9 | -22.9 | 16.4 | 45.4 | 3.2 |
| PLC14-G2 | m | adult | rib | 6.5 | 11.3 | -21.0 | 16.2 | 45.2 | 3.3 |
| PLC14-G23 | m | adult | rib | 7.6 | 10.4 | -21.4 | 15.7 | 43.8 | 3.3 |
| PLC14-G3 | m | adolescent | rib | 9.5 | 11.2 | -21.4 | 15.4 | 43.5 | 3.3 |
| PLC14-G4 | m | adult | rib | 13.9 | 11.4 | -21.6 | 16.2 | 45.3 | 3.3 |
| PLC14-G5 | ? | adult | rib | 13.8 | 11.5 | -21.5 | 16.5 | 46.1 | 3.3 |
| PLC14-G6 | m | adult | rib | 12.5 | 11.1 | -21.5 | 16.8 | 46.0 | 3.2 |
| PLC14-G7 | m? | adult | rib | 15.2 | 10.5 | -21.9 | 16.2 | 44.6 | 3.2 |
| PLC14-G8 | m | adult | rib | 17.0 | 11.4 | -21.4 | 17.0 | 46.6 | 3.2 |
| PLC14-G9 | m | adult | rib | 16.5 | 10.8 | -21.6 | 15.8 | 43.5 | 3.2 |
| PT07-G10 | m | adult | rib | 22.1 | 10.8 | -22.0 | 16.8 | 46.6 | 3.2 |
| PT07-G13 | m | adult | rib | 20.8 | 10.8 | -21.8 | 16.3 | 45.3 | 3.3 |
| PT07-G14 | m | adult | rib | 18.2 | 9.6 | -21.6 | 16.3 | 44.8 | 3.2 |
| PT07-G15 | n/a | adolescent | rib | 9.7 | 10.0 | -21.6 | 16.5 | 45.8 | 3.2 |
| PT07-G16 | m | adult | rib | 12.4 | 10.6 | -21.4 | 14.9 | 41.1 | 3.2 |
| PT07-G18 | m | adult | rib | 11.3 | 11.1 | -21.4 | 16.6 | 45.6 | 3.2 |
| PT07-G20 | f | adult | rib | 10.9 | 11.0 | -21.8 | 15.7 | 43.7 | 3.3 |
| PT07-G22 | m | adult | rib | 11.6 | 9.8 | -21.7 | 16.5 | 46.0 | 3.3 |
| PT07-G26 | f | adult | rib | 16.8 | 10.6 | -21.7 | 16.7 | 45.5 | 3.2 |
| PT07-G27 | f? | adult | rib | 20.2 | 11.6 | -21.3 | 16.4 | 45.1 | 3.2 |
| PT07-G3 | m | adult | rib | 24.0 | 9.5 | -22.4 | 16.7 | 46.7 | 3.3 |
| PT07-G5 | f | adult | rib | 23.9 | 11.2 | -20.9 | 16.2 | 44.3 | 3.2 |
| PT07-G7 | f | adult | rib | 15.1 | 11.3 | -21.3 | 16.1 | 44.3 | 3.2 |

**S2 Table.** The stable isotope results of the animal samples and the quality control indicators. Samples omitted from the analysis based on the quality preservation parameters are highlighted in bold and marked with “*”.

| **ID** | **Species** | **Latin** | **Sample** | **Coll %** | **δ^15^N (‰) (AIR)** | **δ^13^C (‰) (VPDB)** | **%N** | **%C** | **C/N** |
| --- | --- | --- | --- | --- | --- | --- | --- | --- | --- |
| ***11-13^th^ centuries (n = 65)*** | | | | | | | | | |
| Bir13-N26.2.01 | cattle | Bos taurus | M1 or M2 | 5.5 | 8.1 | -22.3 | 12.9 | 37.1 | 3.3 |
| Bir13-N29.2.01 | cattle | Bos taurus | M | 4.0 | 7.3 | -22.0 | 10.7 | 29.5 | 3.2 |
| Bir13-N3.2.01 | cattle | Bos taurus | M2 or M3 | 1.2 | 7.3 | -20.6 | 3.1 | 9.0 | 3.4 |
| Bir13-N5.2.01 | sheep/ goat | Ovis sp. | M2 | 2.1 | 8.0 | -21.4 | 8.1 | 22.4 | 3.2 |
| Bir13-N3-4.01 | beaver | Castor fiber l. | M | 3.3 | 5.5 | -22.4 | 4.7 | 13.2 | 3.2 |
| Bir13-N2.01 | goat | Ovis sp. | M2 | **0.3*** | 7.8 | -21.8 | 7.6 | 23.6 | 3.6 |
| Bir13-N5-6.01 | cattle | Bos taurus | M2 | 2.9 | 6.0 | -21.1 | 5.6 | 15.1 | 3.2 |
| Bir13-N5-6.02 | pig | Sus scrofa domesticus | calcaneus | 1.4 | 3.6 | -25.5 | 0.2 | 1.6 | **7.7*** |
| Bir13-N8.1.01 | pig/boar | Sus scrofa domesticus | M1 | 5.1 | 6.9 | -23.8 | 15.1 | 42.7 | 3.3 |
| Bir-N18.1A.01 | horse | Equus ferus caballus | intermediate phalanx | 3.3 | 5.6 | -23.3 | 12.3 | 34.1 | 3.2 |
| Bir-N1-2-7.01 | cattle | Bos taurus | radius | 10.2 | 6.3 | -21.0 | 6.3 | 17.4 | 3.2 |
| Bir-K68.01 | sheep | Ovis sp. | metatarsal | 21.8 | 10.4 | -23.6 | 17.1 | 47.8 | 3.3 |
| Bir14-N1A.01 | pig | Sus scrofa domesticus | rib | 2.6 | 8.2 | -23.6 | 0.2 | 0.5 | **2.6*** |
| Car19-N102 | bison? | Bison bonasus | metacarpal | 12.5 | 4.1 | -23.0 | 9.6 | 26.9 | 3.3 |
| Car19-N187 | pig | Sus scrofa domesticus | M2 | 11.4 | 9.0 | -23.3 | 16.0 | 43.7 | 3.2 |
| Car19-N188 | pig | Sus scrofa domesticus | mandible | 11.3 | 9.1 | -22.9 | 13.3 | 37.2 | 3.3 |
| Car19-N196 | sheep/ goat | Ovis sp. | radius | 10.8 | 7.8 | -22.8 | 16.0 | 45.0 | 3.3 |
| Car19-N199 | cattle | Bos taurus | metacarpal | 6.5 | 8.3 | -23.2 | 13.9 | 39.9 | 3.3 |
| Car19-N205 | wild bird | Aves indet. | tibiotarsus | 15.4 | 8.3 | -26.0 | 16.1 | 46.9 | 3.4 |
| Car20-N1284 | pig | Sus scrofa domesticus | M2 | 9.4 | 5.9 | -23.0 | 14.3 | 40.1 | 3.3 |
| Car20-N1225 | chicken | Gallus gallus domesticus | tarsometatarsal | 14.9 | 9.9 | -23.9 | 16.4 | 46.0 | 3.3 |
| Car20-N1195 | pig | Sus scrofa domesticus | tibia | 9.7 | 6.0 | -22.7 | 15.9 | 45.1 | 3.3 |
| Car20-N1126 | cattle | Bos taurus | distal phalanx | 7.6 | 8.4 | -22.7 | 15.3 | 43.7 | 3.3 |
| Car20-N1153 | hare | Lepus europeaus | femur | 15.0 | 3.2 | -24.8 | 15.1 | 41.8 | 3.2 |
| Car20-N1003 | chicken | Gallus gallus domesticus | radius | 17.9 | 11.3 | -23.3 | 16.1 | 45.6 | 3.3 |
| Car20-N1005 | fish | Pisces indet. | bone fragment | 16.5 | 13.5 | -27.6 | 17.4 | 46.4 | 3.1 |
| Mie20-N6.3.01 | cattle | Bos taurus | metatarsal | 11.8 | 6.1 | -20.8 | 16.0 | 44.1 | 3.2 |
| Mie20-N6.3.05 | pig | Sus scrofa domesticus | C | 2.4 | 7.3 | -22.4 | 11.0 | 30.9 | 3.3 |
| Mie20-N6.3.06 | carnivore (likely dog) | Canis familiaris | rib | 14.4 | 9.0 | -22.8 | 16.3 | 45.0 | 3.2 |
| Mie20-N34.8.01 | dog/ wolf (likely dog) | Canis familiaris | vertebra | 19.7 | 9.3 | -21.6 | 15.9 | 44.6 | 3.3 |
| Mie20-N34.8.04 | pig | Sus scrofa domesticus | intermediate phalanx | 15.6 | 6.6 | -21.7 | 15.6 | 43.0 | 3.2 |
| Mie20-N34.8.02 | cattle | Bos taurus | rib | 13.8 | 5.5 | -22.2 | 16.8 | 46.8 | 3.2 |
| Mie20-N4-3.4.03 | cattle | Bos taurus | mandible | 4.3 | 5.6 | -21.7 | 16.1 | 45.2 | 3.3 |
| Mie20-N4-3.4.04 | pig | Sus scrofa domesticus | maxilla | 6.0 | 7.0 | -24.1 | 15.4 | 43.5 | 3.3 |
| Mie20-N4-3.4.05 | pig | Sus scrofa domesticus | maxilla | 12.3 | 7.6 | -22.2 | 15.6 | 43.4 | 3.3 |
| Mie20-N4-3.4.08 | sheep/ goat | Ovis sp. | mandible | 5.7 | 6.1 | -22.8 | 15.6 | 45.0 | 3.4 |
| Mie20-N1.3.01 | elk | Alces alces l. | M1 or M2 | 9.8 | 5.5 | -23.8 | 16.1 | 44.7 | 3.2 |
| Mie20-N1.3.02 | pig | Sus scrofa domesticus | C | 16.1 | 8.7 | -23.4 | 15.8 | 43.5 | 3.2 |
| Mie20-N1.3.04 | pig | Sus scrofa domesticus | C | 11.5 | 6.7 | -22.4 | 15.6 | 43.3 | 3.2 |
| Mie20-N1.3.05 | cattle | Bos taurus | metatarsal | 12.0 | 6.6 | -23.3 | 12.1 | 33.6 | 3.2 |
| Mie20-N4-3.4.13 | elk | Alces alces l. | tibia | 4.8 | 6.9 | -22.5 | 15.1 | 42.4 | 3.3 |
| Mie20-N4-3.4.15 | carnivore (likely dog) | Canis familiaris | rib | 19.7 | 8.9 | -22.8 | 14.5 | 40.2 | 3.2 |
| Mie20-N4-3.4.14 | cattle | Bos taurus | metatarsal | 13.8 | 6.0 | -22.3 | 16.6 | 45.8 | 3.2 |
| Mie20-N4-3.4.01 | pig | Sus scrofa domesticus | tibia | 12.9 | 6.9 | -21.4 | 15.5 | 43.3 | 3.3 |
| Mie20-N4-3.4.02 | cattle | Bos taurus | humerus | 7.3 | 6.1 | -22.8 | 11.3 | 32.0 | 3.3 |
| Mie20-ST2-N3 | horse | Equus ferus caballus | P3 or P4 | 15.1 | 6.6 | -23.2 | 15.2 | 41.7 | 3.2 |
| Mie20-ST2-N1 | cattle | Bos taurus | M2 | 10.1 | 6.2 | -20.4 | 15.4 | 42.6 | 3.2 |
| Mie20-ST2-N2 | pig | Sus scrofa domesticus | M3 | 2.4 | 6.4 | -23.4 | 14.2 | 39.0 | 3.2 |
| Mie20-ST2-N4 | pig | Sus scrofa domesticus | mandible | 6.3 | 6.9 | -22.4 | 12.6 | 35.9 | 3.3 |
| PLC14-N24.G1.04 | cattle | Bos taurus | metatarsal | 19.8 | 5.1 | -22.3 | 14.9 | 41.2 | 3.2 |
| PLC14-N24.G1.01 | pig/boar | Sus scrofa domesticus | maxilla | 6.5 | 7.1 | -23.6 | 15.1 | 43.5 | 3.4 |
| PLC14-N24.G1.02 | beaver | Castor fiber l. | ulna | 15.5 | 2.8 | -23.1 | 16.3 | 45.3 | 3.2 |
| PLC14-N24.G1.05 | pig | Sus scrofa domesticus | M2 | 9.6 | 10.7 | -25.3 | 16.1 | 44.9 | 3.2 |
| PLC14-N2265.02 | pig | Sus scrofa domesticus | C | 12.3 | 4.5 | -23.0 | 15.9 | 45.2 | 3.3 |
| PLC14-N2265.06 | cattle | Bos taurus | metacarpal | 19.6 | 6.1 | -22.2 | 16.1 | 43.6 | 3.2 |
| PLC14-N2265.07 | pike | Esox lucius | dentale | 5.9 | 11.6 | -27.7 | 12.8 | 36.1 | 3.3 |
| PLC14-N2391.01 | pike | Esox lucius | dentale | 13.1 | 10.9 | -28.2 | 16.1 | 43.9 | 3.2 |
| PLC14-N2391.08 | pig | Sus scrofa domesticus | tibia | 13.7 | 9.1 | -23.1 | 16.0 | 44.3 | 3.2 |
| PLC14-N2391.04 | chicken | Gallus gallus domesticus | femur | 15.4 | 7.7 | -22.8 | 16.0 | 44.7 | 3.3 |
| PLC14-N2391.05 | chicken | Gallus gallus domesticus | femur | 11.8 | 9.1 | -23.5 | 16.0 | 45.5 | 3.3 |
| PLC14-N2391.06 | galliformes | Galliformes indet. | coracoid | 15.9 | 8.5 | -23.2 | 15.1 | 43.1 | 3.3 |
| PLC14-N2391.07 | sheep | Ovis sp. | tibia | 16.3 | 8.9 | -23.2 | 15.2 | 42.6 | 3.3 |
| PLC14-NE1.5.02 | cattle | Bos taurus | intermediate phalanx | 7.1 | 5.9 | -23.4 | 15.4 | 44.4 | 3.4 |
| PLC14-NE2.5.01 | cattle | Bos taurus | proximal phalanx | 10.8 | 5.9 | -22.6 | 9.8 | 27.0 | 3.2 |
| PLC14-NE2.5.04 | sheep | Ovis sp. | tibia | 11.2 | 7.2 | -23.4 | 15.6 | 42.9 | 3.2 |
| ***13-16^th^ centuries (n = 25)*** | | | | | | | | | |
| Luc01-N27.2.01 | rabbit | Oryctolagus cuniculus | maxilla | 10.5 | 5.6 | -24.6 | 15.3 | 42.8 | 3.3 |
| Luc01-N27.2.02 | goose | Anser anser | beak | 15.9 | 6.5 | -23.4 | 16.8 | 47.0 | 3.3 |
| Luc01-N851 | sheep/ goat | Ovis sp. | metatarsal | 21.2 | 5.6 | -23.8 | 17.0 | 46.6 | 3.2 |
| Luc02-N1565 | cattle | Bos taurus | M2 | 8.1 | 6.3 | -23.0 | 12.5 | 36.1 | 3.4 |
| Luc02-N1768 | cattle | Bos taurus | metacarpal | 9.7 | 6.3 | -23.5 | 16.0 | 45.1 | 3.3 |
| Luc02-N1840 | cat | Felix sp. | femur | 19.2 | 8.7 | -23.9 | 15.8 | 44.6 | 3.3 |
| Luc05-N8030 | sheep/ goat | Ovis sp. | M2 or M1 | 5.0 | 8.7 | -23.1 | 15.6 | 42.8 | 3.2 |
| Luc05-N3102 | pig | Sus scrofa domesticus | mandible | 19.2 | 7.6 | -23.4 | 16.2 | 45.1 | 3.2 |
| Luc05-N3057 | cattle | Bos taurus | tibia | 14.1 | 5.3 | -22.6 | 11.9 | 33.5 | 3.3 |
| Luc05-N3058 | sheep/ goat | Ovis sp. | humerus | 12.8 | 7.1 | -22.9 | 16.7 | 47.0 | 3.3 |
| Luc05-N3130 | pig | Sus scrofa domesticus | tibia | 6.9 | 9.9 | -23.1 | 11.7 | 33.9 | 3.4 |
| Luc-S10-L3-N1 | cattle | Bos taurus | mandible | 4.2 | 6.1 | -23.1 | 15.6 | 44.9 | 3.3 |
| Luc-N10.3.03 | pig | Sus scrofa domesticus | vertebra | 14.8 | 8.7 | -22.9 | 16.1 | 44.9 | 3.3 |
| Luc-N10.3.04 | goose | Anser anser | vertebra | 19.9 | 7.4 | -24.2 | 16.2 | 46.3 | 3.3 |
| PLC14-NA1.2.01 | cattle | Bos taurus | M1 | 9.9 | 7.3 | -22.9 | 16.2 | 44.4 | 3.2 |
| PLC14-NB1.3.02 | sheep/ goat | Ovis sp. | mandible | 16.4 | 7.0 | -22.6 | 16.8 | 46.4 | 3.2 |
| PLC14-NB1.3.01 | rabbit | Oryctolagus cuniculus | tibia | 14.8 | 4.7 | -25.4 | 16.7 | 45.9 | 3.2 |
| PLC14-NB3.4.01 | cattle | Bos taurus | M2 or M1 | 17.4 | 7.3 | -23.1 | 15.7 | 43.6 | 3.2 |
| PLC14-NB3.4.02 | goat | Ovis sp. | metacarpal | 8.8 | 5.2 | -23.3 | 15.7 | 43.0 | 3.2 |
| PLC14-NB4.4.01 | cattle | Bos taurus | mandible | 12.9 | 6.7 | -23.6 | 16.2 | 44.8 | 3.2 |
| PLC14-NB4.4.02 | horse | Equus ferus caballus | metacarpal | 9.6 | 6.7 | -21.2 | 11.1 | 30.0 | 3.1 |
| PLC14-NB4.4.04 | pig | Sus scrofa domesticus | intermediate phalanx | 20.3 | 7.4 | -23.8 | 16.8 | 46.4 | 3.2 |
| PUC20-N1.02 | elk | Alces alces l. | metatarsal | 19.9 | 4.3 | -24.8 | 16.5 | 45.2 | 3.2 |
| PUC20-N1.03 | cattle | Bos taurus | skull | 21.8 | 6.5 | -23.6 | 17.2 | 47.1 | 3.2 |
| PUC20-N1.04 | horse | Equus ferus caballus | scapula | 22.7 | 4.8 | -24.1 | 15.7 | 44.5 | 3.3 |
| ***16-18^th^ centuries (n = 15)*** | | | | | | | | | |
| Luc-N2/382 | cattle | Bos taurus | M2 | 3.2 | 7.3 | -22.7 | 10.3 | 28.0 | 3.2 |
| Luc-N1916 | horse/ pony | Equus ferus caballus | distal phalanx | 26.4 | 6.1 | -23.7 | 16.2 | 45.1 | 3.2 |
| PLC14-NG5.1.01 | pig | Sus scrofa domesticus | P4 | 12.0 | 7.6 | -23.8 | 15.8 | 44.1 | 3.3 |
| PLC14-NG5.1.02 | pig | Sus scrofa domesticus | M2 | 17.2 | 9.9 | -22.5 | 16.0 | 43.9 | 3.2 |
| PLC14-NG5.1.03 | pig | Sus scrofa domesticus | humerus | 12.0 | 9.2 | -22.5 | 15.7 | 44.2 | 3.3 |
| PLC14-NG5.1.04 | cattle | Bos taurus | femur | 16.7 | 5.6 | -23.6 | 16.1 | 44.8 | 3.3 |
| PLC14-NG5.1.05 | red deer | Cervus sp. | radius | 8.5 | 3.9 | -23.0 | 10.6 | 29.5 | 3.3 |
| PLC14-NG2-D2.2.01 | chicken | Gallus gallus domesticus | tarsometatarsal | 18.4 | 9.1 | -22.4 | 15.8 | 44.7 | 3.3 |
| PLC14-NG2-D2.2.02 | galliformes | Galliformes indet. | femur | 15.6 | 6.8 | -23.4 | 16.4 | 46.2 | 3.3 |
| PLC14-NG2-D2.2.03 | sheep/goat | Ovis sp. | scapula | 13.7 | 7.0 | -23.5 | 16.2 | 45.0 | 3.2 |
| PUC16-NA3.13.01 | horse | Equus ferus caballus | metatarsal | 13.3 | 7.1 | -23.7 | 16.6 | 46.6 | 3.3 |
| PUC16-NB1.3.01 | horse | Equus ferus caballus | distal phalanx | 16.6 | 5.6 | -24.7 | 15.9 | 44.7 | 3.3 |
| PUC16-NB3.12.01 | pig | Sus scrofa domesticus | fibula | 17.8 | 6.7 | -22.9 | 17.2 | 47.2 | 3.2 |
| PUC16-NV2.3.02 | pig | Sus scrofa domesticus | ulna | 9.9 | 9.0 | -22.4 | 15.8 | 44.6 | 3.3 |
| PUC16-NV2.3.03 | cattle | Bos taurus | distal phalanx | **0*** | 5.7 | -24.3 | 0.3 | 2.2 | **7.7*** |

**S3 Table.** Results of post-hoc Bonferroni Tests for multiple pairwise comparisons: δ^13^C values of domestic herbivores by group of sites. Bold and “*” show where the p-value is less than 0.05.

| **(Group of) sites** | **Biruli** | **Polack** | **Lučna and Čarscviady** |
| --- | --- | --- | --- |
| **Polack** | **0.004*** | - | - |
| **Lučna and Čarscviady** | **0.012*** | 1.000 | - |
| **Mienka** | 1.000 | **0.017*** | **0.048*** |

**S4 Table.** Kruskal-Wallis multiple comparisons (2-tailed): δ^15^N values of domestic herbivores by group of sites. All p>0.05.

| **(Group of) sites** | **Biruli** | **Polack** | **Lučna and Čarscviady** |
| --- | --- | --- | --- |
| **Polack** | 0.386 | - | - |
| **Lučna and Čarscviady** | 1.000 | 1.000 | - |
| **Mienka** | 0.193 | 1.000 | 0.664 |

**S5 Table.** Kruskal-Wallis multiple comparisons (2-tailed): δ^13^C values of domestic herbivores by time periods. Bold and “*” show where the p-value is less than 0.05.

| **Period** | **11-13^th^ c. AD** | **13-16^th^ c. AD** |
| --- | --- | --- |
| **13-16^th^ c. AD** | **0.008*** | - |
| **16-18^th^ c. AD** | **0.004*** | 0.815 |

**S6 Table.** Kruskal-Wallis multiple comparisons (2-tailed): δ^15^N values of domestic herbivores by time periods. All p>0.05.

| **Period** | **11-13^th^ c. AD** | **13-16^th^ c. AD** |
| --- | --- | --- |
| **13-16^th^ c. AD** | 0.870 | - |
| **16-18^th^ c. AD** | 1.000 | 1.000 |

**S7 Table.** Kruskal-Wallis multiple comparisons (2-tailed): δ^13^C values of domestic herbivores by species. Bold and “*” show where the p-value is less than 0.05.

| **Species** | **Cattle** | **Sheep/goat** |
| --- | --- | --- |
| **Sheep/goat** | 0.199 | - |
| **Horse/pony** | **0.015*** | 0.731 |

**S8 Table.** Kruskal-Wallis multiple comparisons (2-tailed): δ^15^N values of domestic herbivores by species. All p>0.05.

| **Species** | **Cattle** | **Sheep/goat** |
| --- | --- | --- |
| **Sheep/goat** | 0.171 | - |
| **Horse/pony** | 1.000 | 0.115 |

**S9 Table.** Kruskal-Wallis multiple comparisons (2-tailed): δ^13^C values of human individuals by time periods. Bold and “*” show where the p-value is less than 0.05.

| **Period** | **11-13^th^ c. AD** | **13-16^th^ c. AD** |
| --- | --- | --- |
| **13-16^th^ c. AD** | 0.067 | - |
| **16-18^th^ c. AD** | **0.000*** | **0.000*** |

**S10 Table.** Kruskal-Wallis multiple comparisons (2-tailed): δ^15^N values of human individuals by time periods. Bold and “*” show where the p-value is less than 0.05.

| **Period** | **11-13^th^ c. AD** | **13-16^th^ c. AD** |
| --- | --- | --- |
| **13-16^th^ c. AD** | 0.380 | - |
| **16-18^th^ c. AD** | **0.004*** | **0.000*** |

**S11 Table.** Kruskal-Wallis multiple comparisons (2-tailed): δ^13^C values of 16-18^th^-century human individuals by context of the burial (elite, military, non-elite). All p>0.05.

| **Context of burial** | **non-elite** | **military** |
| --- | --- | --- |
| **military** | 0.607 | - |
| **elite** | 1.000 | 1.000 |

**S12 Table.** Results of post-hoc Bonferroni Tests for multiple pairwise comparisons: δ^15^N values of 16-18^th^-century human individuals by context of the burial (elite, military, non-elite). All p>0.05.

| **Context of burial** | **non-elite** | **military** |
| --- | --- | --- |
| **military** | 0.170 | - |
| **elite** | 1.000 | 0.342 |

**S13 Table.** Kruskal-Wallis multiple comparisons (2-tailed): δ^13^C values of domestic herbivores by country. Bold and “*” show where the p-value is less than 0.05.

| **Country** | **Belarus** | **Poland** |
| --- | --- | --- |
| **Poland** | **0.000*** | - |
| **Lithuania** | **0.006*** | 0.287 |

**S14 Table.** Kruskal-Wallis multiple comparisons (2-tailed): δ^15^N values of domestic herbivores by country. All p>0.05.

| **Country** | **Belarus** | **Poland** |
| --- | --- | --- |
| **Poland** | 1.000 | - |
| **Lithuania** | 1.000 | 0.905 |

**S15 Table.** Kruskal-Wallis multiple comparisons (2-tailed): δ^13^C values of human individuals from different countries by groups of sites. Bold and “*” show where the p-value is less than 0.05.

| **(Group of) sites** | **Belarusi-an rural** | **Polack** | **Kałdus** | **Gruczno** | **Polish elites** | **Lithuanian elites** | **Lithuanian rural** | **Smeltė** |
| --- | --- | --- | --- | --- | --- | --- | --- | --- |
| **Polack** | 1.000 | - | - | - | - | - | - | - |
| **Kałdus** | **0.000*** | **0.000*** | - | - | - | - | - | - |
| **Gruczno** | **0.000*** | **0.000*** | **0.021*** | - | - | - | - | - |
| **Polish elites** | **0.000*** | **0.000*** | 1.000 | 1.000 | - | - | - | - |
| **Lithuani-an elites** | **0.000*** | **0.000*** | **0.019*** | 1.000 | 1.000 | - | - | - |
| **Lithuani-an rural** | **0.007*** | **0.027*** | **0.021*** | 1.000 | 0.406 | 1.000 | - | - |
| **Smeltė** | **0.001*** | **0.007*** | **0.000*** | 0.097 | **0.008*** | 0.510 | 1.000 | - |
| **Alytus** | **0.000*** | **0.000*** | **0.000*** | 1.000 | 0.083 | 1.000 | 1.000 | 1.000 |

**S16 Table.** Kruskal-Wallis multiple comparisons (2-tailed): δ^15^N values of human individuals from different countries by groups of sites. Bold and “*” show where the p-value is less than 0.05.

| **(Group of) sites** | **Belarusi-an rural** | **Polack** | **Kałdus** | **Gruczno** | **Polish elites** | **Lithuanian elites** | **Lithuanian rural** | **Smeltė** |
| --- | --- | --- | --- | --- | --- | --- | --- | --- |
| **Polack** | 0.206 | - | - | - | - | - | - | - |
| **Kałdus** | 1.000 | 1.000 | - | - | - | - | - | - |
| **Gruczno** | **0.001*** | **0.000*** | **0.000*** | - | - | - | - | - |
| **Polish elites** | **0.000*** | **0.000*** | **0.000*** | **0.000*** | - | - | - | - |
| **Lithuani-an elites** | **0.000*** | **0.000*** | **0.000*** | **0.000*** | 1.000 | - | - | - |
| **Lithuani-an rural** | 1.000 | 1.000 | 1.000 | 0.282 | **0.001*** | **0.026*** | - | - |
| **Smeltė** | **0.000*** | **0.000*** | **0.000*** | **0.000*** | 1.000 | 1.000 | **0.000*** | - |
| **Alytus** | 0.313 | 1.000 | 1.000 | **0.000*** | **0.000*** | **0.000*** | 1.000 | **0.000*** |

**S17 Table.** Kruskal-Wallis multiple comparisons (2-tailed): δ^13^C values of elite subsamples from different countries and military subsample from Belarus. Bold and “*” show where the p-value is less than 0.05.

| **Country** | **Belarusian military** | **Belarusian elite** | **Polish elite** |
| --- | --- | --- | --- |
| **Belarusian elite** | 1.000 | - | - |
| **Polish elite** | **0.000*** | **0.000*** | - |
| **Lithuanian elite** | **0.001*** | **0.000*** | **0.024*** |

**S18 Table.** Kruskal-Wallis multiple comparisons (2-tailed): δ^15^N values of elite subsamples from different countries and military subsample from Belarus. Bold and “*” show where the p-value is less than 0.05.

| **Country** | **Belarusian military** | **Belarusian elite** | **Polish elite** |
| --- | --- | --- | --- |
| **Belarusian elite** | 1.000 | - | - |
| **Polish elite** | **0.000*** | **0.000*** | - |
| **Lithuanian elite** | 0.210 | **0.003*** | **0.001*** |

**S1 References**

[106. World Bank Climate Change Knowledge Portal. In: Climateknowledgeportal.worldbank.org [Internet]. 2022 [cited 7 Oct 2022]. Available: https://climateknowledgeportal.worldbank.org/country/belarus](https://www.zotero.org/google-docs/?yfeJGw)

[107. Loshenkov MI. Arkheobotanicheskiye kollektsii Belarusi. Minsk: Izdatel’skiy tsentr BGU; 2021.](https://www.zotero.org/google-docs/?yfeJGw)

[108. Makushnikov OA, Loshenkov MI. Paleobotanicheskiye materialy iz srednevekovogo Gomiya. Materyjaly Pa Arch Bielarusi. 2016;28: 230–238.](https://www.zotero.org/google-docs/?yfeJGw)

[109. Lakotka AI. Uvodziny. In: Narysy historyi kuĺtury Bielarusi U 4 t T 3 Kuĺtura siala CHIV – pačatku CHCH st Kn 1 Materyjaĺnaja kuĺtura. Minsk: Belaruskaia navuka; 2015. pp. 5–7.](https://www.zotero.org/google-docs/?yfeJGw)

[110. Piličiauskienė G, Blaževičius P. Archaeoichthyological and Historical Data on Fish Consumption in Vilnius Lower Castle during the 14](https://www.zotero.org/google-docs/?yfeJGw)^[th](https://www.zotero.org/google-docs/?yfeJGw)^[-17](https://www.zotero.org/google-docs/?yfeJGw)^[th](https://www.zotero.org/google-docs/?yfeJGw)^ [Centuries. Est J Archaeol. 2019;23: 39–55. doi:10.3176/arch.2019.1.03](https://www.zotero.org/google-docs/?yfeJGw)

[111. Zaharuĺski EM. Zachodniaja Ruś. IX-XIII stst. Minsk: Univiersiteckaje; 1998.](https://www.zotero.org/google-docs/?yfeJGw)

[112. Levko ON. Formirovaniye torgovykh otnosheniy v Belorusskom Podvin’ye (X―XVIII). In: Historyja handliu na terytoryi Bielarusi: zbornik navukovych artykulaŭ. Minsk: Belaruskaia navuka; 2016. pp. 10–20.](https://www.zotero.org/google-docs/?yfeJGw)

[113. Gud DA. Srednevekovyy Polotsk na vodnom puti “iz varyag v greki”: aspekty razvitiya torgovli i material’noy kul’tury. Tr Molodykh Spetsialistov Polotskogo Gos Univ. 2007;20: 84–85.](https://www.zotero.org/google-docs/?yfeJGw)

[114. Gruzitskiy YL. Tamozhennyye otnosheniya v Velikom knyazhestve Litovskom (XV―XVI vv.). In: Historyja handliu ŭ Bielarusi (ad staražytnaha času da kanca XX st): prabliemy vyvučennia i pierspiektyvy dasliedavannia : materyjaly I Mižnarodnaj navukova-praktyčnaj kanfierencyi. Minsk: Technalohija; 2014. pp. 74–81.](https://www.zotero.org/google-docs/?yfeJGw)

[115. Sliž NU. Arhanizacyja handiu ŭ Horadni ŭ XVI ― pieršaj palovie XVII stahoddzia. In: Historyja handliu ŭ Bielarusi (ad staražytnaha času da kanca XX st): prabliemy vyvučennia i pierspiektyvy dasliedavannia : materyjaly I Mižnarodnaj navukova-praktyčnaj kanfierencyi. Minsk: Technalohija; 2014. pp. 130–149.](https://www.zotero.org/google-docs/?yfeJGw)

[116. Plavinski MA. Kurgan 5 mogil’nika Pogoshcha kak primer elitarnogo pogrebal’nogo kompleksa kontsa KH — nachala XI v. na severo-zapade Polotskoy zemli. In: Drevnosti Issledovaniya Problemy. Kishinev-Tiraspol: Izdatel’skiy dom Stratum; 2018. pp. 445–464.](https://www.zotero.org/google-docs/?yfeJGw)

[117. Dučyc LU. Kurgany s baltskimi ukrasheniyami na territorii Belarusi. Liet Archeol. 2001;21: 235–240.](https://www.zotero.org/google-docs/?yfeJGw)

[118. Duk DV, Kots AL. Staražytny Polack: novyja danyja pa vynikach raskopak na terytoryi Nižniaha zamka ŭ 2014 hodzie. Materyjaly Pa Arch Bielarusi. 2016;27: 31–41.](https://www.zotero.org/google-docs/?yfeJGw)

[119. Klimau MV. Novaje adkryccio ŭ chranalohii staražytnaha Polacka. Byelaruski Gistar Chasopis. 2010;5: 5–7.](https://www.zotero.org/google-docs/?yfeJGw)

[120. Dučyc LU. Spravazdača ab archiealahičnych raskopkach u Hlybockim rajonie i archiealahičnych razviedkach u Pastaŭskim rajonie Viciebskaj voblasci ŭ 1992 hodzie. National Academy of Sciences of Belarus; 1992 p. 27. Report No.: 1424. Rys. 2. Kurhan №1. Skrabianiec; p. 11.](https://www.zotero.org/google-docs/?yfeJGw)

[121. Dučic LV. Otchet o polevykh issledovaniyakh v 1989 godu na territorii Glubokskogo i Dokshitskogo rayonov Vitebskoy oblasti. Minsk: National Academy of Sciences of Belarus; 1989 p. 62. Report No.: 1130. Ris. 21. Zhalʹnik №1 u d. Perevoz Glubokskogo r-na (ryadom s kurg. mogilʹnikom №4); p. 36.](https://www.zotero.org/google-docs/?yfeJGw)

[122. Charauko VU. Archiealahičnyja dasliedavanni na kurhanna-žaĺničnym mohiĺniku kalia v. Biruli Dokšyckaha rajona ŭ 2011 h. Materyjaly navukovych kanfierencyj. Minsk: Nacyjanaĺnaja biblijateka Bielarusi; 2013. pp. 24–25. Mal. 1. Mohiĺnik kalia v. Biruli Dokšyckaha r-na. Kamiennyja kanstrukcyi (sumiaščennie planaŭ nadmahiĺnych i ŭnutrymahiĺnych kanstrukcyj); p. 24.](https://www.zotero.org/google-docs/?yfeJGw)

[123. Charauko VU. Archiealahičnyja dasliedavanni na kurhanna-žaĺničnym mohiĺniku kalia v. Biruli Dokšyckaha rajona ŭ 2011 h. Materyjaly navukovych kanfierencyj. Minsk: Nacyjanaĺnaja biblijateka Bielarusi; 2013. pp. 24–25. Mal. 2. Mohiĺnik kalia v. Biruli Dokšyckaha r-na. Plan razmiaščennia pachavanniaŭ; p.24.](https://www.zotero.org/google-docs/?yfeJGw)

[124. Dučic LV. Otchet o polevykh issledovaniyakh v 1989 godu na territorii Glubokskogo i Dokshitskogo rayonov Vitebskoy oblasti. Minsk: National Academy of Sciences of Belarus; 1989 p. 62. Report No.: 1130. Ris. 11. Situatsionnyy plan kurgannogo mogilʹnika №4 u d. Perevoz Glubokskogo rayona; p. 26.](https://www.zotero.org/google-docs/?yfeJGw)
