# Supplementary material for: Medieval and early modern diets in the Polack region of Belarus: A stable isotope perspective
Source: PLoS One. 2022 Oct 7;17(10):e0275758. doi: 10.1371/journal.pone.0275758 (PMC9543997; doi:10.1371/journal.pone.0275758)
Supplement: Attachment — Submitted filename: PONE-D-22-08905_reviewer - with comments.pdf [file pone.0275758.s002.pdf]

PLOS ONE

Medieval and Early Modern diets in the Polack region of Belarus: A stable isotope perspective

--Manuscript Draft--

**Manuscript Number:**	PONE-D-22-08905
**Article Type:**	Research Article
**Full Title:**	Medieval and Early Modern diets in the Polack region of Belarus: A stable isotope perspective
**Short Title:**	Medieval and Early Modern diets in the Polack region of Belarus
**Corresponding Author:**	Vera Haponava, MA<br>University of Warsaw: Uniwersytet Warszawski<br>Warsaw, POLAND
**Keywords:**	Palaeodiet; subsistence; stable isotope analysis; collagen; carbon; nitrogen; Belarus; the Grand Duchy of Lithuania; the Polish-Lithuanian Commonwealth; Polack; Medieval; Early Modern; urban; rural; elite; commoner; peasant; fish consumption; manuring; animal protein; plant protein
**Abstract:**	In western and north-western Europe there has been a growing focus on exploring how major economic, political, and social changes during the Medieval period impacted the lived experience of different populations and sectors of society. Stable isotope analysis has proven particularly powerful in this regard, providing direct insights into the long-term diets of individuals and communities. Despite experiencing similarly dramatic social reconfigurations and changes, eastern Europe has, however, received far less attention in this regard. The territory of Belarus has, especially, so far remained a relative blank spot on the bioarchaeological map of Europe, though cities such as Polack emerged rapidly as key nodes within a growing economic and religious network. To gain direct insight into the diets of inhabitants of the Polack region of Belarus in the 11-18 th centuries, we applied stable carbon and nitrogen isotope analysis to bone and dentine collagen from human (n=143) and animal individuals (n=105) from the city of Polack and surrounding rural sites. Results indicate a diet based on C_3 terrestrial resources, which did not differ between sexes and showed limited variation over time. Contrary to expectations, it appears that animal products were commonly consumed by peasants, but no significant reliance on fish resources or millet consumption is found. In contrast to examples from western Europe, we argue that the diets in the city and the surrounding villages remained broadly similar for the majority of the population, and similar to commoners analysed in Poland and Lithuania, perhaps suggestive of slightly different economic changes operating in this part of the Medieval world.
**Order of Authors:**	Vera Haponava, MA
	Aliaksei Kots
	Mary Lucas
	Max Both
	Patrick Roberts
**Additional Information:**	
**Question**	**Response**
**Financial Disclosure**<br><br>Enter a financial disclosure statement that describes the sources of funding for the work included in this submission. Review the submission guidelines for detailed requirements. View published research	VH, ML, and PR thank the Max Planck Society (https://www.shh.mpg.de/en) for funding the laboratory work and stable isotope analysis. VH received an additional funding support from the Faculty of Archaeology of the University of Warsaw (https://www.archeologia.uw.edu.pl/en/new-main-page/) in the amount of 2801 PLN towards the cost of trip to Germany for the processing of samples. The funders had no role in study design, data collection and analysis, decision to publish, or preparation of the manuscript.

Powered by Editorial Manager® and ProduXion Manager® from Aries Systems Corporation

articles from *PLOS ONE* for specific examples.<br><br>This statement is required for submission and **will appear in the published article** if the submission is accepted. Please make sure it is accurate.<br><br>**Unfunded studies**<br>Enter: *The author(s) received no specific funding for this work.*<br><br>**Funded studies**<br>Enter a statement with the following details:<br>• Initials of the authors who received each award<br>• Grant numbers awarded to each author<br>• The full name of each funder<br>• URL of each funder website<br>• Did the sponsors or funders play any role in the study design, data collection and analysis, decision to publish, or preparation of the manuscript?<br>• **NO** - Include this sentence at the end of your statement: *The funders had no role in study design, data collection and analysis, decision to publish, or preparation of the manuscript.*<br>• **YES** - Specify the role(s) played.<br><br>* typeset	
**Competing Interests**<br><br>Use the instructions below to enter a competing interest statement for this submission. On behalf of all authors, disclose any competing interests that could be perceived to bias this work—acknowledging all financial support and any other relevant financial or non-financial competing interests.<br><br>This statement is required for submission and **will appear in the published article** if the submission is accepted. Please make sure it is accurate and that any funding sources listed in your Funding Information later in the submission form are also declared in your Financial Disclosure statement.	The authors have declared that no competing interests exist.

View published research articles from *PLOS ONE* for specific examples.<br><br>**NO authors have competing interests**<br><br>Enter: *The authors have declared that no competing interests exist.*<br><br>**Authors with competing interests**<br><br>Enter competing interest details beginning with this statement:<br><br>*I have read the journal's policy and the authors of this manuscript have the following competing interests: [insert competing interests here]*<br><br>* typeset	
**Ethics Statement**<br><br>Enter an ethics statement for this submission. This statement is required if the study involved:<br><br>• Human participants<br>• Human specimens or tissue<br>• Vertebrate animals or cephalopods<br>• Vertebrate embryos or tissues<br>• Field research<br><br>Write "N/A" if the submission does not require an ethics statement.<br><br>General guidance is provided below. Consult the submission guidelines for detailed instructions. **Make sure that all information entered here is included in the Methods section of the manuscript.**	N/A

Powered by Editorial Manager® and ProduXion Manager® from Aries Systems Corporation

**Format for specific study types**<br><br>**Human Subject Research (involving human participants and/or tissue)**<br>• Give the name of the institutional review board or ethics committee that approved the study<br>• Include the approval number and/or a statement indicating approval of this research<br>• Indicate the form of consent obtained (written/oral) or the reason that consent was not obtained (e.g. the data were analyzed anonymously)<br><br>**Animal Research (involving vertebrate animals, embryos or tissues)**<br>• Provide the name of the Institutional Animal Care and Use Committee (IACUC) or other relevant ethics board that reviewed the study protocol, and indicate whether they approved this research or granted a formal waiver of ethical approval<br>• Include an approval number if one was obtained<br>• If the study involved *non-human primates*, add *additional details* about animal welfare and steps taken to ameliorate suffering<br>• If anesthesia, euthanasia, or any kind of animal sacrifice is part of the study, include briefly which substances and/or methods were applied<br><br>**Field Research**<br><br>Include the following details if this study involves the collection of plant, animal, or other materials from a natural setting:<br>• Field permit number<br>• Name of the institution or relevant body that granted permission	
**Data Availability**<br><br>Authors are required to make all data underlying the findings described fully available, without restriction, and from the time of publication. PLOS allows rare exceptions to address legal and ethical concerns. See the PLOS Data Policy and FAQ for detailed information.	Yes - all data are fully available without restriction

A Data Availability Statement describing where the data can be found is required at submission. Your answers to this question constitute the Data Availability Statement and **will be published in the article**, if accepted.<br><br>**Important:** Stating 'data available on request from the author' is not sufficient. If your data are only available upon request, select 'No' for the first question and explain your exceptional situation in the text box.<br><br>Do the authors confirm that all data underlying the findings described in their manuscript are fully available without restriction?	
**Describe where the data may be found in full sentences. If you are copying our sample text, replace any instances of XXX with the appropriate details.**<br><br>• If the data are **held or will be held in a public repository**, include URLs, accession numbers or DOIs. If this information will only be available after acceptance, indicate this by ticking the box below. For example: *All XXX files are available from the XXX database (accession number(s) XXX, XXX.).*<br>• If the data are all contained **within the manuscript and/or Supporting Information files**, enter the following: *All relevant data are within the manuscript and its Supporting Information files.*<br>• If neither of these applies but you are able to provide **details of access elsewhere**, with or without limitations, please do so. For example:<br><br>*Data cannot be shared publicly because of [XXX]. Data are available from the XXX Institutional Data Access / Ethics Committee (contact via XXX) for researchers who meet the criteria for access to confidential data.*<br><br>*The data underlying the results presented in the study are available from (include the name of the third party*	All relevant data are within the manuscript and its Supporting Information files

and contact information or URL).<br>• This text is appropriate if the data are owned by a third party and authors do not have permission to share the data.<br><br>* typeset	
Additional data availability information:	

Powered by Editorial Manager® and ProduXion Manager® from Aries Systems Corporation

Manuscript text
Click here to access/download;Manuscript;Haponava et al. - Manuscript.docx

Medieval and Early Modern diets in the Polack region of Belarus: A stable isotope perspective

Vera Haponava[1]*, Aliaksei Kots[2], Mary Lucas[3], Max Both[4], Patrick Roberts[3,5]*

[1] Faculty of Archaeology, University of Warsaw, Warsaw, Poland

[2] Department of History and Tourism, Polotsk State University, Novopolotsk, Belarus

[3] Department of Archaeology, Max Planck Institute for the Science of Human History, Jena, Germany

[4] Institute of Prehistoric Archaeology, Freie Universitaet, Berlin

[5] School of Social Sciences, University of Queensland, St Lucia, Brisbane, Australia

* Corresponding author

E-mail: harti@protonmail.com

roberts@shh.mpg.de

Abstract

In western and north-western Europe there has been a growing focus on exploring how major economic, political, and social changes during the Medieval period impacted the lived experience of different populations and sectors of society. Stable isotope analysis has proven particularly powerful in this regard, providing direct insights into the long-term diets of individuals and communities. Despite experiencing similarly dramatic social reconfigurations and changes, eastern Europe has, however, received far less attention in this regard. The territory of Belarus has, especially, so far remained a relative blank spot on the bioarchaeological map of Europe, though cities such as Polack emerged rapidly as key nodes within a growing economic and religious network. To gain direct insight into the diets of inhabitants of the Polack region of Belarus in the 11-18th centuries, we applied stable carbon and nitrogen isotope analysis to bone and dentine collagen from human (n=143) and animal individuals (n=105) from the city of Polack and surrounding rural sites. Results indicate a diet based on C_3 terrestrial resources, which did not differ between sexes and showed limited variation over time. Contrary to expectations, it appears that animal products were commonly consumed by peasants, but no significant reliance on fish resources or millet consumption is found. In contrast to examples from western Europe, we argue that the diets in the city and the surrounding villages remained broadly similar for the majority of the population, and similar to commoners analysed in Poland and Lithuania, perhaps suggestive of slightly different economic changes operating in this part of the Medieval world.

1.0 Introduction

The Medieval period of Europe witnessed a series of major economic, political, and social changes, including the rise of Christian states, the expansion of urban networks together with facilitated disease transmission, the intensification of hierarchies and wealth inequality, and increased access to non-local resources [1-4]. While these broader processes are well-documented historically and archaeologically, their impacts on local populations in different parts of Europe are often more obscure, particularly given that archival records are often biased towards elite communities [1,4,5]. Meanwhile, eastern Europe, located beyond the geographic scope of Roman Christianization, has also received less attention and is often considered to be poorer, less developed, and less "European" [5]. ~~This is despite the fact that~~ eastern Europe also went through dramatic social changes, with the territory of Belarus in particular witnessing the appearance of its first clear cities and states at the end of the first millennium and the beginning of the second millennium AD, the rise and decline of feudal relationships between the 9th and the beginning of the 19th centuries AD, a number of major military conflicts and constant raids, the formation of land relations (e.g. adoption of the the so-called Voloka Decree), and the appearance of Magdeburg Law in the cities [6-10]. Being part of the Grand Duchy of Lithuania, at its height the largest state in Medieval Europe, and described in the literature as a melting pot of ethnoses, religions and cultures [11], the region thus remains a yet untapped opportunity to explore issues pertaining to identity, equality, human agency, resilience and adaptation to socio-political transitions in the Medieval world.

The study of individual and group diets offers the possibility of exploring the interaction of cultural, political, and economic change in the lived experience of Medieval populations. Various methodologies can be applied to study past human diets. However, it is in combination where they provide the most comprehensive information. While providing high levels of detail on cuisine and foodstuffs consumed, written records are still virtually nonexistent for the Early Medieval period in certain parts of Europe [12,13] and, where available, historical documentation revolves around social and religious elite groups [5]. Zooarchaeology and archaeobotany can provide rich 'snapshots' into moments of food consumption [13]. However, they are limited by taphonomic biases [14] and retrieval techniques and are largely incapable of showing how available resources were distributed within the population through time [3]. Stable isotope analysis of human remains has emerged as a means of obtaining direct and quantitative information about the diet of individuals and communities [12-14]. It can also, when combined with other more traditional methodologies, enable the exploration of the diet of individuals overlooked in the Medieval historical record, such as juveniles, individuals from lower social statuses, and those residing in nonurban locations [5].

~~Expanding~~ isotopic studies in western and north-western Europe have demonstrated the role of religious prescription, status, and political upheaval on diets and economies [3,4,15,16]. However, this technique has been rarely applied in an eastern European Medieval context [5], despite its potential to answer similar, albeit more region-specific, questions. In this study we apply stable carbon and nitrogen isotope analysis to a large sample of peasants from 15 rural graveyards and the inhabitants of one major city from the northern part of Belarus, as well as animal

remains retrieved from several contemporary sites in the region. The time span of the sites covers the period from the 11th till the 18th centuries AD, and is subdivided into three roughly equal periods, corresponding to periods of major state formation within the territory - Ancient Rus principalities (9th century AD), the Grand Duchy of Lithuania (13th century ASD) and the Polish-Lithuanian Commonwealth (16th century AD). Our goal is to determine whether there were temporal dietary changes brought by the associated political, economic, and social transition, both in urban and rural settings, in male and female individuals, and between 'commoners' versus elites. We also seek to compare our data to that available from the neighbouring regions of Poland and Lithuania to explore how diets varied across the expanding states of the region.

2.0 Background

2. 1 Diet in 11-18th-century Belarus based on historical, archaeological and ethnographic sources

The second millennium AD witnessed dramatic social, political, and cultural changes within the territory of the current nation of Belarus, including the appearance of a clear, established urban network. Formed on the trans-European trade route "from the Varangians to the Greeks", the city of Polack (also Polatsk, Polotsk; official transliteration from the Belarusian language is used here for Belarusian toponyms and names, spelled with Belarusian Latin alphabet) was the major trade, political and

cultural centre of the region [7,9]. Polack Principality was the most prominent of the proto-states on the Belarusian lands, having its own ruling dynasty and maintaining certain independence even when it became part of larger territorial formations in this part of Eurasia [9,17]. Over the course of the period between the 11th and 18th centuries, these formations included Ancient Rus, the Grand Duchy of Lithuania (GDL), and the Polish-Lithuanian Commonwealth (PLC). The latter state ceased to exist at end of the 18th century and the Polack region, as well as other Belarusian lands, was gradually annexed by the Russian Empire. As social identity is believed to be deeply embedded in diet and consumption behaviours, these events might have been paralleled by dietary changes. So far the studies related to nutrition in Belarus have focused on written sources [18,19], ethnohistoric and archival data [20-22] and archaeological materials such as animal remains [23] and charred grain finds [24,25]. These shed light on the major food products and the ways of their preparation through the discussion of agriculture, husbandry and hunting, trade, or the menu of nobility.

Based on these studies we may assume that throughout the 11-18th centuries a plant-based diet dominated across the territory of Belarus, with grains constituting the base of the local inhabitants' subsistence [10,20,26]. Rye bread was the main food product for centuries, as well as gruel [7,22,27]. Wheat, barley and oats followed rye in popularity [10,24,25,28], along with garden crops like cabbage and turnip [7,20,28-30]. By contrast, millets appear in rather negligible quantities among charred grain finds and continue to decrease in number over the period of our focus [24], though some authors name millets as a widely grown crop and even one of the main foods in Ancient Rus [7,28]. It is similarly hard to estimate the role of legumes, but overall they seem to appear in rather small quantities as well [24,25]. Manuring is considered a

necessary component of the crop rotation system, which was the dominant agricultural system throughout the period of our study [7,10,25,28,30]. However, based on written and archaeological sources there is a possibility that manuring was not used or had limited application in northern Belarusian territories between the 12-18th centuries [10,31].

The role of husbandry in the economy was apparently secondary throughout the 11-18th centuries, though it still provided vital subsistence components like meat and milk products [7,20,28]. Fat, milk, butter or oil were common additives to non-meat dishes like porridge, bread, and cabbage, and peasants have been argued to have eaten meat only rarely, mostly during festive dinners [21,27]. Pig was the dominant domestic animal, followed by cattle [23,32]. Hunting, fishing, bortfreat (collection of wild honey), and gathering of mushrooms, berries, nuts and medicinal herbs occupied an important place in the ancient peasant economy, providing additional sources of food [7,20,28]. At the same time, the role of hunting was marginal [23] - only for the feudal aristocracy did hunting remain an important social activity [7]. The territory surrounding Polack, Paazerje, is rich in water resources [33], and fishing was a widespread occupation [7,34]. Commonly caught species included perch, bream, tench, roach, ide, pike, catfish, sea and river lamprey, trout, carp, eel, and pike perch (zander) [7,33,35]. Christian fasts lasted in sum almost half a year [5,20,36], thus a substantial influence of the Christian religion on fish consumption may be expected for the whole period of our study. Fast was reportedly kept very strictly, even by breastfeeding women and by children starting from 7 years of age [27].

Based on the available information it appears that commoners living in towns would have had diets similar to those of rural peasants. Despite agricultural activity

and husbandry being common in cities including Polack in the 10-16th centuries, city inhabitants were the main consumers of village production, increasingly obtaining their food from rural neighbourhoods [7,9,24,25,28,31,35]. The same was likely true for the later period of the 16-18th centuries [37]. One possible difference between the diets of peasants and urban dwellers could be that foreign goods, including food products, almost never reached rural areas [7], while Polack citizens already obtained some imported foods and drink from the very beginning of the period of our focus [9]. However, any imports of foodstuffs seem to have focused on rather luxurious products, such as wine, oil, salt, spices, fruits, and salted herring [7,9,38-40]. Therefore, if differences based on access to rare foreign foods existed between peasants and citizens, they were likely rather marginal and more applicable to rich citizens than commoners. The nutrition of the elites from the times of Ancient Rus has been argued to be more abundant overall and richer in meat in particular [41]. On the other hand, Navahrodski [42] proposes that, until the 16th century, social elites in Belarusian lands consumed almost the same products as the commoners, maybe only of better quality and more regularly. From the 16th century, the cuisine of the nobility was influenced by Poland, for which excessive fat content, presence of sweet dishes, a lot of spices and salt, and consumption in great quantities were typical, testifying the high status of an individual. Additionally, with time, nobility and rich citizens started to use more and more imported products [42] like citrus fruits, potatoes (later), foreign fish, caviar, and even snails. Wine was also imported in large quantities [18]. Overall, historical and archaeological evidence indicates that diet variability and access to nonlocal resources increased throughout Europe during the Medieval and Early Modern periods [5].

2.2. Stable isotope analysis and palaeodiet in Medieval eastern Europe

The use of stable isotope data in palaeodietary studies is based on the fact that stable isotopic ratios of carbon and nitrogen in the body reflect those of the food consumed during life [3,12]. Stable carbon isotope values of bone collagen (expressed as $\delta^{13}C$ (‰)) have traditionally been used to distinguish between dietary protein originating from plants (and animals consuming these plants) utilising the C_4 photosynthetic pathway (e.g., maize and millet) versus C_3 plants (most temperate species, e.g. wheat and barley). It is also used to distinguish between marine and terrestrial protein consumption, with marine environments having a different source of CO_2 that leads to an overlap with C_4 resources [3,12-14,36]. In Europe, there are only rare examples of wild C_4 plants, primarily found in the Mediterranean, plus domestic species which have been introduced at various points, the most important of which are the millets [14]. In the case of bone collagen, it is worth noting that the $\delta^{13}C$ signal will be primarily reflective of protein contributions to the diet. This means that high protein (e.g. meat, aquatic) resources can swamp the signal at the expense of lower protein (e.g. crop) resources [43]. There is an approximate 1‰ increase in $\delta^{13}C$ with each trophic level [44].

Stable nitrogen isotope analysis of bone collagen ($\delta^{15}N$) provides additional resolution of the trophic position of an individual within the foodchain. In archaeological contexts, this includes testing the proportional consumption of animal (meat or dairy) versus plant protein or aquatic versus terrestrial protein, as both freshwater and marine ecosystems tend to, ~~on average,~~ have longer foodchains

resulting in higher $\delta^{15}N$ values (although freshwater settings have much more variable $\delta^{13}C$ than marine ecosystems) [3,12-14,36]. Not all variation in stable isotope ratios is directly attributable to differences in foods consumed among individuals or populations, however [3]. Other factors that may affect the $\delta^{13}C$ and $\delta^{15}N$ values of food sources for agricultural human populations, and their domesticates, include physiology (starvation, nursing), environmental factors such as aridity, farming practices such as manuring which leads to ^{15}N enrichment of soils and thus plants, the "canopy effect" which leads to lower $\delta^{13}C$ values in ~~wild~~ animals occupying closed forest environments compared to d~~omesticated~~ animals occupying open areas, and variation in herding and grazing practices for different livestock taxa [13,36].

Although there is currently a complete absence of isotope-based studies dedicated to palaeodiet on the territory of Belarus, we can turn to those conducted in the neighbouring regions as a reference. For example, a series of isotope studies published by Reitsema, Kozłowski and others [2,3,45] investigated palaeodiet in Poland for the period ranging from the times of the Wielbark population of the 2nd century AD to 10-18th centuries' Medieval Poland and the Polish-Lithuanian Commonwealth. They found dramatic "status-based" differences in diet in the Commonwealth, with much fish consumed by elites, and detected certain trends like the lack of consistent diet changes over time and presence of site-specific changes. In Lithuania, research by Skipitytė et al. [36] and Whitmore et al. [5] showed a certain contradiction to the historical sources and unexpected results in terms of low fish consumption. Stable isotopes were also used for the examination of palaeodiet in the 13th-century city of Yaroslavl, Russia, where a substantial proportion of animal protein in the diet was observed [46].

3.0 Materials and Methods

3.1.1 Sites and burials

The human remains analysed in this study originate from burial sites that include kurgan and ground cemeteries in rural and urban environments. Early ground cemeteries partly contemporaneous to kurgans are characterised by stone constructions in the form of a pavement or lining, often with larger stones at the head and/or foot of the burial [47-49]. Such 'stone graves' are mostly dated to the 13-14th centuries AD, though they existed until the 18th century [48]. Kurgan and ground cemeteries frequently form a long-used burial complex together in the region. A large concentration of such burial complexes is found in the south of the Belarusian Dzvina (also Dvina, Western Dvina, or Daugava) basin - the territory of our study. During most of the period of our focus, in the 14-18th centuries AD, the deceased were interred without any grave goods, including crosses [47,49]. Rural cemeteries that provide the sample of peasants in this study include Biruli, Domžarycy, Doŭhaje, Dubraŭka, Dzmitraŭščyna, Ivieś, Kazloŭcy, Klieščyno, Michalinava, Padsvillie, Pieravoz-4, Sielišča, Skrabianiec, Vaŭča and Ziabki. Fig. 1 shows a map of the sampled sites grouped into the three periods (11-13th, 13-16th, 16-18th centuries AD) of major state formation mentioned above (Ancient Rus, the GDL, the PLC).

Fig 1. *Map of the sites from which human and animal materials were sampled, colour-coded by their dating. The red star shows the urban centre of Polack while all other sites are designated as 'rural'.*

Kurgan cemeteries near the villages of Domžarycy, Dzmitraŭščyna, Sielišča, Skrabianiec, Ziabki and the modern town of Padsvillie are dated to the 10-12th centuries AD [50-53]. A ground cemetery with cremations and inhumations near the village of Kazloŭcy is also dated to the 10-12th centuries AD [54], and Plavinski [55] believes it was actually a kurgan cemetery. The ground cemetery of Ivieś functioned throughout the 14-18th centuries AD, with most burials dated to the 14-16th centuries AD [49]. Klieščyno is dated to the 16-18th centuries [56] and in the Michalinava 'stone graveyard' the studied burials belong to the 18-19th centuries AD, although the functioning of the cemetery began earlier and continued throughout the 15-19th centuries [57]. In Dubraŭka the excavated part of the cemetery functioned over the 17-18th centuries AD [58] and in Doŭhaje over the 14-18th centuries [59]. However, as most of the burials are believed to belong to the earlier part of this broad dated phase, the 14-16th centuries, the Doŭhaje sample is considered to fall within the period of the 13-16th centuries AD in the present work.

In several sites in this study, the sampled material comes from two phases. Namely, these are the kurgan and later ground burials of Biruli (11-12th centuries AD and the 14th - beginning of the 16th century AD) [60,61], the site of Pieravoz (kurgan burials are dated to the 11th - beginning of the 12th century AD, while the ground part of cemetery is dated to the 13-14th centuries AD) [51] and Vaŭča (kurgans dated to the 12-14th centuries AD, while 'stone graves' date to the 13-14th centuries AD) [62,63]. In

fact, Doŭhaje (a ground cemetery of the 14-16th centuries AD) and Ziabki (10-12th centuries AD kurhans) are located so close that their burials represent another long-functioning common complex. Thus, the rural sample covers the whole timespan of focus of this study, with the groups from each period being roughly similar in number (see Table 1 for the dating, provenance, and number of individuals sampled).

Table 1. Summary of cemetery sites from which human material originated.

Site name	Cemetery type	Dating, cc AD	# individuals sampled
Biruli	Kurgan	11-12	1
	Ground	14-16	9
Domžarycy	Kurgan	11	4
Doŭhaje	Ground	14-16	5
Dubraŭka	Ground	17-18	6
Dzmitraŭščyna	Kurgan	11	1
Ivieś	Kurgan	12	1
	Ground	14-16	10
Kazloŭcy	Kurgan?	10-12	2
Klieščyno	Ground	16-18	3
Michalinava	Ground	18-19	11
Padsvillie	Kurgan	12	2
Pieravoz-4	Kurgan	11-12	3
	Ground	13-14	7
Sielišča	Kurgan	10-12	2
Skrabianiec	Kurgan	11	3

Vaŭča	Kurgan	12-14	2
	Ground	13-14	7
Ziabki	Kurgan	11-12	2
Polack - Lower Castle	Ground	13-14	18
	Ground	18	10
Polack - Township	Ground	17-18	13
Polack - Upper Castle	Ground	11-13	21
	Total from the period of the **11-13th** centuries:		**44**
	Total from the period of the **13-16th** centuries:		**56**
	Total from the period of the **16-18th** centuries:		**43**

The city of Polack

The city of Polack played the role of the administrative, social and economic center of the region under study in the 11-16th centuries AD [33]. The human and animal material comes from several parts of this city - the Lower Castle, Upper Castle, and Township. The skeletal material from these sites represents the urban sample in this study and, like the rural sample, quite evenly covers the whole timespan of the 11-18th centuries (Table 1).

A multi-layered graveyard was excavated in the territory of the Lower Castle, yielding individuals from burials near an Orthodox church dated to the 13-14th centuries AD and individuals dated to the 17th century AD - mostly young males interpreted as having military affiliations based on finds of bullets with some of the deceased [64]. The territory of the ancient Township turned into a cemetery in the 17-18th centuries AD, which was excavated in 2007 and 2009 and is interpreted as being

Catholic from the scarce inventory of burial goods uncovered. Based on the Catholic confession, and on the fact that the cemetery is located in the center of the city, the researchers concluded that the deceased belonged to Polack nobility [65] - thus this group constitutes the elite sub-sample which will be compared to ordinary individuals in our study. The ground necropolis on the territory of the Upper Castle was discovered in 2020, and by autumn 2021 more than 100 burials dated to the 11-13th centuries AD had been investigated.

3.1.2 Faunal remains

Due to the fact that the exact isotopic ratios at the base of the food chain can vary across space and time as a product of various environmental and economic parameters it is necessary to sample contemporaneous fauna to fully understand and interpret human results [12]. In this study samples of animal remains originate again from the city of Polack (Lower Castle and Upper Castle), the settlement site and one of the kurgans of Biruli, and several more sites associated with the Polack Principality territory - Lučna (15-17th centuries AD) and Čarscviady (11-12th centuries AD), rural sites that hosted nobility residences, and the Mienka township (11-12th centuries AD) (Table 2). Fauna was identified by Dr. Urszula Iwaszczuk and Vera Haponava using the reference collection at the Institute of Mediterranean and Oriental Cultures of the Polish Academy of Sciences and a standard zoological reference work [66,67].

Table 2. Summary of sites from which animal material originated.

Site name	Dating, cc AD	# individuals sampled

Biruli	10-11	12
	11-13	1
Čarscviady 3	11-12	13
Lučna	15-16	14
	16-17	2
Mienka	10-11	23
Polack - Lower Castle	10-11	13
	11-13	3
	13-14(?)	5
	14-15	3
	17-18	3
	18-19	5
Polack - Upper Castle	16	3
	17	1
	18	1
	17-18	1
	18-19	2
	Total from the period of the **11-13th** centuries:	**65**
	Total from the period of the **13-16th** centuries:	**25**
	Total from the period of the **16-18th** centuries:	**15**

3.1.3 Samples

A total of 100 human bones and 43 human teeth were sampled in this study. The individuals and the elements sampled can be seen in Table S1 (Supplementary

Information). Human bone collagen was extracted primarily from rib bones (92/100). Where postcranial elements were not available in the collection, permanent maxillary or mandibular third or second molars were preferentially sampled for dentine collagen to reflect the adolescent diet of the individuals. When pathological conditions were expressed on the bones or teeth (e.g. carious lesions), non-affected elements were prioritised for sampling. Given the heterogeneity of samples, the results should be interpreted with caution, as different periods of life are compared here: ribs represent approximately last five years of life [68], while dentine from the roots that were cut for analysis in this study represent the ages of 14-20 in the case of the third molars and 8-14 in the case of second molars [69].

For faunal remains, bones (e.g. ribs) (80/105) or teeth developing later in life (such as M3, M2 or premolars in some species) were preferentially selected for collagen extraction (see Table S2, Supplementary Information). To ensure that samples did not come from the same individual, bones and teeth of the same species from different archaeological contexts, unique elements of the same species from the same archaeological context, or elements clearly belonging to different individuals (e.g. of different age-at-death) were used. The permits for the use of samples were obtained for the described study from the institutions holding them in collections (Polotsk State University and the Institute of History of the National Academy of Sciences of Belarus).

Sex determination of human individuals was based on pelvic and cranial morphology [70]. Only adult individuals were included in the study with the exception of 12 adolescents (i.e. only individuals with the second molar fully formed

and erupted). The age-at-death was assessed observing cranial suture closure following Piontek [71] and epiphyseal fusion for younger individuals based on Herrmann et al. [72]. Biological profiles for humans from the collection of the Polack State University were assessed by V.A. Yemialyanchyk and D.S. Gritskevich.

In the collection of the Institute of History of the National Academy of Sciences, postcranial skeletal elements were not available. Age was estimated using dental wear patterns [73] coupled with analysis of the sphenooccipital synchondrosis fusion [74]. Sex assessment was based on cranial morphology [70]. In the cases where sex and age estimation based on the study of the cranium contradicted those stated in the available publication or archaeological report, the latter assessments were used based on the assumption that it could have been estimated with more certainty at the time of analysis when postcranial elements were also available.

3.2.1 Stable isotope measurement

The standard modified-Longin procedure described in Richards and Hedges [75] was used for collagen extraction. First, the surface of the bone and tooth samples was superficially cleaned through gentle sand ablation. 1 g samples of bone and tooth roots were obtained by use of a diamond cutting blade. Approximately 1 g of cleaned bone or tooth root was then demineralized in 10 mL aliquots of 0.5 M HCl. The acid was changed every 48 hours for 5-17 days until demineralization was achieved. Then, the demineralized sample was washed three times in Milli-Q® distilled water and gelatinized using pH 3 HCl at 70 °C for 48 h. The resulting solution was filtered into a test tube using an EZEE filter and frozen for 24 hours. The frozen sample was then

lyophilized in a freeze dryer until fully dried. 1 mg aliquots of dried collagen were weighed into tin capsules for analysis.

Samples were combusted in a Thermo Scientific Flash 2000 Elemental Analyser coupled to a Thermo Delta V Advantage Mass Spectrometer at the Isotope Laboratory, Max Planck Institute for the Science of Human History (Jena, Germany). All isotopic measurements refer to the ratio between heavy and light isotope ($^{13}C/^{12}C$ or $^{15}N/^{14}N$) measured as δ values in parts per mil (‰) calibrated using a two-point calibration between a series of International Standards (IAEA-N-2 Ammonium Sulfate: $\delta^{15}N$ = +20.3 ± 0.2‰, USGS40 L-Glutamic Acid: $\delta^{13}C$ = −26.389 ± 0.042‰, $\delta^{15}N$ = −4.5 ± 0.1‰, IAEA-CH-6 Sucrose: $\delta^{13}C$ = −10.449 ± 0.03‰) and in-house laboratory standards (fish gelatin: $\delta^{13}C$ = −15.7 ± 0.1‰, $\delta^{15}N$ = −14.3 ± 0.1‰; Urea: $\delta^{13}C$ = −41.30 ± 0.04‰, $\delta^{15}N$ = −0.32 ± 0.2‰). All samples were measured in duplicate. Analytical error was studied through the repeated measurement of an in-house fish gelatin standard (-15.7 ± 0.3‰ for $\delta^{13}C$ and 14.3 ± 0.2‰ for $\delta^{15}N$).

3.2.2 Statistical analysis

Statistical analyses were conducted in STATISTICA 14. A Shapiro-Wilk test was performed to test if the human and animal bone collagen $\delta^{13}C$ and $\delta^{15}N$ data within the analysed groups were normally distributed. Levene's test was performed to check for homogeneity of variances. Parametric data was then analysed using Student’s two-sample t-tests for independent samples and one-way ANOVA tests with post-hoc Bonferroni Tests for multiple pairwise comparisons. When the isotopic data were found to follow a non-normal distribution, non-parametric Mann-Whitney U and

Kruskal–Wallis tests were employed, with subsequent Mann-Whitney U tests for pairwise comparisons in the latter case. To determine if a linear relationship exists between $\delta^{13}C$ and $\delta^{15}N$ values of bone collagen linear regression models were used. In all cases, the results were considered significant if the p-value was lower than 0.05. Means are reported ± 2 standard deviations in the below text.

4.0 Results

To ensure that samples were sufficiently preserved and free from diagenetic contamination, the atomic carbon to nitrogen ratio (C:N) was used as a primary criterion according to the parameters published by van Klinken [76]. Collagen yields in the whole sample ranged from 0.002 to 26.36%. Three animal samples were excluded from the analysis as their C:N ratio was outside of the acceptable range of 2.9-3.6 (Bir13-N5-6.02, Bir14-N1A.01, and PUC16-NV2.3.03). One further animal sample which had a yield below 1% (Bir13-N2.01) was also abandoned even though its C:N ratio was within the acceptable range of 2.9-3.6. Therefore, the final number of samples used in the analyses was 244 out of 248 (Table S1).

4.1 Faunal remains

As shown in Fig. 2, $\delta^{13}C$ and $\delta^{15}N$ measurements for domestic herbivores (n = 49) range from −25.4‰ to −20.4‰ (mean −22.8‰ ± 2.1) and from 4.7‰ to 10.4‰ (mean 6.6‰ ± 2.2), respectively. Wild species generally plot lower than the domestic herbivores on the $\delta^{15}N$ axis and cluster more tightly in terms of $\delta^{13}C$. For the wild herbivores the

values are −24.8‰ to −22.4‰ (mean −23.4‰ ± 1.9) for δ^{13}C and from 2.8‰ to 6.9‰ (mean 4.5‰ ± 2.7) for δ^{15}N (see Table 3 for summary of the average values for animals). While there are no statistical differences in δ^{13}C, the difference in δ^{15}N between wild and domestic herbivores is significant ($t=4.61051052$; $df=55$; $p<0.001$) despite the small sample size of wild herbivores. One sheep among the herbivores (Bir-K68.01) exhibits an exceptionally high δ^{15}N value of 10.4‰, perhaps linked to the consumption of manured crops.

Fig 2. *Plot of δ^{13}C and δ^{15}N values of animals and humans by type of consumer.*

Table 3. *Summary data for δ^{13}C (‰ VPDB) and δ^{15}N (‰ AIR) of animals analysed in this study displayed by consumer type, domestic or wild animal type, period groups.*

	n	δ13C Range	δ13C Mean	δ13C SD	δ13C Median	δ15N Range	δ15N Mean	δ15N SD	δ15N Median
Herbivores (domestic)	49	-25.4 to -20.4	-22.8	1.1	-23.0	4.7 to 10.4	6.6	1.1	6.3
Herbivores (rural)	21	-24.6 to -20.6	-22.7	1.0	-22.9	5.3 to 10.4	7.0	1.3	7.1
Herbivores (urban)	28	-25.4 to -20.4	-22.9	1.1	-23.2	4.7 to 9.0	6.3	0.9	6.1

Herbivores (wild)	8	-24.8 to -22.4	-23.4	1.0	-23.0	2.8 to 6.9	4.5	1.3	4.2
Omnivores	27	-25.3 to -21.4	-23.0	0.8	-23.0	4.5 to 10.7	7.7	1.4	7.4
Carnivores	4	-23.9 to -21.6	-22.8	1.0	-22.8	8.7 to 9.3	9.0	0.3	9.0
Birds	10	-26.0 to -22.4	-23.6	1.0	-23.4	6.5 to 11.3	8.4	1.5	8.4
Fish	3	-28.2 to -27.6	-27.8	0.3	-27.8	10.9 to 13.5	12	1.3	11.6
Domestic fauna 11-13	51	-25.3 to -20.4	-22.6	1.0	-22.8	4.5 to 11.3	7.4	1.5	7.0
Domestic fauna 13-16	24	-25.4 to -21.2	-23.4	0.8	-23.3	4.7 to 9.9	6.8	1.3	6.7
Domestic fauna 16-18	12	-24.7 to -22.4	-23.2	0.7	-23.2	5.6 to 9.9	7.5	1.5	7.2
Urban domestic fauna	53	-25.4 to -20.4	-22.9	1.0	-22.9	4.5 to 10.7	7.1	1.4	6.9

Rural domestic fauna	34	-24.6 to -20.6	-23.0	0.9	-23.1	5.3 to 11.3	7.5	1.5	7.3

Omnivore samples (pig, n = 27) have $\delta^{13}C$ and $\delta^{15}N$ measurements ranging from −25.3‰ to −21.4‰ (mean −23.0‰ ± 1.6) and from 4.5‰ to 10.7‰ (mean 7.7‰ ± 2.9), respectively. Carnivores (cat and likely dogs) (n = 4) have $\delta^{13}C$ and $\delta^{15}N$ measurements ranging from -23.9‰ to −21.6‰ (mean −22.8‰ ± 1.9) and from 8.7‰ to 9.3‰ (mean 9.0‰ ± 0.5), respectively. Birds (n=10), mostly represented by chicken, but also geese and birds only defined to the level of 'galliformes' or wild bird, have $\delta^{13}C$ and $\delta^{15}N$ measurements ranging from -26.0‰ to −22.4‰ (mean −23.6‰ ± 1.9) and from 6.5‰ to 11.3‰ (mean 8.4‰ ± 2.9), respectively.

The fish (two pikes and one unidentified species) bone samples provide approximate $\delta^{13}C$ and $\delta^{15}N$ for available freshwater resources with $\delta^{13}C$ ranging from -28.2‰ to −27.6‰ (mean −27.8‰ ± 0.6) and $\delta^{15}N$ ranging from 10.9‰ to 13.5‰ (mean 12.0‰ ± 2.6). Freshwater, anadromous and marine species from studies conducted in Poland and Lithuania serve to complete the picture of potential food sources in this study (see Fig. 5).

When faunal $\delta^{13}C$ and $\delta^{15}N$ across sites are compared, only differences in $\delta^{13}C$ among herbivores appear to be significant based on analysis of variance ($F = 7.102$; $df = 3$; $p < 0.001$). However, the differences appear to be 'site-based' and do not simply fall along a rural and urban division. At the same time, differences in the herbivore $\delta^{15}N$

values, insignificant at the lower level of comparison, accumulate to be significant at the level of rural to urban sites comparison (t=2.342; df=33.568; p=0.025), with the $\delta^{15}N$ of rural herbivores being 0.8‰ higher than that of urban. Comparing the set of domestic animal samples by period shows that there are differences (F = 6.381; df = 2; p =0.003) in $\delta^{13}C$ values between the 11-13th centuries (n=51) and the 13-16th centuries (n=24), with the mean of the High Medieval fauna being 0.7‰ higher than that of the Late Medieval period (Fig. 3). The Early Modern sample size is limited but its mean $\delta^{13}C$ values are intermediate between the Late and High Medieval equivalents. Kruskal-Wallis test indicates no statistically significant differences in $\delta^{15}N$ values over the time periods (2.255; df = 2; p = 0.324).

Fig 3. *Human and animal samples compared by period.*

4.2 Human remains

Human bone collagen (n=143) has $\delta^{13}C$ ranging between -23.5‰ to −20.9‰ (mean = −22.1‰ ± 1.0) and $\delta^{15}N$ ranging between 5.8‰ and 11.9‰ (mean = 10.2‰ ± 1.7). Table S1 displays the complete data while Table 4 provides a summary of the average values, their range, and standard deviation. Overall, human values cluster rather tightly above the animals (including carnivores) on the $\delta^{15}N$ axis, apart from one (PUC20-G1) which has an exceptionally low $\delta^{15}N$ value of 5.8‰ (Fig. 2). If this value is excluded, the human range becomes -23.5‰ to −20.9‰ for $\delta^{13}C$ (mean = −22.1‰ ± 1.0) and $\delta^{15}N$ distance tightens to a range from 8.6‰ to 11.9‰ (mean = 10.3‰ ± 1.5). A linear regression model of $\delta^{13}C$ and $\delta^{15}N$ showed a very weak positive correlation between

these parameters for the sample of all humans ($R = 0.33$, $p < 0.001$), thus ^{13}C-enriched fish (either marine or anadromous) were not likely to have been a major source of protein in the diet.

Table 4. *Summary data for $\delta^{13}C$ (‰ VPDB) and $\delta^{15}N$ (‰ AIR) of humans analyzed in this study displayed by settlement type, period groups, burial context, sex.*

	n	$\delta^{13}C$ Range	$\delta^{13}C$ Mean	$\delta^{13}C$ SD	$\delta^{13}C$ Median	$\delta^{15}N$ Range	$\delta^{15}N$ Mean	$\delta^{15}N$ SD	$\delta^{15}N$ Median
**Total rural**	81	-23.5 to -21.1	-22.2	0.5	-22.2	8.6 to 11.9	10.0	0.8	10.1
Rural 11-13 cc	23	-23.5 to -21.5	-22.2	0.6	-22.2	8.7 to 11.3	10.1	0.7	10.1
Rural 13-16 cc	38	-23.3 to -21.5	-22.3	0.4	-22.4	8.6 to 11.9	9.8	0.7	9.7
Rural 16-18 cc	20	-22.9 to -21.1	-21.7	0.4	-21.7	9.3 to 11.9	10.6	0.7	10.5

**Total urban**	62	-23.2 to -20.9	-22.0	0.5	-22.0	5.8 to 11.6	10.4	0.9	10.6
Urban 11-13 cc	21	-23.2 to 21.1	-22.1	0.5	-22.1	5.8 to 11.3	10.1	1.1	10.2
Urban 13-16 cc	18	-23.0 to -21.9	-22.3	0.3	-22.3	9.1 to 11.3	10.3	0.6	10.3
Urban 16-18 cc	23	-22.9 to -20.9	-21.6	0.4	21.6	9.5 to 11.6	10.8	0.6	10.9
Elite	13	-22.4 to -20.9	-21.6	0.4	-21.6	9.5 to 11.6	10.6	0.7	10.8
Military	10	-22.9 to -21.0	-21.6	0.5	-21.5	10.4 to 11.5	11.0	0.4	11.2
**Total 11-13 cc**	44	-23.5 to -21.1	-22.2	0.6	-22.2	5.8 to 11.3	10.1	0.9	10.2

**Total 13-16 cc**	56	-23.3 to -21.5	-22.3	0.3	-22.3	8.6 to 11.9	9.9	0.7	9.9
**Total 16-18 cc**	43	-22.9 to -20.9	-21.7	0.4	-21.6	9.3 to 11.9	10.7	0.7	10.8
**Total male**	67	-23.5 to -21.0	-22.1	0.5	-22.0	8.6 to 11.9	10.3	0.7	10.4
**Total female**	61	-23.2 to -20.9	-22.0	0.5	-22.2	8.6 to 11.9	10.2	0.8	10.2

Human (n = 142) mean $\delta^{13}C$ values for periods of the 11-13th, 13-16th, and 16-18th centuries are −22.1‰ ± 1.0, −22.3‰ ± 0.6 and −21.7‰ ± 0.8 respectively. Mean $\delta^{15}N$ values from the earliest to the latest period are 10.2‰ ± 1.4, 9.9‰ ± 1.4, and 10.7‰ ± 1.4. For both $\delta^{13}C$ and $\delta^{15}N$, the values from the latest period of the 16-18th centuries are statistically different from both of the earlier two periods ($\delta^{13}C$: Kruskal-Wallis Statistic 47.696; df=2; $p<0.001$; $\delta^{15}N$: Kruskal-Wallis Statistic 24.276; df=2; $p<0.001$). The comparison of human bone collagen $\delta^{15}N$ between the urban cemeteries of Polack (n = 62) and the pooled sample of rural sites (n = 81) displays a significantly higher value

(10.4‰ ± 1.7 compared to 10.0‰ ± 1.6) for the urban group (U=1651; p<0.001), but no difference in $\delta^{13}C$ values (t=1.894; df=141; p=0.06). However, the difference in $\delta^{15}N$ only remains significant in the sample dated to the 13-16th centuries AD (t=2.766; df=54; p=0.008) when urban and rural groups are compared separately within each period.

The ranges of $\delta^{13}C$ and $\delta^{15}N$ within the nobility sub-sample (n=13) are from −22.4‰ to −20.9‰ (mean = −21.6‰ ± 0.7) and from 9.5‰ to 11.6‰ (mean = 10.6‰ ± 1.4), respectively (Table 4). The group of individuals identified as potentially having military affiliations (n=10) has $\delta^{13}C$ and $\delta^{15}N$ measurements ranging from -22.9‰ to −21.0‰ (mean = −21.6‰ ± 1.0) and from 10.4‰ to 11.5‰ (mean = 11.0‰ ± 0.8), respectively. The elite sub-sample thus has slightly higher $\delta^{13}C$ and lower, more variable $\delta^{15}N$ than the military sub-sample. The non-elite (rural) individuals from the 16-18th centuries (n=20) have $\delta^{13}C$ and $\delta^{15}N$ measurements ranging from -22.9‰ to −21.1‰ (mean = −21.7‰ ± 0.8) and from 9.3‰ to 11.9‰ (mean = 10.6‰ ± 1.4), respectively. Given that some temporal changes were already detected, status-based groups were compared only to contemporary commoners. Elite (n=13), military (n=10) and rural (n=20) human groups from the same time period of the 16-18th centuries showed no statistically significant differences either in $\delta^{13}C$ (Kruskal-Wallis Statistic 1.840; df=2; p=0.398) or in $\delta^{15}N$ (F=2.061; df=2; p=0.141). Interestingly, the elite subsample (n=13) showed some correlation between carbon and nitrogen values (R = 0.619, p=0.024), however.

Males (n = 67) and females (n = 61) present mean $\delta^{13}C$ values of −22.1‰ ± 1.1 and −22.0 ± 1.0‰ and mean $\delta^{15}N$ values of 10.3‰ ± 1.4 and 10.2‰ ± 1.6, respectively (Table 4 and Fig. 4). The t-test comparing sexes showed no evidence for statistically

significant differences for either $\delta^{15}N$ (t=1.046; df=126; p = 0.297) or $\delta^{13}C$ (t=0.799; df=126; p = 0.426) when the entire sample is considered together, and no differences are found between males and females buried in urban or rural cemeteries, or between sexes compared in each period separately.

Fig 4. *Plot of male and female $\delta^{13}C$ and $\delta^{15}N$ values.*

5.0 Discussion

5.1 Diet in Belarus between the 11th and 18th centuries AD

The terrestrial animal data presented here show $\delta^{13}C$ values typical for those feeding in C_3 environments. A comparison of omnivore $\delta^{13}C$ and $\delta^{15}N$ among sites and chronological periods indicates a general lack of environmental changes across time and space, though the herbivore sub-sample showed a slight, statistically significant increase in $\delta^{13}C$ between the groups of sites dated to the 11-13th and 13-16th centuries AD. Variation in environment or human management practices (e.g. millet foddering) between the geographic locations of sites (Biruli and especially Mienka are located to the south-west of the cluster of Polack, Lučna and Čarscviady, and have higher $\delta^{13}C$ values) or between the periods to which they date are among the possible explanations for this difference. However, more detailed study would be required to determine the reason with higher certainty. Rural herbivores also have a higher $\delta^{15}N$, possibly due to obtaining more of their fodder from manured fields. Interestingly, the sheep Bir-K68.01, which exhibits a $\delta^{15}N$ value of 10.4‰, was found in a kurgan. It is thus the only

animal in the dataset deposited in a human burial rather than a settlement context and is represented not by a single fragment but rather part of a whole skeleton. Such enrichment in ^{15}N may be due to extreme protein stress like starvation, or being fed a specific diet, or perhaps due to this individual coming from some distance away where local baseline $\delta^{15}N$ values were higher due to manuring [12]. Given the age-at-death of the animal is at least 1.5 years, the weaning effect is unlikely. Overall, the faunal data shows little change in the baseline $\delta^{13}C$ and $\delta^{15}N$ values across space and time, making them useful for interpreting human diets.

Most of the human samples fall within the range of -23.5‰ to −20.9‰, suggesting a clear reliance on C_3 terrestrial protein, which is consistent with the expected diets of Medieval European populations dominated by C_3 grains along with vegetables [77], and with the agricultural system prevalent on Belarusian lands in particular. The data in this study do not indicate the significant presence of millets in the diet in general, something supported by existing archaeological research [24,25]. Human $\delta^{15}N$ values consistent with high trophic position, especially among the peasants, are somewhat contradictory to the proposition that little meat and milk products were consumed by commoners. Given that the picture of meat appearing on the peasants' table on festive occasions was drawn primarily from ethnographic sources [21,26,27], which relate to the 19-20th centuries, this might suggest that animal protein before that time was actually more common in the diet of commoners. This is more in line with contemporary sources from neighbouring Medieval Poland, where meat was supposedly considered a basic food to be consumed on a daily basis even among the general populace [78]. A notable exception among human individuals is

the outlier PUC20-G1 with an exceptionally low $\delta^{15}N$ value of 5.8‰. This individual from Polack in the 11-13th centuries AD plots at the lower end of the herbivorous nitrogen values, which implies a vegetarian diet or substantial reliance on legumes [79]. Domestic species were likely the major source of animal protein for the majority of the population, as the offset between human $\delta^{15}N$ values and those of the wild herbivores is more than 5‰ and thus outside the expected trophic level increase. The fact that wild herbivores are on average 2.1‰ lower than domestic herbivores in terms of their $\delta^{15}N$ values is likely connected to domestic animals grazing on manured fields. Our data thus supports that manuring was likely utilized in the crop-rotation agricultural system at the time, also in the north of Belarus.

Another theme that was widely debated in terms of Medieval temporal trends was the adoption and growing role of Christianity, which in dietary terms would be expected to be reflected in a popularity of fish consumption. The data in this study do not support any significant reliance on aquatic resources, despite the assertion that fasts were kept very strictly in these lands [27], despite water resources being abundant in the north of Belarus [7,34], and despite fishing equipment appearing among archaeological finds here [33]. A similar low role of fish despite expectations connected with Christian fasts and the local availability of fish was observed in other non-elite sites, such as Alytus, Gruczno or Kałdus, as well as the coastal rural sites of Kretinga and Palanga in Lithuania [36]. The general lack of evidence for fish consumption may mean that the inhabitants of Belarusian lands were not so strict in observing Christian fasts or were participating in them in a different way than through focusing on fish consumption (e.g. nuts or berries, waterfowl and aquatic animals like

beaver, which were treated as fish-suitable for fast). However, freshwater fish are difficult to detect isotopically due to their wide ranges of δ^{13}C and δ^{15}N values, and δ^{13}C values of the human samples from Belarus do overlap both the anadromous fish ranges of -24‰ to -12‰ suggested in the literature [2] and δ^{13}C values of some of the Polish and Lithuanian fish samples, such as vimba, catfish, carp-bream and pike-perch - species caught in Belarus as well. Therefore, consumption of freshwater and anadromous fish cannot be completely excluded based on these data, either.

No evidence for statistically significant differences for both δ^{15}N and δ^{13}C were observed between males and females, either in the whole sample or within its subgroups based on urban or rural context or time period. Furthermore, the sub-sample of contemporary nobility from Polack showed no significant differences to peasants in terms of δ^{15}N values, contrary to the proposition of a drastic increase in the consumption of animal protein by the nobility after the 16th century under the Polish influence [42]. The only clear difference that the elite sub-sample shows relative to the rest of the individuals is a moderate correlation between the carbon and nitrogen values, perhaps suggesting consumption of anadromous or marine fish did play some role in elite isotopic variation. However, overall the lack of a significant increase in δ^{15}N value along social status lines suggests limited differences in animal protein access. One further explanation could be that the nobility were consuming greater amounts of wild animal protein (which seems to overall have lower δ^{15}N and δ^{13}C values in our study), which could offset increased animal protein use among elites and further highlight the significance of hunting as a sign of status in the region. Alternatively, limited variation in animal protein consumption between elites and

non-elites could emphasize the significance of the Christian fasting tradition, particularly for the Catholic confession to which this group of deceased is believed to belong, and use of fish and plants in the diet. Interestingly, the military sub-sample does seem to have higher $\delta^{15}N$ values than members of the elite, without any corresponding $\delta^{13}C$ trend, suggesting greater access to domestic animal protein or freshwater fish.

In terms of the comparison between city and village populations, we can see that urban or rural settings did not ~~have too much of~~ a major role in determining diet, at least at the resolution of isotopic analysis. Polack inhabitants have $\delta^{15}N$ values on average 0.5‰ higher than villagers. This small difference is significant only in the 13-16th centuries; however it is notable that at least the urban herbivores at this time have lower $\delta^{15}N$ values. A possible explanation is a slightly higher proportion of animal or freshwater fish protein in the urban diet. Considering that this is a more expensive source of energy, it might mirror the important economic role of Polack in reflecting the generally higher living standards and food security of its inhabitants. The urban/rural distinction probably became more visible between the 13th and 16th centuries as prior to this point urban and rural ways of life were very similar, with the majority of city dwellers engaged in agriculture and husbandry. Starting from the 13th century, however, the differentiation between the town and countryside increased. As feudal relations deepened, rural inhabitants concentrated on farming and only occasionally took part in trade relations. Given that in the 16th - 18th centuries this distinction between town and countryside was supposed to be deepening further, the lack of significant difference between rural and urban samples in this period may

reflect the decline of Polack due to constant military conflicts and raids on northern Belarus by Russian troops that became regular since the 1510s, and in particular the Livonian War of the second half of the 16th century - all of which resulted in the influx of rural population [9], and later even their purposeful relocation to the depopulated Polack region. Despite the initial proposition that urban diets could be more heterogeneous due to obtaining food from markets where not only strictly local but more distant and even foreign foods could be available, urban dwellers seem to, overall, cluster rather tightly, in a similar manner to the rural communities.

One of the major aims of this study was to investigate dietary change through time. In this regard, the only significant change was a slight increase in both carbon and nitrogen values after the 16th century. This suggests that, overall, the emergence of new states had little impact on the ordinary people, especially in rural areas. However, as the change seen following the 16th century cannot be explained by other parameters such as the environmental baseline (it showed a different pattern) or the variable nature of the samples dated to various periods (a similar pattern is preserved if rural and urban or male and female groups of individuals are compared separately by period), it is important to discuss what factors could have caused this shift. If the $\delta^{15}N$ values are simplistically interpreted as greater access to a higher trophic or aquatic protein source, then the slight increase of them in the time of the Polish-Lithuanian Commonwealth would be in line with the picture of the "Golden Age", not only for the highest strata of the society but also more broadly across the population. The simultaneous increase in $\delta^{13}C$ values in the period following the formation of the Polish-Lithuanian Commonwealth may also reflect the adoption of ^{13}C-enriched fish

into the diet by part of the population, which would be consistent both with the reported adoption of some marine imports into the menu of the nobility and with the striking reliance on such fish reported by Reitsema et al. [2] among Polish nobility of the same time period. Finally, the increase in human $\delta^{13}C$ values could indicate higher millet consumption in this period. Given that crop failures are often associated with the incorporation of millet, the bad harvests due to adverse weather events of 1600-1603 and the influence of the Little Ice Age in general, and the aforementioned constant military conflicts with Moscow starting from the early 16th century, which destroyed harvests among other things and undermined the economic contributions of the territory, might have played a role in the increase of millet consumption. The following century was similarly destructive for the GDL; for example, Alytus, another GDL city, was almost completely abandoned by 1712 following a series of depopulation events including an invasion by Sweden (1706), a nationwide famine (1708), and the Big Plague (1709–1711) which devastated populations not only in Lithuania but also in Poland and Prussia [5]. Nevertheless, a corresponding change in $\delta^{15}N$, which occurs in humans but not the animal baseline, suggests that more work is needed to clarify the exact cause of this change. Overall, human diets remain remarkably consistent between social groups and between time periods from an isotopic perspective.

5.2 Comparison with the surrounding region

We also compared our data with other geographically and chronologically proximate datasets available in the literature, namely from Poland [2,3] and Lithuania [5,36] - two countries with which Belarus formed a common state at different points in history. Unfortunately, the raw data from Yaroslavl [46] was not provided in the publication,

thus statistical comparison to the Russian territory is not possible, limiting us to general observations in this instance. The isotopic values for animals from all of the studies are shown in Figure 5. Only domestic herbivores had enough representation in studies from all three countries to allow for meaningful comparison through statistical tests. No difference was observed in $\delta^{15}N$ (Kruskal-Wallis Statistic 1.261; df=2; p=0.532), while stable carbon isotope values differed significantly between Belarus and the other two countries (Kruskal-Wallis Statistic 23.004; df=2; p<0.001). The means of domestic herbivores from Belarus (n=49), Lithuania (n=17), and Poland (n=8) were -22.8‰ ± 2.1, -22.1‰ ± 0.8, and -21.3‰ ± 0.9, respectively. As can be seen from Figure 3, omnivores, carnivores, and even birds and wild herbivores repeat the pattern where $\delta^{13}C$ values increase from Belarus, to Lithuania, to Poland, and it is statistically confirmed if the pooled samples of animals (excluding fish and marine mammals) are compared across these regions.

Fig 5. *Plots of herbivores, omnivores, carnivores with birds, and fish from the three countries (Belarus, Lithuania, Poland).*

Given that even wild herbivores exhibit this pattern, it is unlikely to be explained by human intervention. More plausible here is a difference in the environmental baseline affecting all animals, stemming from the plants consumed by herbivores and maintained along the whole trophic chain. Various characteristics of the local environment, such as water stress, precipitation, humidity, temperature, number of hours of sunshine, and salinity of soil influence ^{13}C abundance in C_3 plants

[80-83], causing 3–6‰ variation in their $\delta^{13}C$ values [80,83]. In particular, Somerville et al. [83] found a significant correlation between temperature and $\delta^{13}C$ values in the bone collagen of rabbits and hares, which in C_3-plant dominated environments may be due to C_3 plants exhibiting higher $\delta^{13}C$ values when water stressed relative to plants from cooler and wetter regions. This pattern seems to fit with the differences between countries compared in the current study, with Belarus being cooler and having more wet days than Lithuania, and Lithuania than Poland based on modern climatic data [84].

In terms of human data, all Belarusian sites (both rural and Polack) cluster quite distinctly from the Polish and Lithuanian data (Fig. 6). In terms of $\delta^{13}C$ values, Mann-Whitney U post-hoc tests show that Belarusian sites are significantly different to the others. Kałdus, a Medieval economic hub in Poland, is likewise separated from all but the Polish elites while other sites largely fall between them within a range of -21‰ and -19‰. The $\delta^{13}C$ distinction noted at Kałdus is likely due to substantial evidence for millet consumption reported based on stable isotope analyses [3]. Fish consumption was considered another contributing factor to higher $\delta^{13}C$ values, with fish providing a ready solution to the problem of feeding agglomerated populations detached from farming [3]. Human $\delta^{15}N$ across the different regions tends to be about one trophic level higher than associated animals, indicating significant consumption of terrestrial animal protein. Kruskal-Wallis tests indicate that Belarusian rural sites and Polack have similar $\delta^{15}N$ to the Polish site of Kałdus ($p=1.000$), Lithuanian rural sites ($p=1.000$) and Alytus ($p=0.313$ for Belarusian rural sites and $p=1.000$ for Polack sites). Kaldus was an economic centre of the 10-13th centuries AD in Poland where

inhabitants both produced and bought part of their food and where the animal protein in the diet came from domestic terrestrial animals complemented by fish [3]. Alytus was a small inland Medieval town in Lithuania, where most citizens engaged in agrarian occupations, such as agriculture, hunting, fishing, and animal husbandry, having a homogeneous diet based on C_3 terrestrial resources such as wheat, barley, cow, and pig [5]. Lithuanian rural sites refer to Medieval–Early Modern inland Kretinga and coastal Palanga, which both showed a high reliance on terrestrial resources and a diet based on cereals and legume crops and plant gathering (mostly mushrooms and nuts), supplemented by pork fat [36].

Fig 6. *Human samples from sites in Belarus, Lithuania and Poland.*

Lithuanian and in particular Polish elites, as well as the coastal site of Smeltė, stand out most distinctly from these other sites with higher $\delta^{15}N$ values. For the elites, this is explained by increased access to animal protein and freshwater and migratory fish consumption, which could have been connected to the elite consumer status of many fish (e.g. sturgeon), as well as the fact that many of these elites were related to the clergy [2]. This pattern is only partially repeated by the small group from Polack interpreted as burials of city nobility, who show some evidence of ^{13}C-enriched fish in the diet but no difference in the mean $\delta^{15}N$ values compared to the non-elite individuals. The sub-sample of nobility from Polack (mean $\delta^{13}C$= -21.6‰; mean $\delta^{15}N$=10.6‰) is significantly lower in $\delta^{13}C$ and $\delta^{15}N$ than the Lithuanian (mean $\delta^{13}C$ = -20.0‰; mean $\delta^{15}N$=11.6‰) and especially Polish elites (mean $\delta^{13}C$ = -19.5‰; mean

$\delta^{15}N$=13.6‰) from church burials ($\delta^{13}C$: Kruskal-Wallis Test Statistic 50.920; df=3; $p<0.001$; $\delta^{15}N$: Kruskal-Wallis Test Statistic 42.290; df=3; $p<0.001$). In this regard it should be noted that nobility in the Grand Duchy of Lithuania ranged from rather poor aristocracy to the most powerful and richest stratum of magnates who must have differed drastically in their lifestyles and diets. Unlike the Polish and Lithuanian elite samples, the sample considered to be nobility from Polack comes from a burial ground in the city center. Relatively higher reliance on hunting and consumption of wild animals meat on Belarusian lands could at least partially account for the lower nitrogen values of Polack nobility. Interestingly, according to Gurevich [41], hunting played an exceptional role in the cities of Black Rus, and in Navahrudak specifically - 36-58% of animal bones found there belong to wild species. However, such a significant role of hunting is not necessarily true for times after the Ancient Rus and for other cities of the Belarusian lands.

The non-elite samples from all regions do not show any clear evidence for fish consumption, apart from the modern coastal site of Smeltė in Lithuania, where diets seem to have included both terrestrial protein and considerable amounts of protein from freshwater fish. The 13th-century Russian city of Yaroslavl with its mean values of -20.0‰ for $\delta^{13}C$ and 11.8‰ for $\delta^{15}N$ (Fig. 6) was interpreted as demonstrating mixed diets based on plants from a temperate climate and meat from local terrestrial herbivores, where rare individuals with $\delta^{15}N$ values exceeding 13‰ probably regularly consumed freshwater fish [46]. This sample would plot much higher in both isotopic values than the Belarusian sites, and closest to the cluster of Lithuanian elites. It is particularly noteworthy given that the sample from Yaroslavl represents the full

population of a 13th-century city, not an elite subgroup from it. Overall, based on the examined samples, diets seem to have been dominated by C_3 plants and animal resources across this part of eastern Europe between the 11th and 18th centuries AD. Fish seems to have played a more marginal role than expected based on Christian fasting tradition, and was more easily accessed in urban or elite contexts unless rural sites were on the coast. In terms of elite groups, those from Belarus have lower $\delta^{15}N$ than those analyzed in Poland or Lithuania, perhaps indicating more similar diets to commoners than expected or the hunting and consumption of wild animals as a source of status. Future work, including single compound amino acid analysis, may help to definitively resolve this. The observed pattern does, however, stand in stark contrast to datasets from Medieval western Europe where clear isotopic distinctions between different elite and urban groups, and commoners, have been observed [4].

6.0 Conclusions

We present a new stable isotope dataset, assembled to explore how diets in the Polack region of Belarus changed throughout the 11-18th centuries in urban and rural settings, between male and female individuals, and across different burial settings. Overall, as is also seen from a comparison of data from the wider eastern European region, the diet in Medieval Belarus was based on C_3 terrestrial resources, among which cereals like rye and wheat, as well as vegetables played the major role. On average, a high access to animal protein for commoners is also observed. In general, the type of site (urban or rural) does not seem to have been a primary factor determining diet, with differences mainly centred around access to fish in inland

settings. The differences in diets between elite and commoners are similarly limited and probably due to religion or status-induced fish consumption across society, or maybe a higher role of hunting in the Belarusian context. Although more work is needed to further investigate the patterns observed in this study, our stable isotope analysis demonstrates how historical record can be tested in Medieval contexts, with eastern Europe providing an interesting and yet under-investigated comparison to other areas of Medieval Europe. Future research should focus on a more comprehensive reconstruction of dietary behaviours and variation by including individuals from broader geographic regions and time periods, as well as representing different strata of the society, such as magnates, clergy and individuals belonging to different confessions. Nevertheless, the data provides an important counterpoint to existing research from western Europe that appears to demonstrate more stark differences between rural and urban settings and between elite individuals and commoners [4].

CRediT authorship contribution statement

Vera Haponava: Conceptualization, Data curation, Formal analysis, Investigation, Methodology, Project Administration, Visualization, Writing - original draft, Writing - review & editing. **Aliaksei Kots**: Resources, Writing - review & editing. **Mary Lucas**: Methodology, Resources. **Max Both**: Investigation. **Patrick Roberts**: Conceptualization, Funding acquisition, Methodology, Supervision, Writing - review & editing. **Urszula Iwaszczuk**: Resources, Supervision. **Arkadiusz Sołtysiak**: Supervision. **D.S. Gritskevich**, **V.A. Yemieljanchyk**: Resources, Investigation. **A.V. Voytekhovich, M.V. Klimau, M.M. Pamazanau, O.V. Marfina:** Resources.

Acknowledgements

The authors express gratitude to Dr. Urszula Iwaszczuk (Institute of Mediterranean and Oriental Cultures, Polish Academy of Sciences) for her help with the identification of faunal species; to professor Arkadiusz Sołtysiak (Warsaw University) for supervision and advice; to D.S. Gritskevich and V.A. Yemieljanchyk from Polack State University (PSU) for determining the biological profiles of individuals from their university's osteological collection, as well as continuous aid in sample acquisition; to M.M. Pamazanau and O.V. Marfina from the Department of Anthropology, Institute of History of the National Academy of Sciences (NAS) of Belarus for help with organisational issues and for the access to materials; and to A.V. Voytekhovich (NAS) and M.V. Klimau (NAS) for the provisioning of animal materials and the related information. VH and PR would also like to thank the Max Planck Society for funding. VH would like to thank the University of Warsaw for additional funding support.

Appendix A. Supplementary data

Supplementary data to this article can be found online at https://docs.google.com/document/d/1s8u2HhESAo2k9j1JaNO-feoYKxNBjSo-VYd8tPWMRY8/edit.

Supplementary Note 1: The Polack region in the 11-18th centuries: historic background.

Supplementary note 2: Additional information on past diets in Belarus.

Supplementary Note 3: Further details about sites and contexts analysed.

Supplementary Table 1. The stable isotope results of the human samples and the quality control indicators.

Supplementary Table 2. The stable isotope results of the animal samples and the quality control indicators.

Supplementary References.

References

1. Reitsema LJ, Vercellotti G. Stable isotope evidence for sex- and status-based variations in diet and life history at medieval Trino Vercellese, Italy. American Journal of Physical Anthropology. 2012 Aug 1;148(4):589–600.

2. Reitsema L, Kozłowski T, Jankauskas R, Drążkowska Anna, Krajewska M. Dieta przedstawicieli elit społecznych Rzeczypospolitej na podstawie analizy stabilnych izotopów węgla i azotu w szczątkach szkieletowych. In: Kultura funeralna elit Rzeczypospolitej od XVI do XVIII wieku na terenie Korony i Wielkiego Księstwa Litewskiego [Internet]. Toruń: Wydawnictwo Naukowe Uniwersytetu Mikołaja Kopernika; 2015 [cited 2021 Nov 12]. p. 230–46. Available from: https://www.academia.edu/25091314/The_Diet_of_the_Polish_Lithuanian_Commonwealth_Social_Elites_on_the_Basis_of_Analyses_of_Stable_Carbon_and_Nitrogen_Isotopes_in_Skeletal_Remains

3. Reitsema LJ, Kozłowski T, Crews DE, Katzenberg MA, Chudziak W. Resilience and local dietary adaptation in rural Poland, 1000–1400 CE. Journal of Anthropological Archaeology. 2017;45:38–52.

4. Pérez-Ramallo P, Lorenzo-Lizalde JI, Staniewska A, Lopez B, Alexander M, Marzo S, et al. Stable isotope analysis and differences in diet and social status in northern Medieval Christian Spain (9th–13th centuries CE). Journal of Archaeological Science: Reports. 2022 Feb 1;41:103325.

5. Whitmore KM, Dupras TL, Williams LJ, Skipitytė R, Schultz JJ, Jankauskas R. Stable carbon and nitrogen isotope inter- and intra-individual dietary reconstruction from the late 14th to early 18th century site of Alytus, Lithuania. American Journal of Physical Anthropology. 2019 Feb 1;168(2):279–91.

6. Štýchaŭ HV. Goroda Polotskoy zemli (IX–XIII vv.). Minsk: Nauka i tekhnika; 1978. 160 p.

7. Halubovič VI. Ekanamičny stan, pobyt i handaĺ staražytnaj Bielarusi (IX—XIII stst.): dapamožnik dlia ekanamičnych spiecyjaĺnasciej vyšejšych navučaĺnych ustanoŭ. Minsk: Ekoperspektiva; 1997. 173 p.

8. Abiezhauz ZJ, Biejĺkin HJ, Buchaviec AR. Historyja sialianstva Bielarusi sa staražytnych časoŭ da našych dzion. Vol. 2. Minsk: Belaruskaia navuka; 2002. 552 p.

9. Duk DU. Polack i palačanie (IX-XVIII stst.) [Internet]. Navapolack: Polotsk State University; 2010 [cited 2021 Aug 5]. 180 p. Available from: http://elib.psu.by:8080/bitstream/123456789/608/1/duk_polotsk.pdf

10. Bialiavina VM. Ziemliarobstva. In: Narysy historyi kuĺtury Bielarusi U 4 t T 3 Kuĺtura siala CHIV – pačatku CHCH st Kn 1 Materyjaĺnaja kuĺtura. Minsk: Belaruskaia navuka; 2015. p. 353–415.

11. Lipatov AV. Velikoye knyazhestvo Litovskoye: istoricheskiy fenomen simbioza etnicheskikh kul’tur : (k voprosu o natsional’nykh putyakh vozvrashcheniya v nadnatsional’nuyu Yevropu). In: Etnokul’turnyye i etnoyazykovyye kontakty na territorii Velikogo Knyazhestva Litovskogo : materialy mezhdunarodnoy nauchnoy konferentsii. Moscow: Novoye izdatel’stvo; 2006. p. 6–19.

12. Lightfoot E, Slaus M, O’Connell TC. Changing Cultures, Changing Cuisines: Cultural Transitions and Dietary Change in Iron Age, Roman, and Early Medieval Croatia. Am J Phys Anthropol. 2012;148:543–56.

13. Halffman CM, Velemínský P. Stable isotope evidence for diet in early medieval Great Moravia (Czech Republic). Journal of Archaeological Science: Reports. 2015;2:1–8.

14. Tafuri MA, Craig OE, Canci A. Stable Isotope Evidence for the Consumption of Millet and Other Plants in Bronze Age Italy. Am J Phys Anthropol. 2009;139(2):146–53.

15. Müldner G, Montgomery J, Cook G, Ellam R, Gledhill A, Lowe C. Isotopes and individuals: Diet and mobility among the medieval Bishops of Whithorn. Antiquity [Internet]. 2009 Dec 1 [cited 2021 Jul 12];83. Available from: https://www.academia.edu/26011852/Isotopes_and_individuals_diet_and_mobility_among_the_medieval_Bishops_of_Whithorn

16. Lahtinen M, Salmi A-K. Mixed Livelihood Society in Iin Hamina – a Case Study of Medieval Diet in the Northern Ostrobothnia, Finland. null. 2019 Jan 2;24(1):1–14.

17. Titov VS. Otrazheniye entnosotsial′noy situatsii v zerkale statutov VKL. Dedy: daidzhest publikatsii o belaruskoi istorii. 2012;9:69–84.

18. Skiepjan AA. Arhanizacyja kuchonnaj služby pry mahnackich dvarach Vialikaha kniastva Litoŭskaha ŭ XVI—XVII ŭ stst. In: Mahnacki dvor i sacyjaĺnaje ŭzajemadziejannie (XV— XVIII stst) : zbornik navukovych prac. Mir: Muziej «Zamkavy komplieks «Mir»; 2014. p. 395–411.

19. Navahrodski TA. "Lítoŭskaya gaspadynya" kak istochnik po izucheniyu shlyakhetskoy kukhni na territorii Belarusi v pervoy polovine XIX v. In: Jeĺskija čytanni : materyjaly kanfierencyi "Šliachieckaja kuĺtura Bielarusi XIX — pačatku XX st." Dudutki: Knihazbor; 2013. p. 160–4.

20. Gurko AV, Bokhan LV, Bunkevich NS. Etnicheskiye traditsii natsional′noy kukhni Belarusi. Minsk: Belaruskaia navuka; 2019. 251 p.

21. Zinkievič E. Admietnasci narodnaj kuchni Braslaŭščyny i jaje suviaź z kaliendarom sviat. In: Narodnaja kuĺtura Viciebščyny : materyjaly navukova-praktyčnaj kanfierencyi "Pracesy madernizacyi narodnaj kuĺtury i vopyt ustanoŭ kuĺtury i adukacyi Viciebščyny pa zachavanniu tradycyj." Viciebsk: VDU imia P. M. Mašerava; 2007. p. 79–85.

22. Šamburova N. Admietnasci narodnaj kuchni Čašnickaha rajona i jaje suviaź z kaliendarom narodnych sviataŭ. In: Narodnaja kuĺtura Viciebščyny : materyjaly

navukova-praktyčnaj kanfierencyi "Pracesy madernizacyi narodnaj kuĺtury i vopyt ustanoŭ kuĺtury i adukacyi Viciebščyny pa zachavanniu tradycyj." Viciebsk: VDU imia P. M. Mašerava; 2007. p. 233–6.

23. Shcheglova V, Tkachov M, Zverugo Y, Duchits L. Materialy o zhivotnovodstve i okhote na territorii Severnoy Belarusi v sredniye veka. Histaryčna-Archiealahičny Zbornik. 2006;21:89–94.

24. Kiryanova NA. O sostave zemledel'cheskikh kul'tur Drevney Rusi X—XVvv. (po arkheologicheskim materialam). Sovetskaya arkheologiya. 1979;4:72–85.

25. Korobushkina TN. Zemledeliye na territorii Belorussii v X-XIII vv. [Internet]. Minsk: Nauka i tekhnika; 1979 [cited 2021 Jan 30]. 120 p. Available from: https://www.academia.edu/31532866/T_H_Коробушкина_Земледелие_на_территории_Белоруссии_в_X_XIII_вв_Zemědělství_v_Bělorusku_X_XIII_století_Agriculture_in_Belarus_in_the_X_XIII_centuries

26. Navahrodski TA. Evaliucyja tradycyj charčavannia bielarusaŭ u XIX—XX stst. Minsk: Bielaruski dziaržaŭny ŭniviersitet; 2015. 243 p.

27. Navahrodski TA. Narysy historyi kuĺtury Bielarusi U 4 t T 3 Kuĺtura siala XIV – pačatku XX st Kn 1 Materyjaĺnaja kuĺtura. Minsk: Belaruskaia navuka; 2015. p. 312–251.

28. Dovzhenok VY. Zemlerobstvo drevn'oyi Rusi. Do seredyny XIII st. Kyiv: Vydavnytstvo Akademiyi nauk URSR; 1961. 267 p.

29. Rakava LV. Aharodnictva. In: Narysy historyi kuĺtury Bielarusi U 4 t T 3 Kuĺtura siala CHIV – pačatku CHCH st Kn 1 Materyjaĺnaja kuĺtura. Minsk: Belaruskaia navuka; 2015. p. 416–55.

30. Rier YG. O razvitii zemledeliya v srednevekovoy Yevrope po arkheologicheskim dannym. MAB. 2016;28:61–4.

31. Koledinskiy LV. Vozdelyvaniye, uborka, pererabotka i khraneniye urozhaya v Vitebske v XIII—XVI vv. (po materialam raskopok Verkhnego zamka). MAB. 2017;28:87–100.

32. Kaspiarovič HI. Žyviolahadoŭlia ŭ majontkach. In: Narysy historyi kuĺtury Bielarusi: T 1: Kuĺtura sacyjaĺnaj elity XIV - pačatku XX st. Minsk: Belaruskaia navuka; 2013. p. 529–56.

33. Klimau MV, Lyashkevich EA. Da pytannia ab rybaloŭstvie na sieĺskich pasieliščach akruhi Polacka ŭ XI–XVI stst. MAB. 2015;26:50–8.

34. Kaspiarovič HI. Žyviolahadoŭlia. In: Narysy historyi kuĺtury Bielarusi U 4 t T 3 Kuĺtura siala CHIV – pačatku CHCH st Kn 1 Materyjaĺnaja kuĺtura. Minsk: Belaruskaia navuka; 2015. p. 456–91.

35. Gurevich FD. Sel′skoye khozyaystvo i promysly drevnerusskogo Novogrudka. Kratkiye soobshcheniya Instituta arkheologii. 1965;104:77–84.

36. Skipitytė R, Lidén K, Eriksson G, Kozakaitė J, Laužikas R, Piličiauskienė G, et al. Diet patterns in medieval to early modern (14th-early 20th c.) coastal communities in Lithuania 299-312. Anthropologischer Anzeiger. 2020;77(4):299–312.

37. Holubieŭ VF. Handaĺ u Vialikim Kniastvie Litoŭskim u XVI — XVIII stst.: stupień vyvučanasci, formy, pryncypy arhanizacyi, asnoŭnyja kirunki ŭ vyvučenni (pastanoŭka prabliemy). In: Historyja handliu na terytoryi Bielarusi: zbornik navukovych artykulaŭ. Minsk: Belaruskaia navuka; 2016. p. 21–32.

38. Gurevich FD. Vizantiyskiy import v gorodakh Zapadnoy Rusi v XII-XIII vv. Vizantiyskiy vremennik. 1986;47(72):65–81.

39. Žaryn DV. Hanziejskija kupcy ŭ Polacku ŭ XIII—XV stahoddziach. Byelaruski Gistarychny Chasopis. 2012;9:3–10.

40. Sorokovik IA. Vliyaniye "Dogovora Smolenskogo, Vitebskogo i Polotskogo knyazhestv s Rigoy, Gotlandskoy zemley i nemetskimi gorodami 1229 goda (Gotlandskaya redaktsiya)" na razvitiye belorusskoy torgovli. In: Historyja handliu ŭ Bielarusi (ad staražytnaha času da kanca XX st): prabliemy vyvučennia i pierspiektyvy dasliedavannia : materyjaly I Mižnarodnaj navukova-praktyčnaj kanfierencyi. Minsk: Technalohija; 2014. p. 68–73.

41. Gurevich FD. Drevniy Novogrudok: Posad — okol′nyy gorod. Leningrad: Nauka; 1981. 155 p.

42. Navahrodski TA. Tradycyi charčavannia pryviliejavanych sasloŭjaŭ Bielarusi. In: Narysy historyi kuĺtury Bielarusi: T 1: Kuĺtura sacyjaĺnaj elity XIV - pačatku XX st. Minsk: Belaruskaia navuka; 2013. p. 369–86.

43. Ambrose SH, Buikstra J, Krueger HW. Status and gender differences in diet at Mound 72, Cahokia, revealed by isotopic analysis of bone. Journal of Anthropological Archaeology. 2003 Sep 1;22(3):217–26.

44. Ambrose SH, Norr L. Experimental Evidence for the Relationship of the Carbon Isotope Ratios of Whole Diet and Dietary Protein to Those of Bone Collagen and Carbonate. In: Lambert JB, Grupe G, editors. Prehistoric Human Bone: Archaeology at the Molecular Level [Internet]. Berlin, Heidelberg: Springer Berlin Heidelberg; 1993. p. 1–37. Available from: https://doi.org/10.1007/978-3-662-02894-0_1

45. Reitsema LJ, Kozłowski T. Diet and society in Poland before the state: Stable isotope evidence from a Wielbark population (2nd c. AD). Anthropological Review. 2013;76(1):1–22.

46. Engovatova AV, Dobrovolskaya MV, Antipina II, Zaitseva GI. Kollektivnyye zakhoroneniya v Yaroslavle. Rekonstruktsiya sistemy pitaniya na osnove rezul'tatov izotopnogo analiza. Kratkiye soobshcheniya Instituta arkheologii. 2013;228(96–115).

47. Charauko VU. Archiealahičnaje vyvučennie mohiĺnika kalia v. Ivieś Hlybockaha rajona ŭ 2011 h. In: Materyjaly Mižnarodnaj navukovaj kanfierencyi (Polack, 22-23 maja 2012 h) [Internet]. Minsk: Belaruskaia navuka; 2012 [cited 2021 Jun 19]. p. 417–26. Available from: http://elib.psu.by:8080/handle/123456789/15261

48. Yemialyanchyk VA. Antropologicheskiy sostav srednevekovogo sel'skogo naseleniya Polotskoy zemli po dannym kraniologii. Vestnik antropologii. 2012;(22):161–73.

49. Charauko VU. Pachavaĺny abrad pozniesiarednieviakovaha mohiĺnika kalia v. Ivieś Hlybockaha rajona Viciebskaj voblasci (pa materyjalach archiealahičnych dasliedavanniaŭ u 2010—2015 hh.). In: Historyja i archiealohija Polacka i Polackaj ziamli : materyjaly VII Mižnarodnaj navukovaj kanfierencyi [Internet]. Polack: Nacyjanaĺny Polacki historyka-kuĺturny muziej-zapaviednik; 2019 [cited 2021 Oct 10]. p. 325–37. Available from: http://elib.psu.by:8080/handle/123456789/23753

50. Štýchaŭ HV. Kryvičy: pa materyjalach raskopak kurhanoŭ ŭ paŭnočnaj Bielarusi. Minsk: Navuka i technika; 1992. 191 p.

51. Dučyc LU, Kviatkoŭskaja AV. Pachavaĺnyja pomniki navakolic Padsvillia. Histaryčna-Archiealahičny Zbornik. 1994;4:58–70.

52. Tarasaŭ SV, Dučyc LU, Siemiančuk HM. Kurhanny mohiĺnik kalia v. Dzmitraŭščyna Polackaha rajona. Histaryčna-Archiealahičny Zbornik. 1995;6:230–41.

53. Dučyc LU. Kurhanny mohiĺnik Ziabki-Douhaje. Histaryčna-Archiealahičny Zbornik. 2001;16:111–4.

54. Siemiančuk HM. Novaja katehoryja archiealahičńich pomnikaŭ na terytoryi i Polackaj ziamli (hruntovyja mahiĺniki X-XIII stst.). In: Tezisy dakladaŭ navukovaj kanfierencyi, prysviečanaj 1130-hoddziu Polacka [Internet]. Polack: Polacki historyka-kuĺturny zapaviednik; 1992 [cited 2021 Jun 18]. p. 53–6. Available from: http://polotsk.museum.by/node/34118

55. Plavinski MA. Kurgan 5 mogil’nika Pogoshcha kak primer elitarnogo pogrebal’nogo kompleksa kontsa KH — nachala XI v. na severo-zapade Polotskoy zemli. In: Drevnosti Issledovaniya Problemy. Kishinev-Tiraspol: Izdatel’skiy dom Stratum; 2018. p. 445–64.

56. Charauko VU. Archiealahičnaje vyvučennie hruntovych pachavanniaŭ XVI–XVIII stahoddziaŭ na mohiĺniku kalia v. Klieščyno Biešankovickaha rajona ŭ 2013 hodzie. Vestnik Polotskogo gosudarstvennogo universiteta. 2015;9:84–9.

57. Charauko VU. Archiealahičnyja raskopki na pozniesiarednieviakovym mohiĺniku kalia v. Michalinava Lioznienskaha rajona ŭ 2016 h. Vestnik Polotskogo gosudarstvennogo universiteta. 2021;1:200–5.

58. Charauko VU. Arkheologicheskiye issledovaniya na mogil’nike vozle d. Dubrovka Gorodokskogo rayona v 2014 g. Vestnik Polotskogo gosudarstvennogo universiteta. 2016;1:153–61.

59. Charauko VU. Archiealahičnyja raskopki na mohiĺniku kalia vioski Doŭhaje Hlybockaha rajona ŭ 2015 h. In: Zbornik navukovych artykulaŭ III mižnarodnaj navukovaj kanfierencyi (Polack, 14–15 krasavika 2016 h): u 2 častkach [Internet]. Navapolack: Polacki dziaržaŭny univiersitet; 2016 [cited 2021 Jun 19]. p. 170–7. Available from: http://elib.psu.by:8080/handle/123456789/19637

60. Vajtsjakhovich AV. Asablivasci pachavaĺnaha abradu kurhannaha mohiĺnika Biruli (Parečča) Dokšyckaha rajona Viciebskaj vobl. MAB. 2008;15:218–35.

61. Charauko VU. Archiealahičnyja dasliedavanni na kurhanna-žaĺničnym mohiĺniku kalia v. Biruli Dokšyckaha rajona ŭ 2011 h. In: Materyjaly navukovych kanfierencyj [Internet]. Minsk: Nacyjanaĺnaja biblijateka Bielarusi; 2013 [cited 2021 Jun 18]. p. 24–5. Available from: http://elib.psu.by:8080/handle/123456789/819

62. Kviatkoŭskaja AV. Otchet o provedenii arkheologicheskikh raskopok u d. Volcha i u d. Osetishche Dokshitskogo rayona. Minsk: National Academy of Sciences of Belarus; 1991 p. 27. Report No.: 1233a.

63. Kviatkoŭskaja AV. Dasliedavannie kamiennych mahil kalia v. Vaŭča Dokšyckaha i v. Pieravoz Hlybokskaha rajonaŭ. In: Tezisy dakladaŭ navukovaj kanfierencyi, prysviečanaj 1130-hoddziu Polacka [Internet]. Polack: Polacki historyka-kuĺturny zapaviednik; 1992 [cited 2021 Jun 18]. p. 30–2. Available from: http://polotsk.museum.by/node/34118

64. Duk DV, Yemialyanchyk VA, Kots AL. Mohilki na strelcy Nižniaha zamka ŭ Polacku. MAB. 2018;29:171–82.

65. Duk DU, Yemialyanchyk VA. Mohilki polackaha haradzišča (pa vynikach archiealahičnych raskopak u 2007 h.). MAB. 2011;21:61–73.

66. Reitz EJ, Wing ES. Zooarchaeology. 2nd ed. Cambridge: Cambridge University Press; 2008. 560 p. (Cambridge Manuals in Archaeology).

67. Gifford-Gonzalez D. An Introduction to Zooarchaeology. Switzerland: Springer International Publishing AG; 2018. 604 p.

68. Chesson L, Meier-Augenstein W, Berg G, Bataille C, Bartelink E, Richards M. Basic principles of stable isotope analysis in humanitarian forensic science. In 2020. p. 285–310.

69. Scheuer L, Black S. CHAPTER FIVE - The Head, Neck and Dentition. In: Scheuer L, Black S, editors. Developmental Juvenile Osteology [Internet]. London: Academic Press; 2000. p. 36–170. Available from: https://www.sciencedirect.com/science/article/pii/B978012624000950006X

70. Buikstra JE, Ubelaker DH. Standards for data collection from human skeletal remains. Fayetteville: Arkansas Archeological Survey; 1994. 272 p. (Arkansas Archeological Survey Research Series).

71. Piontek J. Biologia populacji pradziejowych: zarys metodyczny. Poznań: Wydawnictwo Naukowe UAM; 1996. 217 p.

72. Herrmann B, Grupe G, Hummel S, Piepenbrink H, Schutkowski H. Prähistorische Anthropologie: Leitfaden der Feld- und Labormethoden. Berlin Heidelberg: Springer-Verlag; 1990. 454 p.

73. Brothwell DR. Digging up bones : the excavation, treatment and study of human skeletal remains. 3rd ed.. British Museum; 1981.

74. White TD, Black MT, Folkens PA. Human Osteology. 3rd ed. Academic Press; 2012. 662 p.

75. Richards MP, Hedges REM. Stable Isotope Evidence for Similarities in the Types of Marine Foods Used by Late Mesolithic Humans at Sites Along the Atlantic Coast of Europe. Journal of Archaeological Science. 1999 Jun 1;26(6):717–22.

76. van Klinken GJ. Bone Collagen Quality Indicators for Palaeodietary and Radiocarbon Measurements. Journal of Archaeological Science. 1999 Jun 1;26(6):687–95.

77. Reitsema LJ, Crews DE, Polcyn M. Preliminary evidence for medieval Polish diet from carbon and nitrogen stable isotopes. Journal of Archaeological Science. 2010 Jul 1;37(7):1413–23.

78. Dembińska M, Weaver WW. Food and drink in medieval Poland : rediscovering a cuisine of the past [Internet]. 1999 [cited 2021 Sep 18]. Available from: http://www.reenactor.ru/ARH/PDF/Dembinska.pdf

79. Katzenberg MA. Stable Isotope Analysis: A Tool for Studying Past Diet, Demography, and Life History. In: Biological Anthropology of the Human Skeleton [Internet]. John Wiley & Sons, Ltd; 2008. p. 411–41. Available from: https://onlinelibrary.wiley.com/doi/abs/10.1002/9780470245842.ch13

80. Cormie AB, Schwarcz HP. Effects of climate on deer bone δ15N and δ13C: Lack of precipitation effects on δ15N for animals consuming low amounts of C 4 plants. GCA. 1996 Nov;60(21):4161–6.

81. Ferrio JP, Voltas J, Alonso N, Araus J. Reconstruction of Climate and Crop Conditions in the Past Based on the Carbon Isotope Signature of Archaeobotanical Remains. In: Terrestrial Ecology. 2007. p. 319–32.

82. Panek JA, Waring RH. Stable carbon isotopes as indicators of limitations to forest growth imposed by climate stress. Ecological Applications. 1997;7:854–63.

83. Somerville AD, Froehle AW, Schoeninger MJ. Environmental influences on rabbit and hare bone isotope abundances: Implications for paleoenvironmental research. Palaeogeography, Palaeoclimatology, Palaeoecology. 2018 May 15;497:91–104.

84. Fao.org. 2022. *https://www.fao.org/aquastat/en/*. [online] Available at: <https://www.fao.org/aquastat/en/> [Accessed 23 March 2022].

Click here to access/download
Supporting Information
Haponava et al. - Supplementary Information.docx

Figures 1-6 (zipped)

Click here to access/download
Supporting Information - Compressed/ZIP File Archive
Haponava et al. - Figures.7z
